# Efficacy and morbidity of biodegradable versus titanium osteosyntheses in orthognathic surgery: A systematic review with meta‐analysis and trial sequential analysis

**DOI:** 10.1111/eos.12800

**Published:** 2021-06-15

**Authors:** Barzi Gareb, Nico B. van Bakelen, Pieter U. Dijkstra, Arjan Vissink, Ruud R. M. Bos, Baucke van Minnen

**Affiliations:** ^1^ Department of Oral and Maxillofacial Surgery University Medical Center Groningen University of Groningen Groningen the Netherlands; ^2^ Department of Rehabilitation Medicine University Medical Center Groningen University of Groningen Groningen the Netherlands

**Keywords:** absorbable implant, biocompatible materials, orthopedic fixation devices, osteotomy, polymers

## Abstract

Titanium osteosynthesis is currently the gold standard in orthognathic surgery. Use of biodegradable osteosyntheses avoids removal of plates/screws in a second operation. This systematic review aimed to assess the efficacy and morbidity of biodegradable vs. titanium osteosyntheses in orthognathic surgery (PROSPERO CRD42018086477). Patients with syndromic disorder(s) and/or cleft lip/palate were excluded. Randomised, prospective and retrospective controlled studies were searched for in nine databases (February 2021). The time periods perioperative, short‐term, intermediate, long‐term, and overall follow‐up were studied. Meta‐analyses were performed using random‐effects models. A total of 9073 records was assessed, of which 33 were included, comprising 2551 patients. Seven RCTs had ‘some concerns’ while another seven RCTs had ‘high’ risk of bias (Cochrane‐RoB2). No differences in malunion (qualitative analyses), mobility of bone segments [RR 1.37 (0.47; 3.99)], and malocclusion [RR 0.93 (0.39; 2.26)] were found. The operative time was longer in the biodegradable group [SMD 0.50 (0.09; 0.91)]. Symptomatic plate/screw removal was comparable among both groups [RR 1.29 (0.68; 2.44)]. Skeletal stability was similar in most types of surgery. Biodegradable osteosyntheses is a valid alternative to titanium osteosyntheses for orthognathic surgery, but with longer operation times. Since the quality of evidence varied from very low to moderate, high‐quality research is necessary to elucidate the potential of biodegradable osteosyntheses.

## INTRODUCTION

Titanium osteosynthesis systems are currently the fixation systems of choice in orthognathic surgery. The disadvantages, though, include temperature sensitivity, [1] tactile sensation of plates and screws, [2] growth restrictions, [3] hampering of imaging and radiotherapy, [4, 5, 6] presence of titanium particles in lymph nodes, [7] extreme stiffness causing stress shielding of the underlying bone, [6] and potential mutagenicity [1]. Hence, titanium systems are removed in up to 33% of the cases with accompanying costs and burdens [2, 8].

Biodegradable plates and screws are composed of degradable polymers (e.g. poly‐L‐lactic or polyglycolic acid) and may reduce removal rates of these osteosynthesis systems in a second operation while also avoiding the disadvantages of titanium osteosyntheses. Biodegradable systems have, however, their own limitations including lower strength and stiffness [9] that could lead to higher malunion rates and less skeletal stability after orthognathic surgery, palpability due to bulkiness, [10] and possible foreign body reactions [11]. As a consequence, biodegradable implants are removed in up to 17% of the cases in a second operation [2, 10, 12].

Studies comparing biodegradable vs. titanium osteosyntheses after orthognathic surgery have been performed, but the results are conflicting. Some studies report higher plate removal rates after titanium osteosyntheses, [13, 14, 15, 16] while other studies [2, 17, 18] show higher rates of symptomatic plate removal after biodegradable osteosyntheses. This controversy also applies to other clinically assessed endpoints such as skeletal stability [14, 19, 20] and pain [2, 14]. In 2017, a systematic review focussed on the safety and efficacy of biodegradable vs. titanium systems [21] and included two randomised controlled trials (RCTs) but could not perform a meta‐analysis. The authors concluded that there was insufficient evidence as to which osteosynthesis system is superior [21, 22]. However, certain RCTs that were available at the time of writing were, for unknown reasons, not included in the review. In 2018, a systematic review comparing osteosynthesis systems for orthognathic surgery was published [23] but focused on skeletal stability only and failed to account for clinical (e.g. inclusion of patients with cleft lip and palate) and methodological heterogeneity (e.g. they pooled the data from different study designs). Thus, to guide evidence‐based decisions, the need remains for a systematic review of the current literature that assesses the efficacy and morbidity of biodegradable vs. titanium osteosyntheses in patients undergoing orthognathic surgery adequately, including all relevant clinical endpoints (i.e. not only restricted to skeletal stability) and which takes methodological and clinical heterogeneity of the studies into account.

This systematic review with meta‐analysis and trial sequential analysis was performed to analyse the efficacy [i.e. bone healing, (mal)occlusion, skeletal stability] and morbidity of biodegradable (i.e. consisting of synthetic polymers) against titanium osteosyntheses in patients with dentofacial deformities treated with orthognathic surgery.

## MATERIAL AND METHODS

This systematic review and meta‐analysis was conducted following the recommendations of the *Cochrane Handbook for Systematic Reviews of Interventions* and reported according to the *Preferred Reporting Items for Systematic Reviews and Meta*‐*Analyses (PRISMA)* statement [24, 25]. The study protocol was registered in PROSPERO prior to the systematic literature search (registration number CRD42018086477). A systematic review of biodegradable vs. titanium osteosyntheses in trauma patients, using the same approach, was published separately [10].

### Study identification

A systematic literature search of nine electronic databases [PubMed, EMBASE, the Cochrane Central Register of Controlled Trials (CENTRAL), Web of Science, EBSCOhost, Scopus, African Journals Online, OpenGrey and ClinicalTrials.gov] was conducted (all: inception to 2021). The sensitive search strategy consisted of medical subject heading terms and free‐text words (Table S1). The search strategy also included maxillofacial trauma populations as some studies include both populations in a single study. Data of patients treated with orthognathic surgery were derived from the authors of those studies and included while the trauma patients’ data were excluded for this paper but were recently published separately [10]. The initial search was performed on January 29, 2018, and was updated on February 11, 2021. Additionally, the reference lists of the included studies and leading oral and maxillofacial journals were screened for relevant studies. Maxillofacial surgery experts in biodegradable and titanium osteosynthesis (R.R.M.B. and N.B.vB.) were asked if any relevant studies were missing. No language or period restrictions were applied.

### Study selection

Inclusion criteria were formulated using the PICOS format. The population (P) included patients with dentofacial deformities treated with orthognathic surgery i.e. Le Fort I, Le Fort II, Le Fort III, (bilateral) sagittal split (BSSO), and intraoral vertical ramus osteotomies (IVRO), with and without concurrent genioplasty. The intervention group (I) was treated surgically with biodegradable fixation (i.e. plates and/or screws/pins) that consisted of (co‐)polymers. The control group (C) was surgically treated with titanium fixation (i.e. plates and/or screws). The primary outcome (O) was efficacy of the fixation method, i.e. adequate bone healing with the absence of malunion of bone segments at 12 weeks follow‐up. The secondary outcomes were related to morbidity, i.e. clinical mobility of bone segments, objective and subjective malocclusion, symptomatic osteosynthesis device removal rate (i.e. routinely removed asymptomatic plates were excluded), skeletal stability (i.e. skeletal relapse) assessed by lateral cephalograms or three dimensional imaging, pain, analgesic usage, maximal mouth opening, mandibular function impairment questionnaire (MFIQ; lower score equals better function), temporomandibular joint dysfunction (TMJ‐dysfunction), infection, swelling, wound dehiscence, plate exposure, palpability of plates and/or screws, the patient's satisfaction with the performed surgery, and revision surgery other than device removal (e.g. abscess incision and drainage). Additionally, the handling of the osteosynthesis systems by the surgeons, plate and screw breakage, operative time, and total costs (i.e. direct and indirect costs) of both groups were evaluated [10]. The included study types (S) were RCTs, prospective studies with a control group, and retrospective studies with a control group. RCTs are the highest quality of evidence of an original study, while the latter two designs are valuable for, e.g. the assessment of adverse events. The follow‐up of each corresponding endpoint is described below (see Data collection) [10].

Exclusion criteria consisted of patients with syndromic disorder(s), patients with cleft lip or palate, multiple publications of the same study and endpoints, case reports, case series with fewer than 10 cases, experts’ opinions, letters to the editor, review articles, and conference abstracts [10].

Two reviewers (B.G. and N.B.vB.) independently assessed titles and abstracts for inclusion eligibility. If the title and abstract provided insufficient information, or in case of any doubt, full‐text assessment followed. The full text of the included titles and abstracts were independently assessed by the same two reviewers for final inclusion using the above‐mentioned in‐ and exclusion criteria. Any disagreement was resolved by a discussion. If no consensus could be reached, a third reviewer (P.U.D.) was available to make a final decision. After each selection stage, inter‐observer reliability was expressed as Cohen's kappa and percentage of agreement. Studies written in languages that the observers were not competent in were translated to English by researchers fluent in both that language and English. Subsequently, these translated studies underwent the same review process [10].

### Data collection

The data were extracted using a standardised, pre‐defined form [10]. Two reviewers (B.G. and N.B.vB.) extracted data from a sample (10%) of eligible studies. If agreement was ≥80%, the remainder of the data were extracted by one reviewer (B.G.). The collected data included: first author and year of publication, country in which the study was conducted, study design, number of patients, gender, age, tobacco and alcohol usage, surgical procedures, types of osteosynthesis systems used, intra‐operative switching to another osteosynthesis system, duration of postoperative maxillomandibular fixation, duration of orthodontic treatment, postoperative dietary restrictions, duration of follow‐up, and conflict of interests. The endpoints were collated for five time periods: perioperative, short‐term follow‐up (i.e. 0–4 weeks; soft tissue healing), intermediate follow‐up (i.e. 6–12 weeks; bone healing), long‐term follow‐up (i.e. >12 weeks; degradation effects), and overall follow‐up (i.e. the endpoints of the longest follow‐up; Table S2) [10]. If an identical endpoint was assessed in multiple articles of the same study (e.g. same RCT) at different follow‐ups, the article with the longest follow‐up was included in the analyses of that specific endpoint.

Lateral cephalograms and three‐dimensional imaging were used to assess skeletal stability. The landmarks used for maxillary horizontal and vertical relapse were, in order of priority: point A, anterior nasal spine (ANS), and the anterior implant (AI). Maxillary angular relapse was assessed using the angle sella‐nasion‐point A (SNA). The landmarks used for mandibular horizontal and vertical relapse were, in order of priority: point B, pogonion (Pg), and menton (Me). Mandibular angular relapse was assessed using the angle articulare‐gonion‐gnathion.

The skeletal stability data were included if the first postoperative cephalogram was performed within one month after surgery. The amount of relapse was assessed by calculating the difference between the measurements of the longest follow‐up cephalogram and the first postoperative cephalogram. Whenever the differences in measurements were not presented in the manuscript, but the data of those two time points (i.e. first postoperative and latest cephalogram) were given as means and standard deviations, the mean and standard deviation of the difference was calculated assuming a normal distribution of data. Data presented as medians with (interquartile) ranges were excluded. All the skeletal stability data were converted to absolute values for statistical analyses.

Whenever two or more studies included an identical control group, the means and standard deviations of both intervention groups were pooled and analysed as a single pair‐wise comparison with that specific control group, assuming normal distribution of data, as recommended in the *Cochrane Handbook for Systematic Reviews of Interventions* [25].

If the relevant data could not be extracted, the authors of the studies were contacted by e‐mail from May–November 2018 and April–July 2019. Data were not included in the analyses if the authors did not respond despite three email attempts (Table S3) [10].

### Risk of bias assessment

The risk of bias of all the included studies was independently assessed by the two reviewers (B.G. and N.B.vB.). Trials performed by the author's research group were assessed by two independent researchers not involved in those studies (P.U.D and S.J.vdG.; see acknowledgement) to avoid conflict of interests [10].

Randomised controlled trials were assessed using *The Revised Cochrane risk*‐*of*‐*bias tool* (RoB 2) [26]. The domains were graded low risk, some concerns, or high risk of bias. The nonrandomised studies’ risk of bias was assessed using the Methodological Index for Non‐Randomized Studies (MINORS) [27]. The MINORS is a valid and reliable instrument for bias assessment [27]. Each item was scored either 0 (not reported), 1 (reported but inadequate), or 2 (reported and adequate).

The quality of the body of evidence for each outcome was graded by the two independent reviewers (B.G. and N.B.vB.) as high, moderate, low, or very low quality using the *Grades of Recommendation*, *Assessment*, *Development and Evaluation Working Group system* (GRADE system) [28].

### Statistical analysis

The inter‐observer agreement was calculated using IBM spss Statistics 23 (SPSS). Regarding binary variables, the events and totals were used to calculate the risk ratio (RR) and 95% confidence intervals (CI). The standardised mean difference (SMD), with 95% CI, was calculated for the continuous variables. Statistical heterogeneity was regarded substantial if *I^2^* > 50% [25]. Separate analyses were conducted of the study designs. A summary effect estimate was calculated if ≥2 studies with the same study design could be pooled, unless these studies were total zero‐event studies [25]. The meta‐analysis was performed in R‐*meta* (R v4.0.2, *meta*‐package v4.15‐1), using a random‐effects model with the DerSimonian‐Laird estimator, due to the presence of clinical heterogeneity [25, 29].

The following a priori defined subgroup analyses of the primary outcome, malocclusion, and device removal rate were performed using random‐effects model with the DerSimonian‐Laird estimator: low vs. high risk bias RCTs, paediatric patients (<16 years) vs. adults, osteosyntheses with plates and screws/pins vs. only screws/pins, ≤8 mm vs. >8 mm mandibular advancement, and mandibular vs. maxillary osteotomies. Device removal rate was also analysed according to the follow‐up of the included studies, i.e. ≤1‐year follow‐up and >1‐year follow‐up. Additionally, subgroup analyses of the skeletal stability following osteosyntheses with plates and screws/pins vs. only screws/pins and ≤8 mm vs. >8 mm mandibular advancement were performed.

Since conventional meta‐analyses exclude studies with zero events in both treatment groups, a sensitivity analysis was performed, including those studies with a reciprocal continuity correction of the opposite arm [30]. Furthermore, a sensitivity analysis comparing the effect estimates of all included RCTs vs. non‐high‐risk‐of‐bias RCTs was performed. A meta‐regression analysis with a random‐effects model with the DerSimonian‐Laird estimator evaluated the effect of the study design and the risk of bias items for the primary endpoint, mobility of bone segments, malocclusion, and symptomatic device removal. The meta‐regression was conducted using R‐*meta*. Reporting bias was assessed through funnel plots if >10 studies were available per endpoint and study design, and did not have clinical heterogeneity [10, 25, 31, 32, 33].

As traditional meta‐analyses are prone to type‐I errors (i.e., false positive findings) due to random error and repeated significance testing after each additional trial is published, [34, 35] trial sequential analyses (TSA), including RCTs, were performed for each binary endpoint. An explanation of the TSA, with an example and interpretation of the data, is shown in Figure S1. The TSA, which included the random‐effects (DerSimonian‐Laird estimator) model based on the observed relative risk reduction (RRR) and diversity (D^2^) of RCTs, and an overall type I error (α) of 0.05 and a type II error (β) of 0.20, [36] was performed using trial sequential analysis viewer, version 0.9.5.1 beta (Copenhagen Trial Unit, Centre for Clinical Intervention Research, Rigshospitalet) [10, 36].

In all analyses, *p* < 0.05 (two‐tailed) was considered statistically significant.

## RESULTS

### Study identification and selection

**FIGURE 1:**
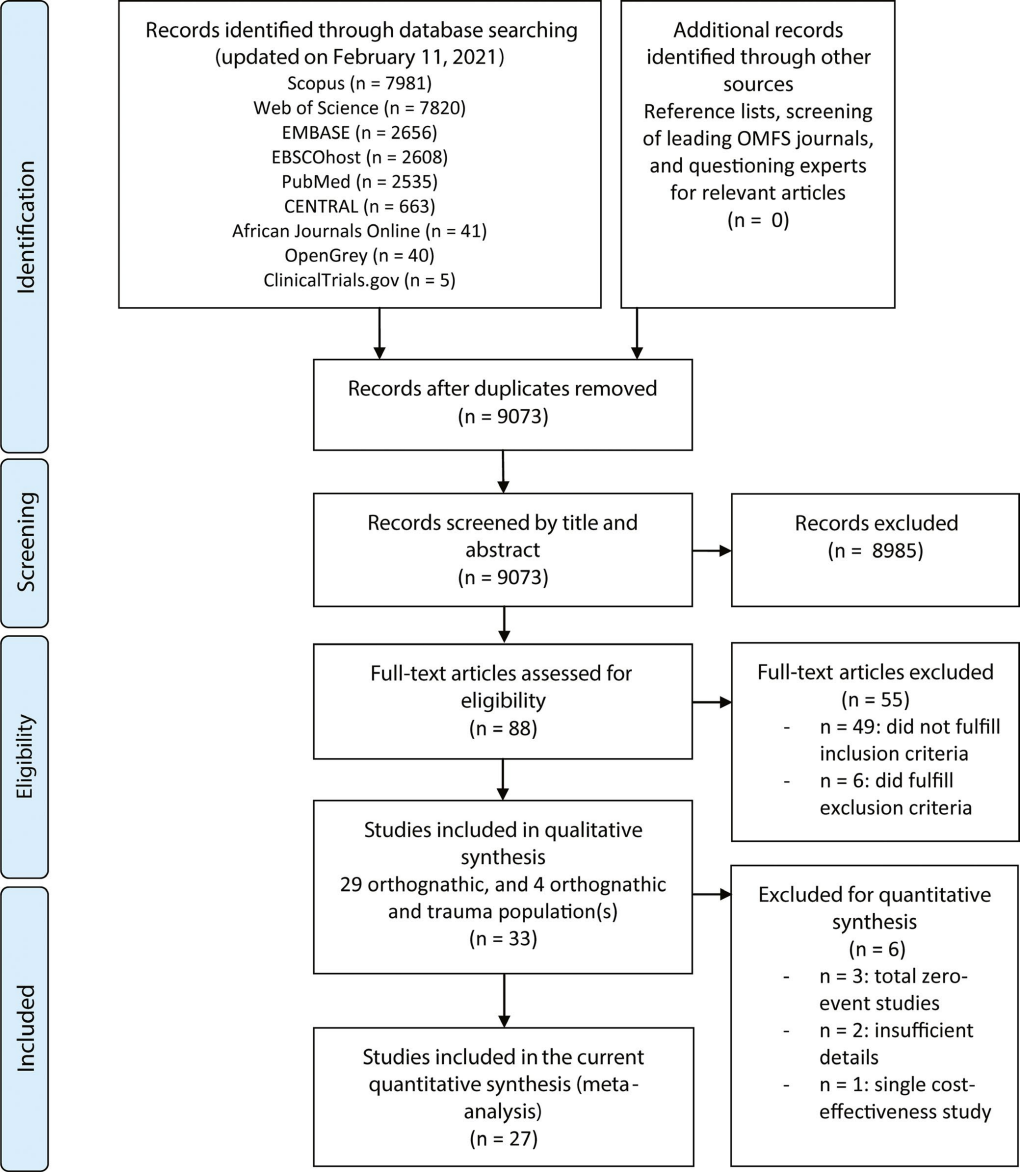
Flowchart of study identification and selection progress

The initial search was updated on February 11, 2021, and yielded 24,349 potentially eligible papers. A total of 9073 titles and abstracts was screened after eliminating duplicate records (Figure 1 and Table S4, kappa 0.91, agreement 99.7%). Eighty‐eight full‐text manuscripts were screened, and 33 and 27 articles were included in the qualitative and quantitative synthesis, respectively (agreement 100%, kappa 1.0). Of the screened full‐text manuscripts, two were written in the Korean [20, 37] and one in the Japanese language [38]. The third reviewer was not consulted.

### Patient characteristics

In total, 2551 patients (*N* = 33 studies), of which 1391 received titanium and 1160 received biodegradable osteosynthesis systems, were included (range 18–272; Table 1). The majority of patients were female. Ages ranged from 16 to 57 years. No study included only paediatric patients. Sixteen studies included patients with class III, [14, 18, 19, 20, 37, 39, 40, 41, 42, 43, 44, 45, 46, 47, 48, 49] six studies with class II, [15, 16, 50, 51, 52, 53] and 10 studies with both class II and class III malocclusion patients [2, 13, 54, 55, 56, 57, 58, 59, 60, 61]. One study did not specify the malocclusion of the included patients [17]. Five studies included both orthognathic and trauma patients [2, 52, 55, 56, 57]. Tobacco and alcohol usages by patients was reported in one study [17].

**TABLE 1:** Characteristics of the included studies

Study (first author, year)	Number of patients		Sex (M/F)		Age in years (mean ± SD or median (IQR))		Osteosynthesis system (outer screw diameter, mm)		Type of osteotomy (n)	Operative switches (B to T, n)	Orthodontic treatment	Duration of postoperative MMF	Follow‐up	Postoperative dietary restrictions
	*T*	*B*	*T*	*B*	*T*	*B*	*T*	*B*						
**Randomised controlled trials**														
Matthews et al. (2003) [50]	11	11	0/11	0/11	32 (range 18–46)	29 (range 21–44)	NM^m^	Biofix^a^, ^m^ (3.5)	BSSO advancement	0	Pre‐ and postoperative^q^	Soft guiding elastics; 4–5 weeks	1 year	
Norholt et al. (2004) [14]	30	30	10/20	12/18	22 (range 17–50)	23 (range 17–48)	W. Lorenz (2.0)	LactoSorb^b^ (2.0)	Le Fort I advancement ± impaction	0	Postoperative^r^	Soft guiding elastics; 2–4 weeks	2 and 6 weeks, 6 and 12 months	
Cheung et al. (2004) [17]	30	30	9/21	9/21	22.9 (range 16–37)		Mathys Compact 2.0 (2.0)	Biosorb FX^c^ (2.0 and 2.4)	Le Fort I maxillary subapical, mandibular subapical, mandibular body, sagittal split, genioplasty	0	Preoperative^s^		2 and 6 weeks, 3 and 6 months, 1 and 2 years	
Ueki et al. (2005) [19]	20	20					Würzburg (2.0)	Fixorb‐MX^d^ (2.0)	BSSO setback	0		Rigid, 2 weeks; soft guiding elastics^t^	1, 3, 6, and 12 months	
Cheung et al. (2008) [54]	20	20	17/23		24 ± 8.4	22 ± 5.5	Synthes	Inion CPS^e^ (2.0)	Le Fort I advancement/ setback/ impaction/ elongation	0	Preoperative^s^		2 weeks, and 3, 6, 12 months	Soft diet; 6 weeks
Park et al. (2010) [20]	10	30	4/6	17/13	22.8 ± 2.0	23.4 ± 2.9	M3 Visidisk	BioSorb FX^c^	Le Fort I advancement ± impaction and BSSO setback	0			6 mos	
Stockmann et al. (2010) [13]	33	33			27.0 ± 7.1	27.0 ± 5.4	Stryker^m^ (2.7)	Isosorb^f^, ^m^ (3.5)	BSSO advancement and setback	0	Postoperative^r^	Soft guiding elastics; 2–3 days	1, 2, 6 weeks, 3, 6 months, and 1, 2, 3, 4, 8 years	
Tuovinen et al. (2010) [15]	50	51	14/36	18/33	33.5		Stryker (max: 2.0; mand^m^: 2.0)	BioSorb FX^c^ (max: 2.4; mand^m^: 2.8)	Le Fort I advancement ± impaction and/or BSSO advancement	0	Postoperative^r^	Soft guiding elastics^t^	6.8 years (range 4.8–7.5)	
Buijs et al. (2012) [55]	124	76	47/77	34/42	30.5 ± 11.1	30.0 ± 11.9	KLS Martin (max: 1.5; mand: 2.0)	Inion CPS^e^ (max: 2.0; mand: 2.5)	Le Fort I, BSSO Le Fort I + BSSO	21	Postoperative^r^	Soft guiding elastics; 6–8 weeks	8 weeks	Soft diet; 5 weeks
Yoshioka et al. (2012) [18]	90	110	24/66	43/67	20 (range 18–37)	20 (range 18–45)	Stryker (2.0)	Neofix^g^ (2.2)	BSSO setback	0	Preoperative^s^	7 days; details NM	3, 6 months, and 1, 2, 3 years	
Bakelen et al. (2013) [56]	124	79	47/77	35/44	30.5 ± 11.1	30.0 ± 11.9	KLS Martin (max: 1.5; mand: 2.0)	Inion CPS^e^ (max: 2.0; mand: 2.5)	Le Fort I, BSSO Le Fort I + BSSO	21	Preoperative^s^	Soft guiding elastics; 6–8 weeks	1 and 2 years	Soft diet; 5 weeks
Yu et al. (2014) [16]	51	50	23/28	12/38	33.5 ± 14.3	31.2 ± 14.2	Stryker^m^	Inion CPS^e^, ^m^	BSSO advancement	0		T: Soft guiding elastics; 41.2% B: Soft guiding elastics; 96%	T: 8.06 ± 9.24 months B: 10.53 ± 7.33 months	
Bakelen et al. (2015) [57]	124	79	47/77	35/44	30.5 ± 11.1	30.0 ± 11.9	KLS Martin (max: 1.5; mand: 2.0)	Inion CPS^e^ (max: 2.0; mand: 2.5)	Le Fort I, BSSO, Le Fort I + BSSO	21	Preoperative^s^	Soft guiding elastics; 6–8 weeks	8 weeks and 2 years	Soft diet; 5 weeks
Gareb et al. (2017 z) [2]	124	79	47/77	35/44	30.5 ± 11.1	30.0 ± 11.9	KLS Martin (max: 1.5; mand: 2.0)	Inion CPS^e^ (max: 2.0; mand: 2.5)	Le Fort I, BSSO, Le Fort I + BSSO	21	Preoperative^s^	Soft guiding elastics; 6–8 weeks	T: 95 months (range 77–111) B: 98 months (range 80–11)	Soft diet; 5 weeks
**Prospective cohort studies**														
Ferrretti et al. (2002) [51]	20	20					NM ^m^ (2.0)	Lactosorb^b^, ^m^ (2.5)	BSSO advancement		Preoperative^t^ Postoperative 4 weeks	Soft guiding elastics^t^	1 and 6 weeks, and 3, 6, and 12 months	Pureed diet; 4 weeks
Dhol et al. (2008) [45]	25	25	8/17	5/20	22.9±1.6	23.3± 2.0		Lactosorb^b^ (2.0)	Le Fort I impaction				1 and 6 weeks, and 3, 6, and 12 months	
Bakelen et al. (2014) [52]	22	15	6/16	7/8	35 ± 11	35 ± 12	KLS Martin (2.0)	Inion CPS^e^ (2.5)	BSSO advancement		Pre‐ and postoperative^q^	Soft guiding elastics; 6–8 weeks	T: Mean 27 months B: Mean 25 months	Soft diet; 5 weeks
**Retrospective cohort studies**														
Harada et al. (1997) [46]	10	10	4/6	3/7	23.0 (range 18–30)	22.4 (range 20–31)	OSW Leibinger^m^ (2.7)	Takiron^h^, ^m^ (2.7)	BSSO setback		Pre‐ and postoperative^q^	T: Rigid; 9.4 days B: Rigid; 14.6 days	2 and 3 days, and 3, 6 and 12 months	
Costa et al. (2006) [47]	12	10			27.8 ± 5.9	26.9 ± 7.1		Lactosorb^b^ (2.0)	Le Fort I advancement ± impaction and BSSO setback			Rigid; 1 week	1 and 8 weeks, and 1 year	
Landes et al. (2006) [58]	30	30	23/37		25 (range 16–57)		Stryker (2.0)	MacroSorb^i^, PolyMax^j^ (2.0)	Le Fort I advancement/setback ± BSSO advancement/setback			Soft guiding elastics; 2–4 weeks	3 days and 1 year	Soft diet; 6 weeks
Turvey et al. (2006) [53]	35	34	16/18	11/24	26.8 ± 11.2	27.5 ± 13.0	NM^m^ (2.0)	BioSorb FX^c^, ^m^ (2.0)	BSSO advancement ± genioplasty		Pre‐ and postoperative^q^	Soft guiding elastics^t^	3, 5 weeks, and 1 year	
Ueki et al. (2006a, b) ^n^[48]	a: 12, b: 14	a: 12, b: 9	8/39		a: 21.8 (range 16–34), b: 26.5 (range 17–34)	a: 21.6 (range 17–32), b: 21.1 (range 19–25)	Würzburg (2.0)	Fixorb‐MX^d^ (2.0)	a: Le Fort advancement + BSSO setback, b: Le Fort I advancement + IVRO without fixation		Pre‐ and postoperative^q^	a: rigid, 'several days' + soft guiding elastics^t^ b: rigid, 1–3 weeks + soft guiding elastics^t^	1, 3, 6, 12 months	
Landes et al. (2007) [59]	30	15	23/22		27 (range 18–46)		Stryker (2.0)	LactoSorb^b^ , RapidSorb^k^ (2.0)	Le Fort I advancement/setback ± BSSO advancement/setback			Soft guiding elastics; 2–4 weeks	3 days and 1 year	Soft diet; 6 weeks
Ueki et al. (2009) [49]	12	11	3/20		25.1 ± 7.3		Stryker (2.0)	Fixorb‐MX^d^ (2.0)	BSSO setback		Pre‐ and postoperative^q^	Rigid, 1 week; soft guiding elastics^t^	1 yr	
Ahn et al. (2010) [60]	152	120	126/146		23			BioSorb FX^c^ (max: 2.0; mand: 2.4); (genioplasty: 2.0)	BSSO ± Le Fort I ± genioplasty				Follow‐up examinations on a regular basis	
Choi et al. (2010) [37]	15	15	9/6	7/8	21.8 (range 17–32)	21 (range 17–31)	Stryker (2.0)	Inion CPS^e^ (2.0)	BSSO setback		Pre‐ and postoperative^q^	Rigid 3 days	14.5 months	
Ueki et al. (2011) [39]	20	20^1^; 20^4^	10/10	5/15^12^ 10/10^4^	21.7 ± 5.6	29.1 ± 11.2^12^ 23.5±5.9^4^	Würzburg (2.0)	Super‐Fixsorb‐MX^l^ , Fixorb‐MX^d^ (2.0)	BSSO setback			Rigid, 'few days' + soft guiding elastics^t^	1, 3, 12 months	
Ballon et al. (2012) [61]	43	41	22/21	20/21	25 (range 16–57)	24 (range 16–46)	Stryker‐Leibinger (2.0)	Inion CPS^e^ (max : 2.0 ; mand : 2.5)	Le Fort I ± BSSO BSSO^u^			Soft guiding elastics; 2–6 weeks	T: 35 (6–113) months B: 13 (7–27) months	Soft diet; 6 weeks
Paeng et al. (2012) [40]	25	25	13/12	11/14	25.3 ± 4.1	22.6 ± 2.9	Le Forte system^m^ (2.4)	Inion CPS^e^, ^m^ (2.5)	BSSO setback			Soft guiding elastics^t^	3 days, and 2, 6, 12 months	
Ueki et al. (2012) [41]	20	20^1^; 20^4^	4/16	9/11^12^ 4/16^4^	21.6 ± 4.4	26.4 ± 8.6^12^ 23.8± 6.4^4^	Würzburg (2.0)	Super‐Fixsorb‐MX^l^, Fixorb‐MX^d^ (2.0)	Le Fort I advancement ± impaction + BSSO setback		Pre‐ and postoperative^q^	Rigid, 'few days' + soft guiding elastics^t^	1, 3, 12 months	
Blakey et al. (2014) [42]	30	27	14/16	7/20	20.8 ± 6.4	19.7 ± 5.5	Leibinger, Stryker (2.0)	Inion CPS^e^ or BioSorb FX^c^ (2.0)	Le Fort I advancement		Pre‐ and postoperative^q^	Soft guiding elastics; 6 weeks	1 year	
Lee et al. (2014) ^o^[43]	10	8					Le Forte system	BioSorb FX^c^	BSSO setback		B: Preoperative 2.75 ± 1.82 months; post‐operative^t^ T: Preoperative 2.34 ± 1.89 months; post‐operative^t^	Rigid; 2–3 weeks	6 months	
Ueki et al. (2015) ^p^[44]	13	35					Stryker (2.0)	Fixorb‐MX^d^ (2.0)	BSSO setback ± Le Fort I advancement		Pre‐ and postoperative^q^	Soft guiding elastics^t^	1 year	

Osteosyntheses were performed using plates and screws, unless stated otherwise.

Abbreviations: B, biodegradable osteosynthesis; BSSO, bilateral sagittal split osteotomy; F, female; IVRO, intraoral vertical ramus osteotomy; M, male; mand, mandible; max, maxilla; MMF, maxillomandibular fixation; mos, months; NM, not mentioned; PDLA, poly‐D‐lactic acid; PDLLA, poly‐D,L‐lactic acid; PGA, polyglycolic acid; PLLA, poly‐L‐lactic acid; T, titanium osteosynthesis; T, titanium osteosynthesis; TMC, trimethylene carbonate; uHA, unsintered hydroxyapatite. Empty cells: not reported.

^a^ Biofix (self‐reinforced PLLA).

^b^ Lactosorb (82/18 PLLA/PGA).

^c^ BioSorb FX (self‐reinforced 70/30 PLLA/PDLLA).

^d^ Fixorb‐MX (100 PLLA).

^e^ Inion CPS (79/15/6 PDLLA/PDLA/TMC).

^f^ Isosorb (80/20 (90/10 PLLA/PDLLA) /(50/50 PLLA/PDLA)).

^g^ Neofix (100 PLLA).

^h^ Takiron (100 PLLA).

^i^ MacroSorb (70/30 PLLA/PDLLA).

^j^ PolyMax (70/30 PLLA/PDLLA).

^k^ RapidSorb (85/15 PLLA/PGA).

^l^ Super‐Fixsorb‐MX (40/60 uHA/PLLA).

^m^ Osteosyntheses performed with screws only.

^n^ Identical study, but different comparisons: (a) Le Fort I + BSSO or (b) Le Fort I + IVRO without osteosyntheses.

^o^ Only subgroup 2 and 3 of the original manuscript are relevant for the present review. The distribution of sex and age is not given for each subgroup.

^p^ Only subgroup 1–3 of the original manuscript are relevant for the present review. The distribution of sex and age is not given for each subgroup

^q^ Durations not mentioned

^r^ Preoperative and durations not mentioned

^s^ Postoperative and durations not mentioned

^t^ Duration not mentioned

^u^ Only used with titanium osteosynthesis

### Procedural characteristics

The characteristics of all included procedures are presented in Table 1. The titanium screw diameters varied from 1.5 to 2.0 mm whenever osteosynthesis plates were used, while the screw diameters varied from 2.0 to 2.7 mm whenever osteosyntheses was performed solely with screws.

BioSorb FX (self‐reinforced 70/30 PLLA/PDLLA) and Inion CPS (79/15/6 poly‐L‐lactic acid (PLLA)/poly‐DL‐lactic acid (PDLLA)/trimethylene carbonate) were the biodegradable osteosynthesis systems that were most frequently used (Table 1). The biodegradable screw diameters varied from 2.0 to 3.5 mm. Perioperative switching from biodegradable to titanium osteosyntheses was reported in four articles [2, 55, 56, 57]. Main reasons for switching was non‐grips screws resulting in inadequate fixation or a lack of stability of the fixated bone segments [62].

The most commonly performed surgical procedures were Le Fort I and bilateral sagittal split osteotomies. Only one study reported duration of the pre‐ and postoperative orthodontic treatment [43] while one study only reported duration of postoperative orthodontic treatment [51]. Postoperative maxillomandibular fixation duration ranged from 1 week to 8 weeks and most of the studies used soft guiding elastics for maxillomandibular fixation. Four studies only used rigid maxillomandibular fixation, [37, 43, 46, 47] while five studies used a combination of rigid and soft guiding elastics for maxillomandibular fixation [19, 39, 41, 48, 49]. Ten studies reported on postoperative dietary restrictions, a soft or pureed diet for 4–6 weeks [2, 51, 52, 54, 55, 56, 57, 58, 59, 61].

### Risk of bias assessment

Of all included articles, 14 were publications of RCTs, [2, 13, 14, 15, 16, 17, 18, 19, 20, 50, 54, 55, 56, 57] and 4 of these publications were from the same RCT with different follow‐up times [2, 55, 56, 57]; three were prospective cohort studies [45, 51, 52]; and 16 were retrospective cohort studies [37, 39, 40, 41, 42, 43, 44, 46, 47, 48, 49, 53, 58, 59, 60, 61]. Seven publications of RCTs were assessed having ‘some concerns’ regarding risk of bias (Table 2). All other RCTs had high risk of bias (Table 2). Of all included cohort studies, none were assessed as having an unbiased assessment of study endpoints while 42% of the included studies had adequate contemporary groups (i.e. biodegradable and titanium groups; Table 3).

**TABLE 2:** Risk of bias assessment of the included randomized controlled trials

*Study name (year)*	Revised Cochrane risk‐of‐bias tool for randomized trials (RoB 2)					
	Domain 1	Domain 2	Domain 3	Domain 4	Domain 5	Overall risk‐of‐bias
Matthews et al. (2003) [50]	SC	L	L	L	L	SC
Norholt et al. (2004) [14]	SC	L	L	L	L	SC
Cheung et al. (2004) [17]	SC	L	SC	SC	L	SC
Ueki et al. (2005) [19]	SC	L	L	L	L	SC
Cheung et al. (2008) [54]	SC	L	H	L	L	H
Park et al. (2010) [20]	SC	L	L	L	SC	SC
Stockmann et al. (2010) [13]	SC	L	L	L	L	SC
Tuovinen et al. (2010) [15]	SC	L	L	L	L	SC
Buijs et al. (2012) [55]	L	H	L	L	L	H
Yoshioka et al. (2012) [18]	SC	L	H	H	L	H
Bakelen et al. (2013) [56]	L	H	L	L	L	H
Yu et al. (2014) [16]	H	L	SC	L	SC	H
Bakelen et al. (2015) [57]	L	H	L	L	L	H
Gareb et al. (2017) [2]	L	H	L	L	L	H

Domain 1, Bias arising from the randomization process; Domain 2, Bias due to deviations from the intended intervention; Domain 3, Bias due to missing outcome data; Domain 4, Bias in measurement of outcome; Domain 5, Bias in selection of reported results; H, high risk of bias; L, low risk of bias; SC, some concerns.

**TABLE 3:** Risk of bias assessment of the included cohort studies

Study name (year)	MINORS											
	Clearly stated aim	Inclusion of consecutive patients	Prospective data collection	Endpoints appropriate to aim of study	Unbiased assessment of study endpoint	Follow‐up period appropriate to aim of study	Loss to follow‐up <5%	Prospective calculation of study size	Adequate control group	Contemporary groups	Baseline equivalence of groups	Adequate statistical analyses
Prospective cohort studies												
Ferrretti et al. (2002 ) [51]	2	2	2	2	0	1	2	0	2	2	2	1
Dhol et al. (2008) [46]	2	2	2	2	0	1	2	0	2	2	2	2
Bakelen et al. (2014) [52]	2	2	2	2	1	2	0	2	2	2	2	2
Study name (year)												
Harada et al. (1997) [46]	2	0	0	2	0	2	2	0	2	0	2	0
Costa et al. (2006) [47]	2	2	0	2	0	2	2	0	2	2	2	2
Landes et al. (2006) [58]	2	1	0	2	1	2	2	0	2	2	0	2
Turvey et al. (2006) [53]	2	1	0	2	0	2	2	0	2	1	1	0
Ueki et al. (2006) [48]	2	0	0	2	0	2	0	0	2	0	1	2
Landes et al. (2007) [59]	2	1	0	2	1	2	0	0	2	1	0	2
Ueki et al. (2009) [49]	2	0	0	2	0	2	0	0	2	0	1	2
Ahn et al. (2010) [60]	2	1	0	1	0	0	0	0	2	2	0	1
Choi et al. (2010) [37]	2	1	0	2	0	2	0	0	2	1	2	2
Ueki et al. (2011) [39]	2	0	0	2	0	2	0	0	2	0	1	2
Ballon et al. (2012) [61]	2	0	0	2	0	2	0	0	2	1	1	2
Paeng et al. (2012) [40]	2	2	0	2	0	1	2	0	2	0	2	1
Ueki et al. (2012) [41]	2	0	0	2	0	2	2	1	2	0	2	2
Blakey et al. (2014) [42]	2	0	0	2	0	2	0	0	2	2	1	2
Lee et al. (2014) [43]	2	0	0	2	1	1	2	0	2	2	1	2
Ueki et al. (2015) [44]	2	0	0	2	0	2	2	0	2	1	1	2

MINORS, Methodological index for non‐randomized studies. 0: not reported; 1: reported but inadequate; 2: reported and adequate.

Two studies reported funding from research programmes [15, 42] and one from the manufacturer of biodegradable osteosyntheses [13]. Funding or conflict of interest was not reported in 20 studies [14, 16, 17, 18, 19, 20, 37, 39, 43, 44, 45, 46, 47, 48, 49, 50, 51, 53, 59, 60]. The other studies declared no funding or conflict of interest [2, 40, 41, 52, 54, 55, 56, 57, 58, 61].

### Primary endpoint

All effect estimates of pooled endpoints are presented as RR or SMD (95% CI) including the quality of evidence. Five studies reported on malunion (Table S5) [19, 44, 47, 49, 55]. In one RCT malunion was found present at 8 weeks of follow‐up in 3% of biodegradable osteosyntheses and 0% of titanium osteosyntheses [55]. The other four studies assessing this endpoint were total zero‐event studies and, thus, pooling of data was not possible.

### Secondary endpoints

**FIGURE 2:**
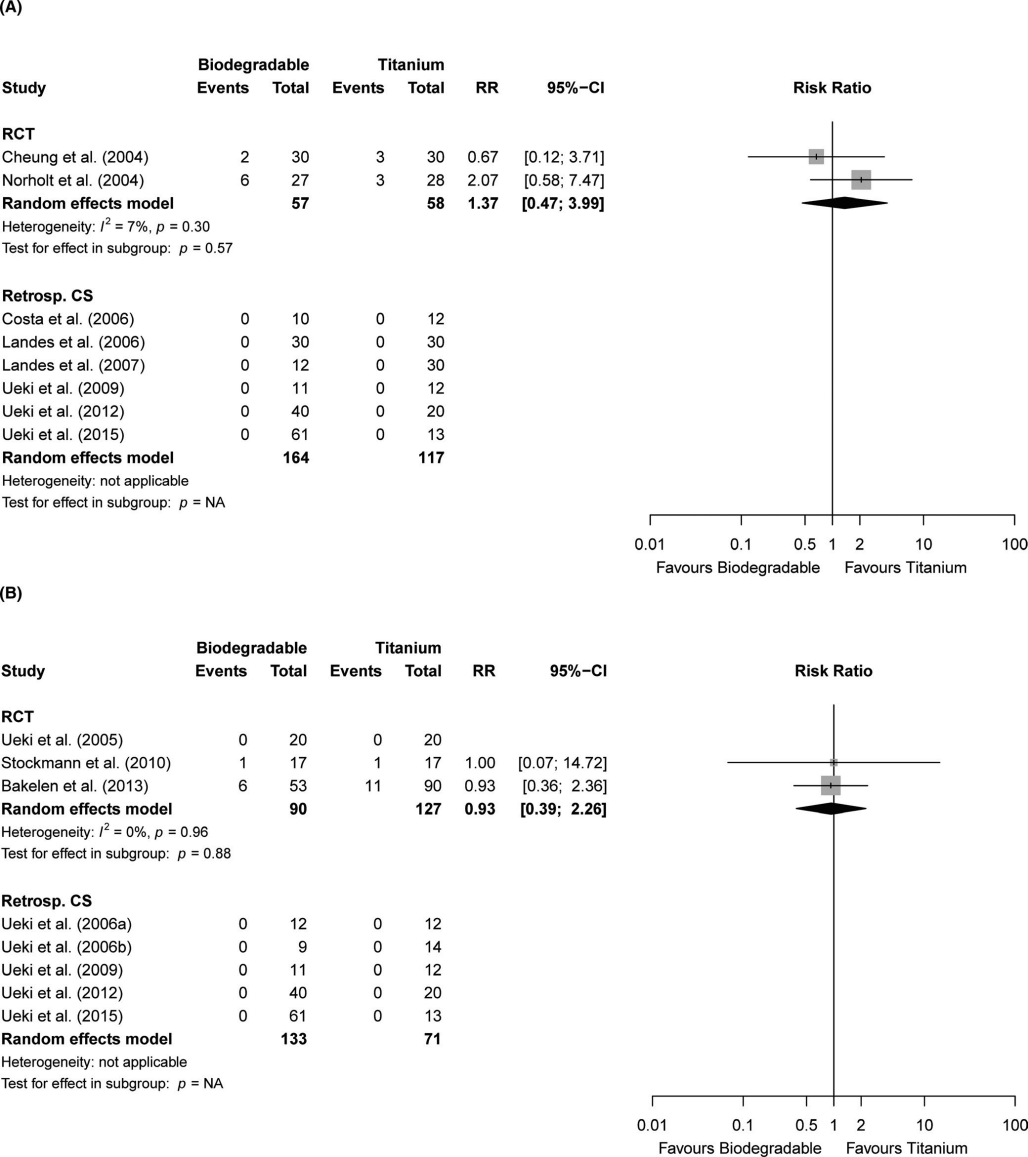
Forest plots of the endpoints (A) mobility of bone segments (6–12 weeks follow‐up) and (B) malocclusion (>12 weeks follow‐up) stratified by study design. 95%‐CI, 95% confidence interval; NA, not applicable; RCT, randomised controlled trials; RR, risk ratio

Mobility of bone segments at 6–12 weeks follow‐up was evaluated in 10 studies (Table S5) [14, 17, 19, 41, 44, 47, 49, 54, 58, 59]. Mobility of bone segments was assessed as not present in seven studies [19, 41, 44, 47, 49, 58, 59]. No significant difference was found between the biodegradable and titanium groups [RCTs: RR 1.37 (0.47; 3.99), *p* = 0.57, n = 2 studies, moderate quality, Figure 2A].

Malocclusion within 4 weeks of follow‐up was not reported in any of the included studies (Table S5). One RCT reported that 9% and 13% of the patients had objective malocclusion at 6–12 weeks of follow‐up in the biodegradable and titanium group, respectively (Table S5).[55] Malocclusion after >12 weeks follow‐up ranged from 0% to 15% (n = 8 studies). All retrospective studies reporting this endpoint found no malocclusion in either treatment groups [41, 44, 48, 49]. Pooling data from RCTs that assessed objective malocclusion did not result in significant differences between both groups [RR 0.93 (0.39; 2.26), *p* = 0.88, n = 3 studies, moderate quality, Figure 2B]. One RCT with >5 years of follow‐up reported that 12% and 15% of patients in the biodegradable and titanium groups, respectively, had subjective malocclusion [2]. No subgroup analyses of objective malocclusion at short‐term follow‐up (no studies), intermediate follow‐up (single study), and long‐term follow‐up (single studies in each of the subgroups osteosyntheses with plates/screws vs. only screws) could be performed. Additionally, subgroup analyses of subjective malocclusion at any of the pre‐specified follow‐up moments could not be performed (single study).

Perioperative plate breakage ranged from 0 to 3% and 0% among patients in the biodegradable and titanium groups, respectively (n = 4 studies, Table S5). One RCT assessed plate breakage at plate‐level and reported 4% plate breakage in the biodegradable osteosyntheses group and 0% titanium plates [17]. Regarding screws, 0%–12% biodegradable and 3% of the titanium screws broke (n = 7 studies) [13]. Data of screw breakage in retrospective studies could not be pooled because two studies [58, 59] reported the percentage of broken screws without giving the total number of included screws and these numbers could not be provided by the corresponding authors. The operative time in the RCTs was significantly longer in the biodegradable compared to the titanium group [SMD 0.50 (0.09; 0.91], *p* = 0.02, n = 2 studies, moderate quality, Figure S2). Plate and screw handling was easier in the titanium compared to the biodegradable group but could not be included in the quantitative analysis (Table S5) [14, 55].

**FIGURE 3:**
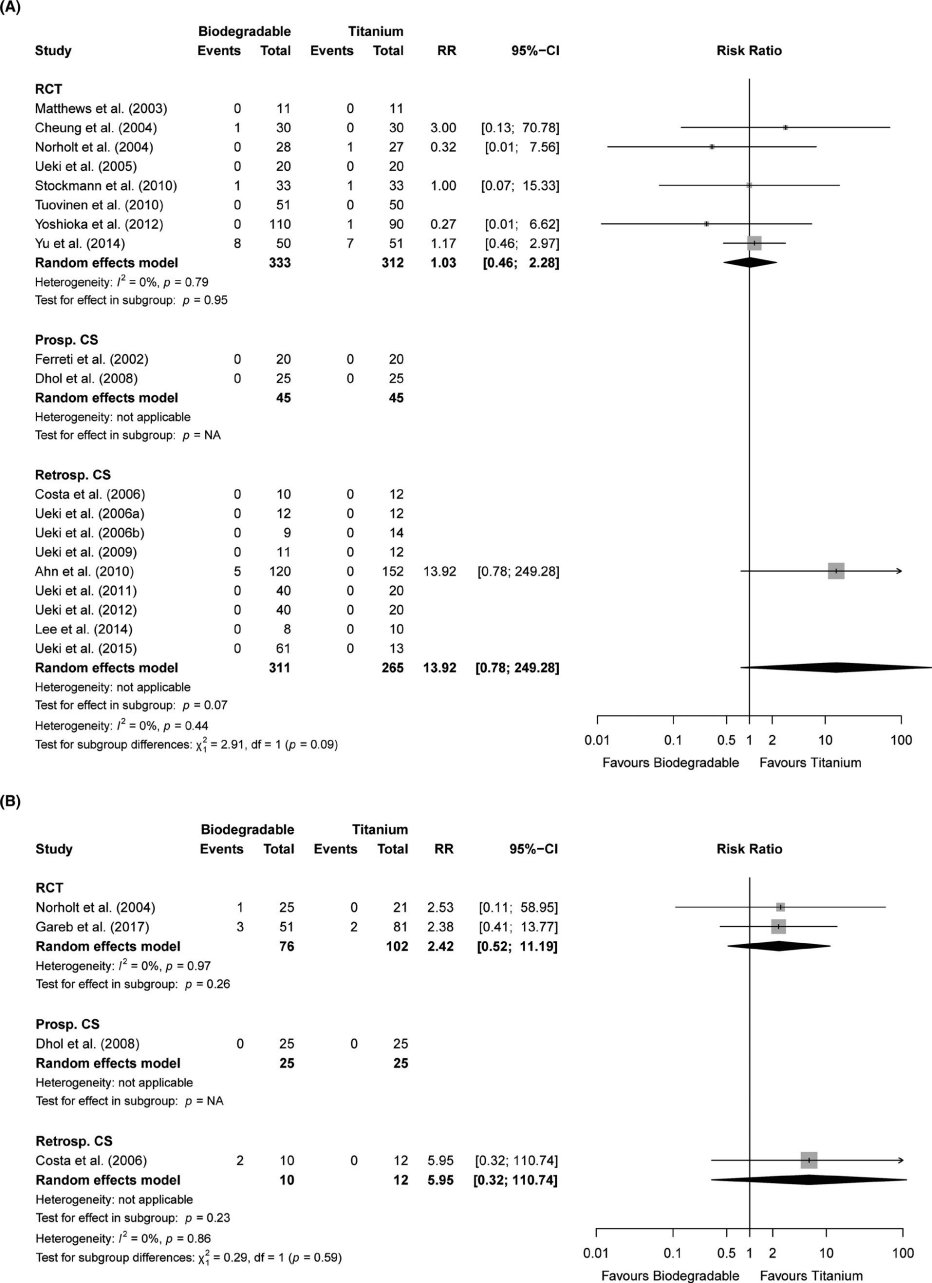
Forest plots of the endpoints (A) infection (<4 weeks follow‐up) and (B) swelling (>12 weeks follow‐up) stratified by study design. 95%‐CI, 95% confidence interval; Prosp. CS, prospective cohort studies; RCT, randomised controlled trials; Retrosp. CS, retrospective cohort studies; RR, risk ratio

Infection within 4 weeks of follow‐up was reported in 0%–16% and 0%–14% in the biodegradable and titanium groups (n = 18 studies, Table S5), respectively, and did not differ significantly between both groups [RR 1.03 (0.46; 2.28), *p* = 0.95, n = 8 studies, moderate quality, Figure 3A]. Swelling within 4 weeks of follow‐up did not differ significantly between both treatment groups [RR 1.51 (0.68; 3.38), *p* = 0.31, n = 2 studies, very low quality, Figure S3] [14, 55]. Abscess formation was present in 12% and 5% of the patients (n = 1 study) treated with biodegradable and titanium osteosyntheses, respectively [55]. Pain within short‐term follow‐up varied from 0%–25% in the biodegradable and 0%–30% in the titanium group (n = 3 studies). Maximal mouth opening within 4 weeks of follow‐up was reported in one study but that study did not present exact numbers [13]. Dehiscence varied between 0%–7% and 0%–10% in the biodegradable and titanium groups, respectively [RCTs: RR 1.53 (0.52; 4.50), *p* = 0.44, n = 5 studies, moderate quality; Figure S4 and Table S5]. Plate exposure after short‐term follow‐up was reported in 0%–9% and 0% in the biodegradable and titanium groups, respectively (n = 8 studies).

Pain within 6–12 weeks of follow‐up was not significantly different between both treatment groups [SMD −0.1 (−0.26; 0.24), *p* = 0.93, n = 2 studies, moderate quality, Figure S5 and Table S5]. Two studies assessed the presence of TMJ‐dysfunction after intermediate follow‐up [46, 50]. None of these included patients were diagnosed with having TMJ‐dysfunction.

**FIGURE 4:**
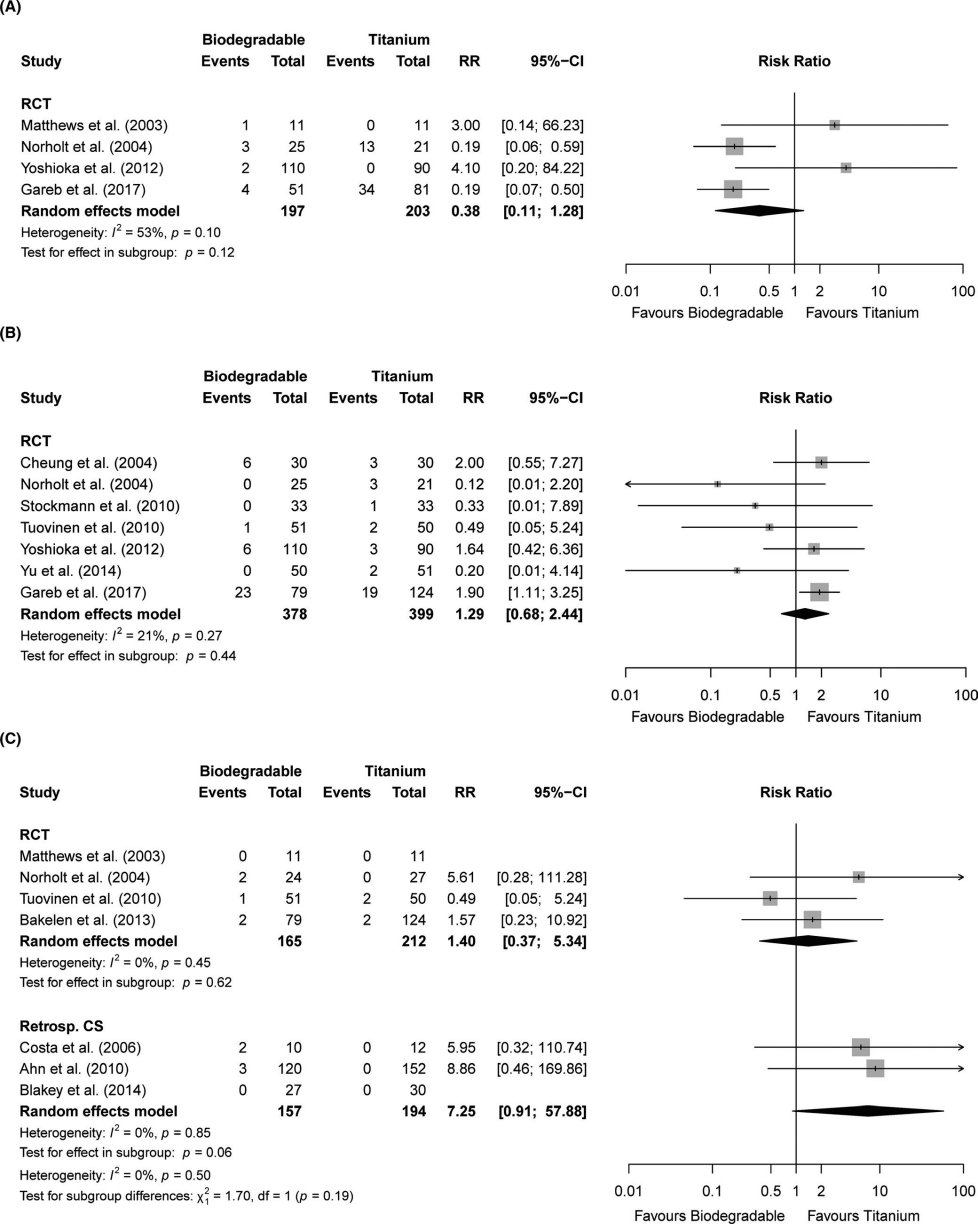
Forest plots of the endpoints (A) palpability of plates/screws (>12 weeks follow‐up), (B) symptomatic device removal (overall follow‐up), and (C) revision surgery (overall follow‐up) stratified by study design. 95%‐CI, 95% confidence interval; RCT, randomised controlled trials; Retrosp. CS, retrospective cohort studies; RR, risk ratio

Regarding long‐term follow‐up, the amount of pain was generally low in both treatment groups [RCTs: SMD −0.02 (−0.29; 0.25), *p* = 0.89, n = 3 studies, high quality, Figure S6 and Table S5]. Maximal mouth opening (at >12 weeks of follow‐up) was not significantly different between both groups [RCTs: SMD −0.58 (−1.39; 0.22), n = 2 studies, *p* = 0.16, very low quality, Figure S7]. The presence of TMJ‐dysfunction ranged from 0%–15% in the biodegradable and 0%–25% in the titanium group (n = 3 studies). MFIQ scores were similar after >5 years of follow‐up for both treatment groups [median 18 (interquartile range 17–21)] [2]. A single RCT reported a similar percentage (3%) of abscesses at 1‐year follow‐up [56]. RCTs showed no significant differences between both groups regarding long‐term swelling [RR 2.42 (0.52; 11.19), *p* = 0.26, n = 2 studies, moderate quality; Figure 3B]. Plate and screw palpability after long‐term follow‐up occurred in 2%–51% and 0%–42% of the biodegradable and titanium groups, respectively [RCTs: RR 0.38 (0.11; 1.28), *p* = 0.12, n = 4 studies, very low quality, Figure 4A]. Patients of both groups were comparable regarding satisfaction with the result after 2, [17] 5, [2] and 8 years of follow‐up [13] (n = 3 studies, Table S5).

### Secondary surgery and total costs

Symptomatic biodegradable and titanium device removal frequencies varied from 0%–29% and 0%–15%, respectively [RCTs: RR 1.29 (0.68; 2.44), *p* = 0.44, n = 7 studies, moderate quality, Figure 4B and Table S5]. The follow‐up time varied from 8 weeks to 8 years (Table 1). Chronic infection and discomfort were the main reasons for symptomatic device removal. No differences were found between the maxillary vs. mandibular vs. bimaxillary osteotomies [maxillary: RR 0.12 (0.01; 2.20), *p* = 0.15, n = 1 study; mandibular: RR 1.60 (0.76; 3.34), *p* = 0.21, n = 4 studies; bimaxillary: RR 1.45 (0.64; 3.27), *p* = 0.37; *p* = 0.09, Figure S8]. A subgroup analysis of osteosyntheses using only screws vs. plates and screws revealed no significant difference in symptomatic device removal rate between both subgroups [screws: RR 0.26 (0.03; 2.28), *p* = 0.22, n = 2 studies; plates and screws: RR 1.86 (1.13; 3.07), *p* = 0.01, n = 2 studies; *p* = 0.08; Figure S9]. A subgroup analysis of the symptomatic device removal rates at ≤1 year and >1 year of follow‐up resulted in similar symptomatic device removal rates of biodegradable and titanium osteosyntheses at ≤1 year of follow‐up [RR 0.16 (0.02; 1.26), *p* = 0.08, n = 2 studies], while titanium osteosyntheses had lower symptomatic device removal rates if only studies with >1‐year follow‐up time were included [RR 1.73 (1.10; 2.72), *p* = 0.02, n = 5 studies; Figure S10].

Total costs (i.e., indirect and direct costs) were assessed in one RCT with 2 years of follow‐up [57]. The mean costs of the biodegradable and titanium groups were €6589 ± 3492 and €6787 ± 5014, respectively. Revision surgery (i.e. device removal not included) ranged from 0%–8% and 0%–4% of the patients in the biodegradable group and titanium group, respectively [RCTs: RR 1.40 (0.37;5.34), *p* *= *0.62, n = 4 studies, moderate quality, Figure 4C]. Chronic infection and abscess formation were the main reasons to indicate revision surgery.

### Skeletal stability

The amount of operative displacement, amount of relapse with the corresponding follow‐up, and the lateral reference marks used by all studies that assessed skeletal stability are presented in Table S6. Follow‐up ranged from 6 weeks to 2 years. The majority of studies assessed skeletal stability after 1‐year follow‐up.

Horizontal relapse after a Le Fort I advancement was assessed in three RCTs, [14, 20, 54] one prospective study, [45] and seven retrospective studies [41, 42, 47, 48, 58, 59, 61]. RCT data could not be pooled because one study did not provide exact numbers [54] and one study reported zero variance in the amount of relapse. The retrospective studies’ data showed no significant difference in the amount of relapse between biodegradable and titanium osteosyntheses [SMD 0.15 (−0.08; 0.39), *p* = 0.21, n = 7 studies, very low quality, Figure S11]. Angular relapse after maxillary advancement did not differ significantly between both treatment groups [SMD 0.07 (−0.41; 0.55), *p* = 0.78, n = 4 studies, very low quality, Figure S12]. No significant difference in horizontal relapse after a Le Fort I setback between biodegradable and titanium osteosyntheses groups was found [SMD −0.02 (−0.61; 0.57), *p* = 0.95, n = 3 studies, very low quality, Figure S13].

Vertical relapse after maxillary impaction did not differ significantly between both treatment groups [SMD 0.07 (−0.35; 0.50), *p* = 0.74, n = 2 studies, high quality, Figure S14]. The amount of maxillary relapse after maxillary elongation was not significantly different between both types of osteosynthesis systems [SMD 0.31 (−0.23; 0.84), *p* = 0.26, n = 2 studies, very low quality, Figure S15].

Horizontal relapse after mandibular advancement was not significantly different between both treatment groups [SMD 0.16 (−0.39; 0.71), *p* = 0.56, n = 2 studies, very low quality, Figure S16]. Two RCTs [19, 20] and seven retrospective studies [37, 40, 43, 46, 58, 59, 61] assessed horizontal relapse after mandibular setback. Pooling of data showed no significant differences between biodegradable and titanium osteosyntheses [RCTs: SMD 0.04 (−0.73; 0.80), *p* = 0.92, n = 2 studies, low quality, Figure S17]. A subgroup comparison of horizontal relapse after mandibular setback between osteosyntheses with plates and screws vs. only screws resulted in no significant difference between subgroups (n = 5 studies, *p* = 0.99; Figure S18).

Data regarding vertical relapse after mandibular setback showed a significant difference in favour of biodegradable osteosyntheses opposed to titanium osteosyntheses [RCTs: SMD −0.63 (−1.11; −0.15), *p* = 0.01, n = 2 studies, low quality, Figure S19]. There was no significant difference in the subgroup analysis of osteosyntheses plates and screws vs. only screws (n = 5 studies, *p* = 0.58; Figure S20).

A quantitative analysis of the data of mandibular angular relapse after clockwise rotation (CW) showed significantly less relapse in the biodegradable osteosyntheses group [SMD −0.79 (−1.40; −0.17), *p* = 0.01, n = 4 studies, very low quality, Figure S21]. Regarding mandibular angular relapse after counter‐clockwise rotation, RCTs’ data showed significantly less relapse in the titanium osteosyntheses group [SMD 1.12 (0.08; 2.16), *p* = 0.03, n = 2 studies, very low quality, Figure S22]. All assessed endpoints with the quality of evidence are summarized in Tables 4 and 5.

**TABLE 4:** Summary of findings of the randomized controlled trials with quality of evidence assessment

Randomized controlled trials					
Outcome	Subjects, N (studies)	RR or SMD (95% CI)	Tit. event proportion	Bio. risk (95% CI)	Quality of evidence (GRADE)
Perioperative endpoints					
Plate breakage^a^	482 (4)	Four studies, of which two had zero events, one assessed plate breakage at plate level, and one at patient level			
Screw breakage^a^	348 (4)	Four studies, of which two had zero events, one assessed screw breakage at screw level, and one at patient level			
Operation time^b^	266 (2)	+0.50 (0.09; 0.91)	NA	NA	Moderate^c^
Handling by surgeon ^b^	260 (2)	Two studies, different outcome measures			
Short‐term follow‐up					
Malocclusion^a^	No studies				
Infection^a^	645 (8)	1.03 (0.46; 2.28)	43 per 1000	45 per 1000 (20; 98)	Moderate^f^
Swelling^a^	255 (2)	1.51 (0.68; 3.38)	133 per 1000	201 per 1000 (91; 450)	Very low^c^, ^d^, ^f^
Abscess^a^	200 (1)	Single study			
Pain^a^	160 (3)	Three studies, two with different outcome measures and one provided data in graphs only			
Analgesics used^a^	No studies				
MMO^b^	66 (1)	Single study that provided data in graphs only			
Dehiscence^a^	421 (5)	1.53 (0.52; 4.50)	24 per 1000	37 per 1000 (13; 108)	Moderate^f^
Plate exposure^a^	182 (4)	Four studies, of which two had zero events and one did not provide sufficient details			
Intermediate follow‐up					
Malunion^a^	240 (2)	Two studies, of which one had zero events			
Mobility bone segments^a^	115 (2)	1.37 (0.47; 3.99)	104 per 1000	143 per 1000 (49; 415)	Moderate^f^
Malocclusion^a^	200 (1)	Single study			
Pain^b^	260 (2)	−0.01 (−0.26; 0.24)	NA	NA	Moderate^c^
MMO^b^	66 (1)	Single study that provided data in graphs only			
TMJ‐dysfunction^a^	22 (1)	Single zero‐event study			
Long‐term follow‐up					
Malocclusion^a^	217 (3)	0.93 (0.39; 2.26)	113 per 1000	105 per 1000 (44; 256)	Moderate^f^
Pain^b^	220 (3)	−0.02 (−0.29; 0.25)	NA	NA	High
MMO^b^	141 (2)	−0.58 (−1.39; 0.22)	NA	NA	Very low^c^, ^e^, ^f^
TMJ‐dysfunction^a^	40 (1)	Single study			
MFIQ^b^	203 (1)	Single study			
Abscess^a^	203 (1)	Single study			
Swelling^a^	178 (2)	2.42 (0.52; 11.19)	20 per 1000	49 per 1000 (11; 224)	Moderate^c^, ^f^, ^g^
Palpability plate/screws^a^	400 (4)	0.38 (0.11; 1.28)	232 per 1000	89 per 1000 (26; 297)	Very low^c^, ^d^, ^f^
Satisfaction^b^	329 (3)	Three studies, different outcome measures			
Overall follow‐up					
Symptomatic device removal^a^	777 (7)	1.29 (0.68; 2.44)	83 per 1000	107 per 1000 (57; 203)	Moderate^4^
Total costs^b^	203 (1)	Single study			
Revision surgery (not device removal)^a^	377 (4)	1.40 (0.37; 5.34)	20 per 1000	28 per 1000 (8; 107)	Moderate^4^
Maxillary horizontal relapse (adv)	160 (3)	Three studies, of which one provided data in graphs only and one could not be included in the meta‐analysis due to zero variance in the titanium group			
Maxillary angular relapse (adv)	100 (2)	Two studies, significant different reference points used for assessment			
Maxillary horizontal relapse (sb)	No studies				
Maxillary angle relapse (sb)	No studies				
Maxillary vertical relapse (imp)	95 (2)	+0.07 (−0.35; 0.50)	NA	NA	High
Maxillary vertical relapse (elong)	60 (1)	Single study			
Mandibular horizontal relapse (adv)	80 (2)	Two studies, of which one provided data in median with interquartile range and one provided insufficient details			
Mandibular horizontal relapse (sb)	80 (2)	+0.04 (−0.73; 0.80)	NA	NA	Low^d^, ^f^
Mandibular vertical relapse (adv)	80 (2)	Two studies, of which one provided data in median with interquartile range and one provided insufficient details			
Mandibular vertical relapse (sb)	80 (2)	−0.63 (−1.11; −0.15)	NA	NA	Low^d^, ^f^
Mandibular angular relapse (CW)	22 (1)	Single study			
Mandibular angular relapse (CCW)	80 (2)	1.12 (0.08; 2.16)	NA	NA	Very low^d^, ^e^, ^f^

Abbreviations: adv, advancement; Bio, biodegradable osteosynthesis; CCW, counter clockwise rotation; CW, clockwise rotation; elong, elongation; GRADE, Grades of Recommendation, Assessment, Development and Evaluation Working Group system; NA, not applicable; RR, risk ratio (binary variables); sb, setback; imp, impaction; SMD, standardized mean difference (continuous variables); Tit, titanium osteosynthesis.

^a^ Binary variable.

^b^ Continuous variable.

^c^ Downgraded one level due to high risks of bias identified across studies: majority of studies had high risk of bias.

^d^ Downgraded one level for inconsistency: substantial methodological or clinical heterogeneity that could not be accounted for in analyses.

^e^ Downgraded one level for indirectness: the evidence of the original manuscripts were more restrictive than the review question.

^f^ Downgraded one level for imprecision: limits of effect estimate confidence interval are not consistent (i.e., cover both benefit and harm).

^g^ Upgraded one level due to large effect (i.e., RR < 0.5 or RR > 2.0, or SMD < −0.8 or SMD > +0.8).

**TABLE 5:** Summary of findings of the included cohort studies with quality of evidence assessment

Outcome	Prospective cohort studies					Retrospective cohort studies				
	Subjects, N (studies)	RR or SMD (95% CI)	Tit. Event proportion	Bio. Risk (95% CI)	Quality of evidence (GRADE)	Subjects, N (studies)	RR or SMD (95% CI)	Tit. Event proportion	Bio. Risk (95% CI)	Quality of evidence (GRADE)
Perioperative endpoints										
Plate breakage^a^	No studies					No studies				
Screw breakage^a^	No studies					155 (3)	Three studies, of which two only mentioned percentages of screw breakage without the total number of screws used			
Operation time^b^	No studies					No studies				
Handling by surgeon ^b^	No studies					No studies				
Short‐term follow‐up										
Malocclusion^a^	No studies					No studies				
Infection^a^	90 (2)	Two zero‐event studies				576 (9)	Nine studies, of which eight had zero events			
Swelling^a^	50 (1)	Single zero‐event study				No studies				
Abscess^a^	No studies					No studies				
Pain^a^	50 (1)	Single zero‐event study				No studies				
Analgesics used^a^	No studies					No studies				
MMO^b^	No studies					No studies				
Dehiscence^a^	No studies					226 (5)	Five zero‐event studies			
Plate exposure^a^	No studies					140 (4)	Four zero‐event studies			
Intermediate follow‐up										
Malunion^a^	No studies					93 (3)	Three zero‐event studies			
Mobility bone segments^a^	No studies					281 (6)	Six zero‐event studies			
Malocclusion^a^	No studies					No studies				
Pain^b^	50 (1)	Single zero‐event study				No studies				
MMO^b^	No studies					No studies				
TMJ‐dysfunction^a^	No studies					60 (2)	Two zero‐event studies			
Long‐term follow‐up										
Malocclusion^a^	No studies					204 (4)	Four zero‐event studies			
Pain^b^	50 (1)	Single zero‐event study				No studies				
MMO^b^	No studies					No studies				
TMJ‐dysfunction^a^	No studies					292 (2)	Two studies, of which one had zero events			
MFIQ^b^	No studies					No studies				
Abscess^a^	No studies					No studies				
Swelling^a^	50 (1)	Single zero‐event study				22 (1)	Single study			
Palpability plate/screws^a^	No studies					No studies				
Satisfaction^b^	No studies					No studies				
Overall follow‐up										
Symptomatic device removal^a^	No studies					No studies				
Total costs^b^	No studies					No studies				
Revision surgery (not device removal) ^a^	No studies					351 (3)	7.25 (0.91; 57.88)	0 per 1000	NA	Very low^c^, ^f^, ^g^
Maxillary horizontal relapse (adv)	50 (1)	Single study				288 (7)	+0.15 (−0.08; 0.39)	NA	NA	Very low^c^
Maxillary angular relapse (adv)	No studies					129 (4)	+0.07 (−0.41; 0.55)	NA	NA	Very low^c^, ^f^, ^g^
Maxillary horizontal relapse (sb)	No studies					47 (3)	−0.02 (−0.61; 0.57)	NA	NA	Very low^c^, ^f^
Maxillary angle relapse (sb)	No studies					No studies				
Maxillary vertical relapse (imp)	50 (1)	Single study				175 (5)	+0.35 (−0.13; 0.83)	NA	NA	Very low^c^, ^d^, ^f^
Maxillary vertical relapse (elong)	No studies					81 (3)	+0.31 (−0.23; 0.84)	NA	NA	Very low^c^, ^f^
Mandibular horizontal relapse (adv)	77 (2)	+0.16 (−0.39; 0.71)	NA	NA	Very low^c^, ^f^	129 (4)	+0.14 (−0.23; 0.51)	NA	NA	Very low^c^, ^f^
Mandibular horizontal relapse (sb)	No studies					195 (7)	+0.33 (−0.25; 0.91)	NA	NA	Very low^c^, ^d^, ^f^
Mandibular vertical relapse (adv)	37 (1)	Single study				69 (1)	Single study			
Mandibular vertical relapse (sb)	No studies					118 (4)	−0.25 (−0.66; 0.16)	NA	NA	Very low^c^, ^f^
Mandibular angular relapse (CW)	No studies					102 (4)	−0.79 (−1.40; −0.17)	NA	NA	Very low^c^, ^d^, ^f^
Mandibular angular relapse (CCW)	No studies					49 (3)	+0.34 (−0.23; 0.91)	NA	NA	Very low^c^, ^f^

Abbreviations: adv, advancement; Bio, biodegradable osteosynthesis; CCW, counter clockwise rotation; CW, clockwise rotation; elong, elongation; GRADE, Grades of Recommendation, Assessment, Development and Evaluation Working Group system; imp, impaction; NA, not applicable; RR, risk ratio (binary variables); sb, setback; SMD, standardized mean difference (continuous variables); Tit, titanium osteosynthesis.

^a^ Binary variable.

^b^ Continuous variable.

^c^ Downgraded one level due to high risks of bias identified across studies: majority of studies had high risk of bias.

^d^ Downgraded one level for inconsistency: substantial methodological or clinical heterogeneity that could not be accounted for in analyses.

^e^ Downgraded one level for indirectness: the evidence of the original manuscripts was more restrictive than the review question.

^f^ Downgraded one level for imprecision: limits of effect estimate confidence interval are not consistent (i.e., cover both benefit and harm).

^g^ Upgraded one level due to large effect (i.e., RR < 0.5 or RR > 2.0, or SMD < −0.8 or SMD > +0.8).

### Additional analyses

The sensitivity analyses with total zero event studies included showed no significant differences the conventional analyses. Additionally, a post‐hoc sensitivity analysis whereby one study was omitted at a time showed that excluding the study performed by Ueki et al. [19] significantly altered the overall effect estimate of vertical relapse after mandibular setback to a non‐significant difference between biodegradable and titanium osteosyntheses (both sensitivity analyses are available via the corresponding author). Sensitivity analyses showed that the effect estimates of all included RCTs did not significantly differ (i.e. overlapping 95% CI) whenever compared to effect estimates of only non‐high‐risk‐of‐bias RCTs (Figures S23 and S24). A meta‐regression analysis showed that all five domains and overall risk of bias did not have a significant effect on the effect estimate of symptomatic device removal (Table S7).

Trial sequential analyses revealed that the required information sizes were not achieved for the outcomes swelling (short‐long‐term follow‐up), dehiscence, mobility of bone segment, palpability of screws/plates, symptomatic device removal, and revision surgery. Also, no TSA‐boundaries were surpassed (Table S8) and, hence, TSA was not able to support the findings of conventional meta‐analyses for these outcomes.

## DISCUSSION

This meta‐analysis has shown that biodegradable and titanium osteosyntheses are equivalent in malunion rates, bone segments mobility, objective and subjective malocclusion, and maximal mouth opening at predefined time points after orthognathic surgery. Furthermore, we found no differences in swelling, pain, dehiscence, infection, plate exposure, plate and screw palpability, TMJ‐dysfunction, symptomatic device removal, and revision surgery rates (i.e., other than removal of plates and screws). Additionally, skeletal stability was similar in most types of orthognathic surgery except when assessing mandibular vertical relapse after setback, and mandibular angular relapse after clockwise (both in favour of biodegradable osteosyntheses) and counter‐clockwise‐rotation (in favour of titanium osteosyntheses). The operative time was significantly longer in the biodegradable compared to the titanium group.

Malunion at 6–12 weeks follow‐up was rare in both treatment groups. One study reported that a small proportion (3%) of patients treated with biodegradable osteosyntheses demonstrated malunion [55]. All other studies reported zero events of malunion in both treatment groups. This result, together with the non‐mobility of bone segments at 6–12 weeks follow‐up in most of the studies, emphasizes that both types of osteosyntheses are adequate for the fixation of maxillofacial osteotomies. Furthermore, low rates of objective and subjective malocclusion at both intermediate and long‐term follow‐up, and similar MFIQ scores at long‐term follow‐up (i.e., >5 years) were observed with both types of osteosyntheses.

Although skeletal stability was similar among both treatment groups after most orthognathic surgeries (Tables 4 and 5), even after surgical procedures which are known to exhibit a high degree of instability (e.g. maxillary setback), [63] significant differences were found between the biodegradable group and titanium group after mandibular setback (vertical relapse) and mandibular clockwise (angular relapse; both in favour of biodegradable osteosyntheses), and counter‐clockwise rotation (angular relapse; in favour of titanium osteosyntheses) surgery. The difference in vertical relapse after mandibular setback may have been due to the higher operative displacement in the titanium group [19, 20]. The sensitivity analysis showed that omitting Ueki et al. [19] (i.e. the study with the largest discrepancy in operative movement between both treatment groups; see Table S5) from the analysis diminished this significant difference between both treatment groups, indicating a non‐robust effect estimate of the initial analysis. Higher operative displacement results in less skeletal stability with greater skeletal relapse, and thus may be a confounding factor [64, 65]. We found no clear explanation for the difference in angular relapse after mandibular clockwise rotation in favour of biodegradable osteosyntheses. Ballon et al. [61] used an outer screw diameter of 2.5 and 2.0 mm with corresponding plates in the biodegradable and titanium group, respectively. Although the mechanical properties improve significantly when larger systems are used, [9] the mechanical properties of biodegradable systems remain lower than the corresponding titanium systems [9]. Skeletal stability was lower in the biodegradable group than the titanium group after counter‐clockwise rotation. One of the included RCT’s [19] described higher operative displacement in the biodegradable group, while another RCT reported similar operative displacement [20]. The higher operative displacement and discrepancy in duration of follow‐up (ranging from 6 months to 1 year) introduces heterogeneity in the meta‐analysis (*I^2^* = 75%). Given the confounding factors and substantial heterogeneity in these analyses, no firm conclusions regarding these differences can be drawn. Future researchers should focus on well‐defined in‐ and exclusion criteria to minimize heterogeneity (e.g. operative displacement) and on adequate follow‐up (i.e. ≥1 year) as most skeletal relapses occur in the first postoperative year [66, 67]. These measures would enable adequate pooling of future data.

Biodegradable osteosyntheses have been associated with foreign‐body reactions and, thus, continues to be a concern in the use of such osteosynthesis systems [2, 11]. The present review finds no significant differences regarding long‐term swelling and pain. A single study [56] assessed abscess formation after long‐term follow‐up and reported 2% vs. 0% abscess formation in the biodegradable and titanium group, respectively. Another study assessed plate and screw palpability during a follow‐up time >5 years and reported 42% vs. 8% cases after titanium and biodegradable osteosyntheses, respectively [2]. This finding is in line with the expectations given the resorbability of the latter osteosynthesis system. No significant difference was found in the symptomatic device removal rate between biodegradable and titanium osteosyntheses. The main reason for device removal in both groups was chronic infection or discomfort. A subgroup analysis comparing symptomatic device removal rates of both types of osteosyntheses according to the duration of follow‐up (i.e. ≤1 year and >1 year) showed that titanium osteosynthesis systems were favourable whenever follow‐up was longer than 1 year. Both these subgroup analyses indicate that biodegradable plates and screws, as opposed to only screws, tend to be removed after 1‐year follow‐up. This could be explained by the degradation process of biodegradable plates and screws which occurs in two phases, both consisting of hydrolysis [68]. Symptomatic swelling during the degradation of biodegradable osteosyntheses is in particular a concern during degradation (e.g. after >1‐year follow‐up) due to the attraction of water within the capsule surrounding the implant as well as foreign body reactions evoked by degradation products, and is less pronounced during the initial postoperative period (i.e. <1‐year follow‐up) [68, 69]. Discomfort was one of the main reasons for symptomatic device removal, especially of the biodegradable plate and screws after >1‐year follow‐up, which may have been due to their bulkiness. This effect might be less noticeable in cases where only screws or pins are used (i.e. less bulky). Therefore, future research should have at least a 1‐year follow‐up and should distinguish osteosyntheses with plates and screws vs. only screws to assess the symptomatic device removal rate appropriately.

The mandible is exposed to considerably higher biomechanical forces compared to the maxilla [70, 71]. Higher forces acting on the mandible may lead to loosening of screws and, thereafter, to inflammation especially in osteotomies because, unlike fractures, there is no interfragmentary stability [2]. Additionally, the lesser vascularity and morphology of the mandible could have unfavourable consequences on fixation and degradation of biodegradable osteosynthesis systems [2]. Of all included biodegradable osteosynthesis systems, three are certified to be used for mandibular osteotomies [72, 73, 74]. Instructions of all other included biodegradable systems state that usage of these systems in load‐bearing areas, including mandibular osteotomies, are contraindicated. Nonetheless, these biodegradable osteosynthesis systems have been applied off‐label in various studies that included patients treated with mandibular osteotomies [20, 43, 47, 50, 53, 59]. A subgroup analysis comparing maxillary vs mandibular vs. bimaxillary osteotomies showed no difference in symptomatic device removal between the types of osteotomies. However, this subgroup analysis showed a trend in favour of biodegradable osteosyntheses in maxillary osteotomies and titanium osteosyntheses in mandibular and bimaxillary osteotomies. Therefore, although the overall analysed symptomatic device removal rates were similar for titanium and biodegradable osteosyntheses (i.e. all patients undergoing orthognathic surgery analysed as a single group), biodegradable osteosyntheses could result in less symptomatic device removal after maxillary osteotomies and may thus be appealing for this specific subpopulation (i.e. maxillary osteotomies) due to possible lower symptomatic device removal rates compared to titanium osteosyntheses. However, the data for maxillary osteotomies in this subgroup analysis were derived from two small RCTs. Future studies should make this distinction thereby enabling a subgroup comparison with larger sample sizes.

Plate breakage occurred in none of the cases with titanium osteosyntheses while biodegradable plates broke in 0%–4% of the cases. Additionally, titanium screw breakage occurred only in one study in 3% of the cases [13] while biodegradable screws broke in 0%–12% of the cases of the included studies. These less favourable handling characteristics of biodegradable osteosyntheses are also expressed by surgeons (Table S5). The perioperative material complications, accompanied with the need to tap the burr hole and to heat the biodegradable plates to facilitate bending of the plate, are the main reasons that the operation time is significantly longer for biodegradable than titanium osteosynthesis systems. Surgeons do, however, state that more exposure to biodegradable systems could diminish these differences [62, 75].

None of the included studies assessed analgesic usage and angular relapse after maxillary setback. Also, data regarding abscess formation after long‐term follow‐up was rare but remains important as it could indicate a foreign‐body reaction. Future studies should include this endpoint in their assessment, preferably by taking a culture to exclude an infection as a cause. Furthermore, it is hypothesized that orthognathic surgery may cause biomechanical stress at the TMJ and could induce pathophysiological remodelling processes such as degenerative joint disease [76]. In this review, we found that 15% and 25% of the patients treated in one RCT [19] with biodegradable and titanium osteosyntheses, respectively, had complaints regarding TMJ‐function at 1‐year follow‐up. Additionally, patients in both treatment groups still had some mandibular function impairment at >5‐years follow‐up. Therefore, to adequately assess mandibular function after orthognathic surgery as well as to make comparison with healthy subjects possible, we advocate that future research should include TMJ‐function assessment and use validated questionnaires (e.g. MFIQ). Finally, a single RCT [57] assessed the total costs and reported that the titanium group's mean total costs at the 2 year follow‐up were slightly higher. The main reason was due to ‘absence of work’ after the surgical procedure due to worse MFIQ scores at 8 weeks follow‐up (i.e. indirect costs).

The most recent systematic review regarding the comparison of biodegradable vs. titanium osteosyntheses for orthognathic surgery was published in 2018, [23] but focused only on skeletal stability and failed to account for methodological (e.g. combining different study designs) and clinical heterogeneity (e.g. the authors included patients with cleft lip and palate). It is known that (i) the required amount of maxillary and mandibular movement is generally much larger in those patients compared to patients without a cleft, [77] (ii) their osteotomies are less stable compared to non‐cleft patients and therefore have more frequent and larger skeletal relapse after surgery, [78] and (iii) the frequency of secondary surgical intervention increases with increasing cleft severity (i.e. cleft lip and alveolus, unilateral cleft lip and palate, and bilateral cleft lip and palate) [79].

Another recent systematic review assessed the efficacy and morbidity of biodegradable compared to titanium osteosyntheses after maxillofacial trauma in a similar way as this review [10]. It was concluded that symptomatic device removal occurred significantly less often (RR 0.11; 95% CI 0.02; 0.57) and screw breakage (i.e. perioperative) significantly more often (RR 17.13, 95% CI: 2.19; 34.18) after biodegradable compared to titanium osteosyntheses [10]. An essential difference between both populations is that the fixation in trauma patients is supported by interfragmentary stability while this is, by definition, absent in osteotomies. Furthermore, trauma patients’ fractures are fixated into maximum occlusion whereas maximum occlusion in patients undergoing orthognathic surgery is reached after postoperative orthodontic treatment. Additionally, trauma patients are more often male using alcohol and tobacco compared to patients undergoing orthognathic surgery [80, 81, 82]. Both substances impair wound healing and reduce vascularity intra‐orally and thus disturb degradation and resorption of biodegradable systems. The different conclusions of the reviews of both populations emphasize that osteosyntheses in patients undergoing open reduction with internal fixation after maxillofacial trauma and patients undergoing orthognathic surgery should be considered as two different entities. Therefore, the results of any future research of both populations should be analysed separately.

None of the included RCTs were assessed as having overall low risk of bias. The quality of evidence (i.e. using the GRADE approach) of the assessed endpoints varied from very low to high quality. Downgrading the quality of evidence was predominantly caused by the presence of high risk of bias, imprecision and inconsistency of data (e.g. due to clinical heterogeneity but with insufficient evidence available to perform subgroup analyses). Endpoints with very low quality of evidence should be interpreted with caution and should not be used to make recommendations for clinical practice [25].

This is the most comprehensive systematic review to date comparing biodegradable with titanium osteosyntheses in orthognathic surgery. The strengths of this systematic review with meta‐analysis are the robust and transparent methodology used, based on a pre‐specified and ‐registered protocol, the Cochrane Handbook, and the PRISMA statement; a sensitive, thorough, and updated literature search without language or period restrictions; and inclusion of all relevant clinical endpoints. Furthermore, all stages of study selection and the data‐extraction were independently performed by two reviewers with excellent inter‐observer agreement. Additionally, the risk of bias and GRADE assessment were performed in duplicate. Finally, to increase the reliability of the conclusions drawn and to assess the required information size, TSA was performed.

The quality of studies included limits the outcomes of the current systematic review and thus biased effect estimates could not be excluded. Furthermore, clinical heterogeneity due to differences in biodegradable and titanium osteosynthesis systems used (e.g. composition and sizes), different procedures, differences in operative displacement, and differences in maxillomandibular fixation policies were present across studies. Preferably, we would perform subgroup analyses, but there was insufficient data reported in the studies to conduct these analyses. As a result of the presences of heterogeneity, we had to downgrade the quality of evidence of several endpoints in the GRADE assessment. Finally, despite multiple attempts to contact authors of original research papers, some data could not be retrieved and, thus, not be included in this review.

Currently, the main quality of the evidence varies from very low to moderate, and thus high‐quality research is needed. We acknowledge the fact that blinding (i.e. by surgeons and the outcome assessment) is not possible owing to the properties of titanium and biodegradable osteosynthesis systems. However, the main reasons for increasing the risk of bias was bias due to deviations from the intended intervention and bias due to missing outcome data. This emphasizes the need of pre‐specified, ‐registered, and well‐defined protocols of future RCTs. In particular, these protocols should focus on well‐defined (i) inclusion and exclusion criteria (e.g. separating the inclusion of maxillary and mandibular osteotomies) and (ii) endpoints to minimize reporting bias. Furthermore, (iii) appropriate follow‐up is advocated to minimize attrition bias (e.g. >1 year follow‐up for symptomatic device removal and skeletal stability) and (iv) indications for device removal should be clearly defined and followed to reduce detection bias. Additionally, since data regarding three‐dimensional analysis of patients undergoing orthognathic surgery is growing, they could and should be used in the analyses of skeletal stability. Also, as the patient's opinion regarding outcomes is of high importance, patient reported outcomes (e.g. MFIQ, subjective malocclusion) should be assessed. Moreover, reporting of patient and surgical characteristics, and outcomes, including details regarding the usage of alcohol and tobacco, the used osteosynthesis systems with compositions and sizes, and the maxillomandibular fixation, orthodontic treatment, and postoperative dietary policies, should be improved. Future studies should also include cost‐effectiveness as the outcome measure, including primary (i.e. perioperative) and secondary costs (e.g. additional interventions, travelling expenses, absence of work). Finally, RCTs should adhere to the CONSORT guidelines to assure high‐quality reporting, [83] minimize reporting bias, and enable assessment and pooling of future data.

Considering the qualitative review and meta‐analyses, biodegradable and titanium osteosyntheses are equivalent in efficacy and morbidity of fixation after orthognathic surgery. Perioperative plate and screw breakage, however, occur more often with biodegradable relative to titanium systems, and the operative time of the former is longer. Symptomatic device removal rate is similar among both groups. Skeletal stability is similar in most types of orthognathic surgery after using both types of osteosyntheses. Biodegradable osteosyntheses can serve as a valid alternative whenever this is preferred by surgeons or patients. Due to the quality of evidence, high‐quality studies are necessary to elucidate the full potential of biodegradable osteosyntheses.

## CONFLICTS OF INTERESTS

The authors state that they have no conflicts of interest.

## AUTHOR CONTRIBUTIONS

**B.G., N.B.vB., P.U.D., A.V., R.B., B.vM.:** Conceptualization, Methodology, Writing ‐ review & editing; **B.G.:** Software, Formal analysis, Investigation, Writing ‐ original draft, Visualization; **N.B.vB., P.U.D.:** Validation; **N.B.vB., P.U.D., A.V., R.B., B.vM.:** Supervision. **B.G., N.B.vB., P.U.D., A.V., R.B., B.vM.:** approved the submitted version of the manuscript, have agreed to be personally accountable for the author's contributions, and ensure that questions related to the accuracy and integrity will be appropriately investigated.

## Supporting information

Appendix S1
