# Supplementary material for: Efficacy and morbidity of biodegradable versus titanium osteosyntheses in orthognathic surgery: A systematic review with meta‐analysis and trial sequential analysis
Source: Eur J Oral Sci. 2021 Jun 15;129(5):e12800. doi: 10.1111/eos.12800 (PMC8596673; doi:10.1111/eos.12800)
Supplement: Supplementary file 1 — Appendix S1 [file EOS-129-0-s001.pdf]

SUPPORTING INFORMATION

Efficacy and morbidity of biodegradable versus titanium osteosyntheses in orthognathic surgery: a systematic review with meta-analysis and trial sequential analysis

BARZI GAREB, NICO B. VAN BAKELEN, PIETER U. DIJKSTRA, ARJAN VISSINK, RUUD R.M. BOS, BAUCKE VAN MINNEN

University of Groningen, University Medical Center Groningen, Groningen, The Netherlands

Supplementary Tables:

Table S1: Electronic databases with the corresponding search details (11 February 2021).

Table S2: Endpoints divided into five time units.

Table S3: List with contacted authors of original articles.

Table S4: Excluded articles with reasons for exclusion after full-text screening.

Table S5: All assessed endpoints of the included studies, except skeletal stability.

Table S6: Operative displacement and relapse.

Table S7: Results of univariable meta-regression analyses to analyze the effect of risk of bias items on the log risk ratio of symptomatic device removal using a random effects model.

Table S8: Input and results of the trial sequential analyses using the random-effects (DerSimonian-Laird) model with the corresponding interpretations.

Supplementary Figures:

Figure S1: Example graph with explanation of the trial sequential analysis.

Figure S2: Forest plot of the endpoint operative time in minutes. *SMD, standardised mean difference; 95%-CI, 95% confidence interval.*

Figure S3: Forest plot of the endpoint swelling (<4 weeks follow-up) stratified by study design. *RCT, randomised controlled trials; Prosp. CS, prospective cohort studies; RR, risk ratio; 95%-CI, 95% confidence interval.*

Figure S4: Forest plot of the endpoint dehiscence (<4 weeks follow-up) stratified by study design. *RCT, randomised controlled trials; Retrosp. CS, retrospective cohort studies; RR, risk ratio; 95%-CI, 95% confidence interval.*

Figure S5: Forest plot of the endpoint pain (6-12 weeks follow-up) stratified by study design. *RCT, randomised controlled trials; SMD, standardised mean difference; 95%-CI, 95% confidence interval.*

Figure S6: Forest plot of the endpoint pain (>12 weeks follow-up) stratified by study design. *RCT, randomised controlled trials; SMD, standardised mean difference; 95%-CI, 95% confidence interval.*

Figure S7: Forest plot of the endpoint maximum mouth opening (>12 weeks follow-up) stratified by study design. *RCT, randomised controlled trials; SMD, standardised mean difference; 95%-CI, 95% confidence interval.*

Figure S8: Forest plot of the endpoint symptomatic device removal (overall follow-up) of studies stratified maxillary, mandibular, and bimaxillary osteotomies. *RR, risk ratio; 95%-CI, 95% confidence interval.*

Figure S9: Forest plot of the endpoint symptomatic device removal (overall follow-up) of studies including osteosynthesis by plates and screws versus only screws. *RR, risk ratio; 95%-CI, 95% confidence interval.*

Figure S10: Forest plot of the endpoint symptomatic device removal (overall follow-up) stratified by ≤1-year and >1-year follow-up. *RR, risk ratio; 95%-CI, 95% confidence interval.*

Figure S11: Forest plot of the endpoint maxillary horizontal relapse after maxillary advancement stratified by study design (overall follow-up). *RCT, randomised controlled trials; Retrosp. CS, retrospective cohort studies; SMD, standardised mean difference; 95%-CI, 95% confidence interval.*

Figure S12: Forest plot of the endpoint maxillary angular relapse after maxillary advancement stratified by study design (overall follow-up). *Retrosp. CS, retrospective cohort studies; SMD, standardised mean difference; 95%-CI, 95% confidence interval.*

Figure S13: Forest plot of the endpoint maxillary horizontal relapse after maxillary setback stratified by study design (overall follow-up). *Retrosp. CS, retrospective cohort studies; SMD, standardised mean difference; 95%-CI, 95% confidence interval.*

Figure S14: Forest plot of the endpoint maxillary vertical relapse after maxillary impaction stratified by study design (overall follow-up). *RCT, randomised controlled trials; Retrosp. CS, retrospective cohort studies; SMD, standardised mean difference; 95%-CI, 95% confidence interval.*

Figure S15: Forest plot of the endpoint maxillary vertical relapse after maxillary elongation stratified by study design (overall follow-up). *Retrosp. CS, retrospective cohort studies; SMD, standardised mean difference; 95%-CI, 95% confidence interval.*

Figure S16: Forest plot of the endpoint mandibular horizontal relapse after mandibular advancement stratified by study design (overall follow-up). *Prosp. CS, prospective cohort studies; Retrosp. CS, retrospective cohort studies; SMD, standardised mean difference; 95%-CI, 95% confidence interval.*

Figure S17: Forest plot of the endpoint mandibular horizontal relapse after mandibular setback stratified by study design (overall follow-up). *RCT, randomised controlled trials; Retrosp. CS, retrospective cohort studies; SMD, standardised mean difference; 95%-CI, 95% confidence interval.*

Figure S18: Forest plot of the endpoint mandibular horizontal relapse after mandibular setback of studies including osteosynthesis by plates and screws versus only screws (overall follow-up). *SMD, standardised mean difference; 95%-CI, 95% confidence interval.*

Figure S19: Forest plot of the endpoint mandibular vertical relapse after mandibular setback stratified by study design (overall follow-up). *RCT, randomised controlled trials; Retrosp. CS, retrospective cohort studies; SMD, standardised mean difference; 95%-CI, 95% confidence interval.*

Figure S20: Forest plot of the endpoint mandibular vertical relapse after mandibular setback of studies including osteosynthesis by plates and screws versus only screws (overall follow-up). *SMD, standardised mean difference; 95%-CI, 95% confidence interval.*

Figure S21: Forest plot of the endpoint mandibular angular relapse after mandibular clockwise rotation stratified by study design (overall follow-up). *Retrosp. CS, retrospective cohort studies; SMD, standardised mean difference; 95%-CI, 95% confidence interval.*

Figure S22: Forest plot of the endpoint mandibular angular relapse after mandibular counter clockwise rotation stratified by study design (overall follow-up). *RCT, randomised controlled trials; Retrosp. CS, retrospective cohort studies; SMD, standardised mean difference; 95%-CI, 95% confidence interval.*

Figure S23: Sensitivity analysis with summary risk ratios for all pooled outcomes according to the inclusion of all randomized controlled trials and non-high-risk-of-bias RCTs only. *RCT, randomised controlled trials; RR, risk ratio; 95%-CI, 95% confidence interval.*

Figure S24: Sensitivity analysis with summary standardized mean differences for all pooled outcomes according to the inclusion of all randomized controlled trials and non-high-risk-of-bias RCTs only. *RCT, randomised controlled trials; SMD, standardised mean difference; 95%-CI, 95% confidence interval.*

Table S1: Electronic databases with the corresponding search details (11 February 2021).

Database	Search terms	Hits
PubMed (http://www.ncbi.nlm.nih.gov/pubmed/)	("Orthognathic Surgery"[Mesh] OR "Orthognathic Surgical Procedures"[Mesh] OR "Osteotomy, Le Fort"[Mesh] OR "Osteotomy, Sagittal Split Ramus"[Mesh] OR "Mandibular Advancement"[Mesh] OR "Facial Bones/surgery"[Mesh] OR "Facial Injuries"[Mesh:NoExp] OR "Maxillofacial Injuries"[Mesh] OR "Maxillofacial Abnormalities"[Mesh] OR "Malocclusion/surgery"[Mesh] OR maxill*[tiab] OR mandib*[tiab] OR jaw[tiab] OR orthognat*[tiab] OR craniofac*[tiab] OR craniomaxil*[tiab] OR retrognat*[tiab] OR orthodont*[tiab] OR zygom*[tiab] OR split ramus[tiab] OR "Facial injuries"[MeSH] OR ((orbit*[tiab] OR facial[tiab] OR face[tiab] OR nose[tiab] OR nasal[tiab]) AND (fract*[tiab] OR injur*[tiab] OR reconstruct*[tiab])))<br>AND ("Absorbable Implants"[Mesh] OR "Internal Fixators"[Mesh] OR "Fracture Fixation, Internal"[Mesh] OR plate*[tiab] OR screw*[tiab] OR miniscrew*[tiab] OR miniplate*[tiab] OR implant*[tiab] OR osteosynth*[tiab] OR osseointegrat*[tiab] OR osteofixat*[tiab] OR osteotom*[tiab] OR fixat*[tiab])<br>AND ("Absorbable Implants"[Mesh] OR bioresorb*[tiab] OR biodegrad*[tiab] OR bioabsorb*[tiab] OR bioadsorb*[tiab] OR absorb*[tiab] OR resorb*[tiab] OR adsorb*[tiab] OR "Lactic acid"[MeSH] OR lactic acid[tiab] OR "Polyglycolic acid"[MeSH] OR polyglycolic acid[tiab] OR "Hydroxyapatites"[MeSH] OR hydroxyapatite[tiab] OR biologically inert[tiab])<br>NOT ("Case Reports" [Publication Type] OR "Review" [Publication Type])<br>NOT ("Animals"[Mesh] NOT "Humans"[Mesh])	2535
EMBASE (http://www.embase.com/home)	('craniofacial surgery'/de OR 'cranioplasty'/exp OR 'face surgery'/de OR 'maxillofacial surgery'/exp OR 'nose surgery'/exp OR 'orthognathic surgery'/exp OR 'orbit reconstruction'/exp OR 'maxillofacial injury'/de OR 'skull injury'/exp OR 'skull'/exp OR 'face fracture'/exp OR 'skull malformation'/exp/dm_su OR 'craniofacial malformation'/exp OR 'face malformation'/dm_su OR 'malocclusion'/exp/dm_su OR (maxill* OR mandib* OR jaw OR orthognat* OR craniofac* OR craniomaxil* OR retrognat* OR orthodont* OR zygom* OR 'split ramus' OR ((orbit* OR facial OR face OR nose OR nasal) AND (fract* OR injur* OR reconstruct*))):ab,ti)<br>AND ('bone plate'/exp OR 'bone screw'/exp OR 'internal fixator'/exp OR 'fracture fixation'/exp OR 'bioabsorbable screw'/exp OR 'biodegradable screw'/exp OR 'biodegradable implant'/exp OR 'orthopedic fixation device'/de OR (plate* OR screw* OR miniscrew* OR miniplate* OR implant* OR osteosynth* OR osseointegrat* OR osteofixat* OR osteotom* OR fixat*):ab,ti)<br>AND ('biodegradable implant'/exp OR 'bioabsorbable screw'/exp OR 'biodegradable screw'/exp OR 'lactic acid'/exp/mj OR 'polyglycolic acid'/exp/mj OR 'hydroxyapatite'/exp/mj OR 'biosorbent'/exp OR (bioresorb* OR biodegrad* OR bioabsorb* OR bioadsorb* OR absorb* OR resorb* OR adsorb* OR 'lactic acid' OR 'polyglycolic acid' OR hydroxyapatite OR 'biologically inert'):ab,ti)<br>NOT (('animal'/exp OR 'nonhuman'/exp) NOT 'human'/exp)<br>NOT ('review'/exp OR 'case report'/exp OR 'conference abstract'/it)	2656
Cochrane Central Register of Controlled Trials (www.thecochranelibrary.com)	(maxill* OR mandib* OR jaw OR orthognat* OR craniofac* OR craniomaxil* OR retrognat* OR orthodont* OR osteotom* OR zygom* OR "split ramus" OR (malocclus* AND surg*) OR ((orbit* OR facial OR face OR nose OR nasal) AND (fract* OR injur* OR reconstruct* OR surg*)))<br>AND (plate* OR screw* OR miniscrew* OR miniplate* OR implant* OR osteosynth* OR osseointegrat* OR osteofixat* OR osteotom* OR fixat*)<br>AND (bioresorb* OR biodegrad* OR bioabsorb* OR bioadsorb* OR absorb* OR resorb* OR adsorb* OR "Lactic acid" OR "Polyglycolic acid" OR Hydroxyapatite* OR "biologically inert")	663
Web of Science (www.webofknowledge.com)	TS=(maxill* OR mandib* OR jaw OR orthognat* OR craniofac* OR craniomaxil* OR retrognat* OR orthodont* OR osteotom* OR zygom* OR "split ramus" OR (malocclus* AND surg*) OR ((orbit* OR facial OR face OR nose OR nasal) AND (fract* OR injur* OR reconstruct* OR surg*)))<br>AND<br>TS=(plate* OR screw* OR miniscrew* OR miniplate* OR implant* OR osteosynth* OR osseointegrat* OR osteofixat* OR osteotom* OR fixat*)<br>AND<br>TS=(bioresorb* OR biodegrad* OR bioabsorb* OR bioadsorb* OR absorb* OR resorb* OR adsorb* OR "Lactic acid" OR "Polyglycolic acid" OR Hydroxyapatite* OR "biologically inert")<br>NOT<br>DT=(review OR "meeting abstract")	7820
EBSCOhost (search.ebscohost.com)<br>Databases:	((maxill* OR mandib* OR jaw OR orthognat* OR craniofac* OR craniomaxil* OR retrognat* OR orthodont* OR osteotom* OR zygom* OR "split ramus" OR (malocclus* AND surg*) OR ((orbit* OR facial OR face OR nose OR nasal) AND (fract* OR injur* OR reconstruct* OR surg*)))<br>AND	2608

Academic search Premier, Business Source Premier, Military & Government Collection, and CINAHL	(plate* OR screw* OR miniscrew* OR miniplate* OR implant* OR osteosynth* OR osseointegrat* OR osteofixat* OR osteotom* OR fixat*)<br>AND<br>(bioresorb* OR biodegrad* OR bioabsorb* OR bioadsorb* OR absorb* OR resorb* OR adsorb* OR "Lactic acid" OR "Polyglycolic acid" OR Hydroxyapatite* OR “biologically inert”))	
Scopus (www.scopus.com/)	TITLE-ABS-KEY(maxill* OR mandib* OR jaw OR orthognat* OR craniofac* OR craniomaxil* OR retrognat* OR orthodont* OR osteotom* OR zygom* OR “split ramus” OR (malocclus* AND surg*) OR ((orbit* OR facial OR face OR nose OR nasal) AND (fract* OR injur* OR reconstruct* OR surg*)))<br>AND<br>TITLE-ABS-KEY(plate* OR screw* OR miniscrew* OR miniplate* OR implant* OR osteosynth* OR osseointegrat* OR osteofixat* OR osteotom* OR fixat*)<br>AND<br>TITLE-ABS-KEY(bioresorb* OR biodegrad* OR bioabsorb* OR bioadsorb* OR absorb* OR resorb* OR adsorb* OR "Lactic acid" OR "Polyglycolic acid" OR Hydroxyapatite* OR “biologically inert”)	7981
African Journals Online (www.ajol.info/)	(maxillary OR mandibular OR orthognathic OR craniofacial OR craniomaxillofacial) AND (bioresorbable OR biodegradable)	41
OpenGrey (www.opengrey.eu)	((maxill* OR mandib* OR jaw OR orthognat* OR craniofac* OR craniomaxil* OR retrognat* OR orthodont* OR osteotom* OR zygom* OR “split ramus” OR (malocclus* AND surg*) OR ((orbit* OR facial OR face OR nose OR nasal) AND (fract* OR injur* OR reconstruct* OR surg*)))<br>AND<br>(bioresorb* OR biodegrad* OR bioabsorb* OR bioadsorb* OR absorb* OR resorb* OR adsorb* OR "Lactic acid" OR "Polyglycolic acid" OR Hydroxyapatite* OR “biologically inert”))	40
ClinicalTrials.gov	Condition: (maxillary OR mandibular OR orthognathic OR craniofacial OR craniomaxillofacial)<br>Other terms: (bioresorbable OR biodegradable)	5

Table S2: Endpoints divided into five time units.

Time unit	Endpoints
**Perioperative**	Plate and/or screw breakage, operation time, and handling by surgeon
**Short-term (0-4 weeks; soft tissue healing)**	Infection, dehiscence, malocclusion, pain, swelling, plate exposure, MMO, abscess, and analgesics used
**Intermediate follow-up (6 – 12 weeks; bone healing)**	Malunion, mobility of bone segments, malocclusion, MMO, TMJ-dysfunction, and pain
**Long-term follow-up (>12 weeks; degradation effects)**	Palpability of plate and screws, malocclusion, pain, swelling, satisfaction, TMJ-dysfunction, MMO, abscess, and MFIQ
**Overall**	Skeletal stability (i.e., skeletal relapse), symptomatic device removal, additional surgery (not device removal), and total costs

MMO: maximal mouth opening; TMJ-dysfunction: temporomandibular joint dysfunction; MFIQ: Mandibular Function Impairment Questionnaire.

Table S3: List with contacted authors of original articles.

Study	Dates of contact	Reasons for contact	Responses
Matthews et al. (2003)[1]	19 May 2018, 29 May 2018, and 22 August 2018.	1. Details regarding study design<br>2. Details regarding the distribution of data of the outcomes 'operative displacement' and 'relapse'<br>3. Details regarding the allocation of two patients with TMJ-dysfunction.	1. "It was a randomised study using odd/even registration numbers that are linked to concealed envelops."<br>2. "Unfortunately, I don't have access to the original data, it has been more than 15 years since we conducted this study."<br>3. "Apology, I can't remember."
Cheung et al. (2008)[2]	29 May 2018, 1 August 2018, 22 August 2018, 19 October 2018	1. All reported data are presented in figures only. No numbers are available to include in the meta-analyses.	No response
Stockmann et al. (2010)[3]	29 May 2018, 1 August 2018, 10 August 2018	1. Data not reported per treatment group<br>2. Definition of relapse (i.e., it currently is a binary variable).	No response
Tuovinen et al. (2010)[4]	23 August 2018, 24 August 2018, and 19 October 2018	1. Only P-values of difference between relapse of both treatment groups are reported, not the amount of relapse itself.	1. "Thank you for your interest concerning my report. I have not found my raw data yet. I'll keep on digging in my old cd-copies. My computer has been changed several times and we have been moving to three different location since that report and in the archive where the data should be I could not find it. I'll continue to look for the data."<br>2. "I have not found SPSS files but all handmade (written) measurements can be find, but it would be quite a job to copy and explain my notes and put them again in

			SPSS. Attached one example of measurements."<br>3. No response after asking for all the data.
Landes et al. (2006)[5]	29 May 2018, 22 August 2018, 19 October 2018	1. Details regarding the absolute number of screw and plate breakage per treatment group.	No response
Paeng et al. (2012)[6]	29 May 2018, 22 August 2018, and 19 October 2018	1. Details regarding the single patient that demonstrated infection after 7 days follow-up: which treatment group?	No response

Table S4: Excluded articles with reasons for exclusion after full-text screening.

Author (year)	Reason for exclusion	Reference
Ahmed et al. (2013)	Surgical procedure not relevant for this review	[7]
Arshad et al. (2019)	Surgical procedure not relevant for this review	[8]
Arya et al. (2020)	Surgical procedure not relevant for this review	[9]
Bekal et al. (2017)	Surgical procedure not relevant for this review	[10]
Bell et al. (2006)	Surgical procedure not relevant for this review	[11]
Bhatt et al. (2010)	Surgical procedure not relevant for this review	[12]
Bhatt et al. (2015)	Surgical procedure not relevant for this review	[13]
Bohm et al. (1998)	Surgical procedure not relevant for this review	[14]
Bouletreau et al. (2005)	Both groups consist of biodegradable and titanium osteosynthesis	[15]
Burlini et al. (2015)	Surgical procedure not relevant for this review	[16]
Champy et al. (1992)	No control group	[17]
Wang et al. (2013)	Surgical procedure not relevant for this review	[18]
Fakourand et al. (2012	Surgical procedure not relevant for this review	[19]
Filinte et al. (2015)	Surgical procedure not relevant for this review	[20]
Fuente del Campo et al. (1996)	No control group; Biodegradable plates with titanium screws used	[21]
Hashiba et al. (2007)	No relevant endpoints for this review	[22]
Ho et al. (2011)	No pure biodegradable group, only titanium or mixed groups	[23]
Hwang et al. (2017)	No pure biodegradable group, only titanium or mixed groups	[24]
Iatrou et al. (2010)	Surgical procedure not relevant for this review	[25]
Illi et al. (1989)	Children with syndromic disorders included	[26]
Imola et al. (2002)	Review paper	[27]
Janickova et al. (2018)	Surgical procedure not relevant for this review	[28]
Kallela et al. (1999)	Review paper	[29]
Kang et al. (2014)	Surgical procedure not relevant for this review	[30]
Kim et al. (2018)	Surgical procedure not relevant for this review	[31]
Kobayashi et al. (2004)	No control group	[32]
Kretschmer et al. (2011)	Surgical procedure not relevant for this review	[33]
Landes et al. (2014)	Patients with cleft lip and palate included	[34]
Landes et al. (2015)	No control group	[35]
Lee et al. (2010)	Surgical procedure not relevant for this review	[36]
Lee et al. (2014)	Surgical procedure not relevant for this review	[37]
Lee et al. (2014)	Endpoint not relevant for this review	[38]
Leno et al. (2017)	Surgical procedure not relevant for this review	[39]

Leonhardt et al. (2008)	Surgical procedure not relevant for this review	[40]
Lim et al. (2014)	Surgical procedure not relevant for this review	[41]
Liu et al. (2016)	Surgical procedure not relevant for this review	[42]
Mahmoud et al. (2016)	Surgical procedure not relevant for this review	[43]
Menon et al. (2007)	Surgical procedure not relevant for this review	[44]
Menon et al. (2012)	Surgical procedure not relevant for this review	[45]
Netto et al. (2013)	Surgical procedure not relevant for this review	[46]
Obwegeser et al. (1994)	No biodegradable osteosynthesis used, only biodegradable sutures	[47]
Park et al. (2005)	Surgical procedure not relevant for this review	[48]
Park et al. (2011)	Surgical procedure not relevant for this review	[49]
Pistner et al. (1991)	Review paper	[50]
Qiu et al. (2015)	Surgical procedure not relevant for this review	[51]
Stuck et al. (2011)	Review paper	[52]
Sukegawa et al. (2016)	Surgical procedure not relevant for this review	[53]
Tan et al. (2011)	Surgical procedure not relevant for this review	[54]
Tripathi et al. (2013)	Surgical procedure not relevant for this review	[55]
Ueki et al. (2011b)	Does not fulfill inclusion criteria	[56]
Ueki et al. (2015b)	Does not fulfill inclusion criteria	[57]
Ueki et al. (2017)	Does not fulfill inclusion criteria	[58]
Wittwer et al. (2006)	Surgical procedure not relevant for this review	[59]
Wu et al. (2017)	Surgical procedure not relevant for this review	[60]
Zheng et al. (2001)	No control group	[61]

Table S5: All assessed endpoints of the included studies, except skeletal stability.

Study name (year)	Osteosynthesis system	Plate breakage (%)	Screw breakage (%)	Operation time (mean±SD, in minutes)[a]	Handling by surgeon (0: worst; 10: excellent)	Infection (%)	Swelling (%)	Abscess (%)	Pain (%)	MMO (%)	Dehiscence (%)	Plate exposure (%)	Malunion (%)	Mobility segments (%)	Malocclusion (%)	Pain (%)	MMO (mean±SD)	TMJ-dysfunction (%)	Malocclusion (%)	Pain (%)	MMO (mean±SD)	TMJ-dysfunction (%)	MFIQ (median, IQR)	Abscess (%)	Swelling (%)	Palpability screw/plate (%)	Satisfaction (%)	Symptomatic plate/screw removal (%)	Costs (direct and indirect; mean±SD)[a]	Revision surgery (not device removal; %)	Skeletal stability assessed (yes)
		Perioperative endpoints				Short-term follow-up							Intermediate follow-up						Long-term follow-up									Overall follow-up			
													Randomised controlled trials																		
**Matthews (2003)**[1]	B	0	0			0						9.1						0								9.1				0	x
	T	0	0			0						0						0								0				0	x
**Norholt (2004)**[62]	B				82.1[b]	0	42.9		25		3.6	0		22.2		18.5				20					4	12		0		8.3	x
	T				100[b]	3.7	40.7		29.6		0	0		10.7		21.4				28.6					0	61.9		14.3		0	x
**Cheung (2004)**[63]	B	4.2[*]	10.9[#]			3.3			3.63± 2.27[a†]		6.7			6.7		1.2± 1.35[a†]				0.46± 1.10[a†]							8.63± 1.44[a†]	20			
	T	0[*]	0[#]			0			4.40± 2.29[a†]		10			10		1.47± 1.50[a†]				0.67± 1.13[a†]							8.50± 1.84[a†]	10			
**Ueki (2005)**[64]	B					0					0	0[c]	0	0					0 (O)	0.8± 1.8[a]	-3.40± 2.26[f]	15									x
	T					0					0	0[c]	0	0					0 (O)	1.0± 2.1[a]	-5.60± 1.86[f]	25									x
**Cheung (2008)**[2]	B								NNA					NNA		NNA				NNA						NNA					x
	T								NNA					NNA		NNA				NNA						NNA					x

Study name (year)	Osteosynthesis system	Plate breakage (%)	Screw breakage (%)	Operation time (mean±SD, in minutes)[a]	Handling by surgeon (0: worst; 10: excellent)	Infection (%)	Swelling (%)	Abscess (%)	Pain (%)	MMO (%)	Dehiscence (%)	Plate exposure (%)	Malunion (%)	Mobility segments (%)	Malocclusion (%)	Pain (%)	MMO (mean±SD)	TMJ-dysfunction (%)	Malocclusion (%)	Pain (%)	MMO (mean±SD)	TMJ-dysfunction (%)	MFIQ (median, IQR)	Abscess (%)	Swelling (%)	Palpability screw/plate (%)	Satisfaction (%)	Symptomatic plate/screw removal (%)	Costs (direct and indirect; mean±SD)[a]	Revision surgery (not device removal; %)	Skeletal stability assessed (yes)
		**Perioperative endpoints**				**Short-term follow-up**						**Intermediate follow-up**							**Long-term follow-up**									**Overall follow-up**			
**Park (2010)**[65]	B																														x
	T																														x
**Stockmann (2010)**[3]	B		9.1	+36[d]		3				NNA	3	NNA					NNA		5.9 (O)		NNA						62	0			
	T		3	NNA[d]		3				NNA	0	0[c]					NNA		5.9 (O)		NNA							3			
**Tuovinen (2010)**[4]	B					0																						2		2	x
	T					0																						4		4	x
**Buijs (2012)**[66]	B	0	0	137± 59	7.6± 0.7[a]		17.1	11.8			3.9		2.6		9.2 (O)	5.15± 8.9[a]												1.3		0	
	T	0	0	116± 64	8.5± 1.2[a]		7.3	4.8			1.6		0		12.9 (O)	4.7± 10.6[a]												1.6		0.8	
**Yoshioka (2012)**[67]	B	2.7				0																				1.8		5.5		0	
	T	0				1.1																				0		3.3		0	
**Bakelen (2013)**[68],[e]	B																		7.5 & 11.3 (O)	2±9 & 2±6			17 (17-23) & 17 (17-19)	3 & 1.9	9 & 5.7	50.7 & 47.2		24		2.5	

Study name (year)	Osteosynthesis system	Plate breakage (%)	Screw breakage (%)	Operation time (mean±SD, in minutes)[a]	Handling by surgeon (0: worst; 10: excellent)	Infection (%)	Swelling (%)	Abscess (%)	Pain (%)	MMO (%)	Dehiscence (%)	Plate exposure (%)	Malunion (%)	Mobility segments (%)	Malocclusion (%)	Pain (%)	MMO (mean±SD)	TMJ-dysfunction (%)	Malocclusion (%)	Pain (%)	MMO (mean±SD)	TMJ-dysfunction (%)	MFIQ (median, IQR)	Abscess (%)	Swelling (%)	Palpability screw/plate (%)	Satisfaction (%)	Symptomatic plate/screw removal (%)	Costs (direct and indirect; mean±SD)[a]	Revision surgery (not device removal; %)	Skeletal stability assessed (yes)
		Perioperative endpoints				Short-term follow-up					Intermediate follow-up								Long-term follow-up									Overall follow-up			
	T																		12.1 & 12.2 (O)	1±7 & 2±9			21 (17-27) & 19 (17-23)	2.6 & 0	3.4 & 0	34.5 & 47.8		8		1.6	
**Yu et al. (2014)**[69]	B					16															-0.31±6.80[f]							0			
	T					13.7															-1.86±7.51[f]							3.9			
**Bakelen (2015)**[70]	B																												6589±3492		
	T																												6787±5014		
**Gareb (2017)**[71]	B																		11.8 (S)	7.0±18.7[a]			18 (17-21)		5.9	7.8		29.1			
	T																		14.8 (S)	5.7±17.6[a]			18 (17-21)		2.5	42		15.3			

		Perioperative endpoints				Short-term follow-up							Intermediate follow-up						Long-term follow-up									Overall follow-up			
Study name (year)	Osteosynthesis system	Plate breakage (%)	Screw breakage (%)	Operation time (mean±SD, in minutes)[a]	Handling by surgeon (0: worst; 10: excellent)	Infection (%)	Swelling (%)	Abscess (%)	Pain (%)	MMO (%)	Dehiscence (%)	Plate exposure (%)	Malunion (%)	Mobility segments (%)	Malocclusion (%)	Pain (%)	MMO (mean±SD)	TMJ-dysfunction (%)	Malocclusion (%)	Pain (%)	MMO (mean±SD)	TMJ-dysfunction (%)	MFIQ (median, IQR)	Abscess (%)	Swelling (%)	Palpability screw/plate (%)	Satisfaction (%)	Symptomatic plate/screw removal (%)	Costs (direct and indirect; mean±SD)[a]	Revision surgery (not device removal; %)	Skeletal stability assessed (yes)
Prospective cohort studies																															
**Ferretti (2002)**[72]	B					0																									x
	T					0																									x
**Dhol (2008)**[73]	B					0	0		0							0				0					0						x
	T					0	0		0							0				0					0						x
**Bakelen (2014)**[74]	B																														x
	T																														x
Retrospective cohort studies																															
**Harada (1997)**[75]	B																	0				0									x
	T																	0				0									x
**Costa (2006)**[76]	B					0					0	0[c]	0	0						20					20					20	x
	T					0					0	0[c]	0	0						0					0					0	x
**Landes (2006)**[5]	B		5[#]											0																	x
	T		0[#]											0																	x
**Turvey (2006)**[77]	B																														x
	T																														x

Study name (year)	Osteosynthesis system	Plate breakage (%)	Screw breakage (%)	Operation time (mean±SD, in minutes)[a]	Handling by surgeon (0: worst; 10: excellent)	Infection (%)	Swelling (%)	Abscess (%)	Pain (%)	MMO (%)	Dehiscence (%)	Plate exposure (%)	Malunion (%)	Mobility segments (%)	Malocclusion (%)	Pain (%)	MMO (mean±SD)	TMJ-dysfunction (%)	Malocclusion (%)	Pain (%)	MMO (mean±SD)	TMJ-dysfunction (%)	MFIQ (median, IQR)	Abscess (%)	Swelling (%)	Palpability screw/plate (%)	Satisfaction (%)	Symptomatic plate/screw removal (%)	Costs (direct and indirect; mean±SD)[a]	Revision surgery (not device removal; %)	Skeletal stability assessed (yes)
		Perioperative endpoints				Short-term follow-up							Intermediate follow-up							Long-term follow-up								Overall follow-up			
**Ueki (2006a and b)**[78]	B					a & b: 0					a & b: 0	a & b: 0[c]							a & b: 0												x
	T					a & b: 0					a & b: 0	a & b: 0[c]							a & b: 0												x
**Landes (2007)**[79]	B		12[#]											0																	x
	T		0[#]											0																	x
**Ueki (2009)**[80]	B					0					0	0[c]	0	0					0												
	T					0					0	0[c]	0	0					0												
**Ahn (2010)**[81]	B					4.2																5.8								2.5	
	T					0																6.6								0	
**Choi (2010)**[82]	B																														x
	T																														x
**Ueki (2011)**[83]	B					0												0													x
	T					0												0													x
**Ballon (2012)**[84]	B																														x
	T																														x

Study name (year)	Osteosynthesis system	Plate breakage (%)	Screw breakage (%)	Operation time (mean±SD, in minutes)[a]	Handling by surgeon (0: worst; 10: excellent)	Infection (%)	Swelling (%)	Abscess (%)	Pain (%)	MMO (%)	Dehiscence (%)	Plate exposure (%)	Malunion (%)	Mobility segments (%)	Malocclusion (%)
		Perioperative endpoints				Short-term follow-up							Intermediate follow-up		
**Paeng (2012)**[6]	B		1.6[#]			NNA									
	T		0[#]			NNA									
**Ueki (2012)**[85]	B					0					0			0	
	T					0					0			0	
**Blakey (2014)**[86]	B														
	T														
**Lee (2014)**[87]	B					0									
	T					0									
**Ueki (2015)**[88]	B					0					0	0[c]	0	0	
	T					0					0	0[c]	0	0	

Study name (year)	Osteosynthesis system	Pain (%)	MMO (mean±SD)	TMJ-dysfunction (%)	Malocclusion (%)	Pain (%)	MMO (mean±SD)	TMJ-dysfunction (%)	MFIQ (median, IQR)	Abscess (%)	Swelling (%)	Palpability screw/plate (%)	Satisfaction (%)	Symptomatic plate/screw removal (%)	Costs (direct and indirect; mean±SD)[a]	Revision surgery (not device removal; %)	Skeletal stability assessed (yes)
		Intermediate follow-up (cont.)				Long-term follow-up							Overall follow-up				
**Paeng (2012)**[6]	B															NNA	x
	T															NNA	x
**Ueki (2012)**[85]	B				0												x
	T				0												x
**Blakey (2014)**[86]	B															0	x
	T															0	x
**Lee (2014)**[87]	B																x
	T																x
**Ueki (2015)**[88]	B				0												
	T				0												

All data are given in percentages, unless stated otherwise. All unit of analysis was number of patients, unless stated otherwise. [*]Unit of analysis was plates. [#]Unit of analysis was screws.[†]Data of the follow-up moment 6-12 months given. The follow-up moment 12-24 months had high proportion of participants lost to follow-up. [a]Data given in mean±standard deviation. [b]Percentage of surgeons 'satisfied' or higher with the osteosynthesis system. [c]If no wound dehiscence was present, plate exposure was also assessed as not present. [d]Only the difference in mean operative time of biodegradable compared to titanium osteosyntheses was reported. [e]Two follow-up moments: 1- and 2-year follow-up, respectively. [f]Postoperative minus preoperative MMO. B, biodegradable; T, titanium; O, objectively assessed; S, subjectively assessed; NNA, numbers not available. MMO, maximal mouth opening; TMJ-dysfunction, temporomandibular joint dysfunction; MFIQ, Mandibular Function Impairment Questionnaire; x, assessed (see Table S6). Empty cells: not reported. Note that (i) malocclusion and (ii)

analgesic usage after short-term follow-up are not mentioned in this table as these endpoints were not assessed in any of the included studies, and (iii) that certain continuous variables are shown without standard deviations because these were not reported in the original manuscripts.

Table S6: Operative displacement and relapse.

Study (first author, year)	Type of displacement	Operative displacement (mean±SD or median (IQR), in mm or ° angle)		Relapse (mean±SD or median (IQR), in mm or ° angle)		Follow-up	Lateral cephalometric reference marks
		**B**	**T**	**B**	**T**		
Randomised controlled trials							
**Matthews et al. (2003)**[1]	Mandibular horizontal	4.0 (0.0-5.0)	4.0 (3.0-8.0)	NNA		1 yr	Go
	Mandibular vertical	4.5 (2.0-6.0)	4.5 (1.5-5.0)				Go
	Mandibular angle	4.2 (2.2-8.8)	1.5 (1.0-8.0)				Ar-Go-Gn
**Norholt et al. (2004)**[62]	Maxillary horizontal	2.3±2.8	2.4±2.2	0.03±0.82	0.24±1.4	6 wks	AI
	Maxillary vertical	2.1±1.6	2.2±2.2	0.58±0.60	0.56±1.9		AI
	Maxillary angle	NR	NR	3.6±2.2	0.67±2.6		OP
**Ueki et al. (2005)**[64]	Mandibular horizontal	2.0±3.6[a]	3.5±5.3[a]	2.4±2.89	0.7±4.99	1 yr	Pg
	Mandibular vertical	2.9±2.6[a]	4.3±3.3[a]	0.1±2.61	2.5±2.94		Pg
	Mandibular angle	8.8±2.0[a]	3.4±2.4[a]	6.7±2.53	2.6±2.33		Ar-Go-Gn
**Cheung et al. (2008)**[2]	Maxillary horizontal (adv)	3.43±2.03	4.0±2.45	NNA		1 yr	Point-A
	Maxillary horizontal (setb)	1.12±1.12	1.59±0.84	NNA			
	Maxillary vertical (imp)	2.65±2.05	3.27±1.84	NNA			
	Maxillary vertical (elong)	2.87±1.94	1.45±1.1	NNA			
**Park et al. (2010)**[65]	Maxillary horizontal	1.88±1.19	3.20±2.19	0.08±0.23	0.0±0.0	6 mos	ANS
	Maxillary vertical	1.32±1.66	2.25±1.98	0.07±0.41	0.0±0.24		ANS
	Maxillary angle	2.45±1.12	3.45±1.80	0.12±0.41	0.15±0.24		SNA
	Mandibular horizontal	8.18±5.24	8.70±8.43	0.60±1.96	1.45±2.95		Pg
	Mandibular vertical	1.05±2.89	2.00±2.16	1.26±1.52	1.85±1.73		Pg
	Mandibular angle	0.55±4.20	0.75±6.79	5.01±4.61	2.30±4.10		Ar-Go-Gn
**Tuovinen et al. (2010)**[4]	All	NNA	NNA	NNA	NNA		
Prospective cohort studies							

Study (first author, year)	Type of displacement	Operative displacement (mean±SD or median (IQR), in mm or ° angle)		Relapse (mean±SD or median (IQR), in mm or ° angle)		Follow-up	Lateral cephalometric reference marks
		**B**	**T**	**B**	**T**		
**Ferrretti et al. (2002)**[72]	Mandibular horizontal	5.67±1.70[b]	4.80±1.33[b]	0.83±1.25	0.25±1.38	6 mos	Point-B
**Dhol et al. (2008)**[73]	Maxillary horizontal	2.02±0.39[c]	2.45±0.57[c]	0.20±0.43[c]	0.80±0.43[c]	≥8 mos	Point-A
	Maxillary vertical	2.46±0.71[c]	2.14±0.65[c]	0.12±0.57[c]	0.64±0.57[c]		Point-A
	Maxillary angle	0.99±0.84[c]	2.36±0.74[c]	0.04±0.69[c]	0.00±0.69[c]		Palatal plane
**Bakelen et al. (2014)**[74]	Mandibular horizontal	3.2±1.6	4.2±2.2	0.03±1.7	0.3±2.3	2 yrs	Point-B
	Mandibular vertical	4.8±1.8	3.2±2.4	1.1±1.5	0.9±1.6		
Retrospective cohort studies							
**Harada et al. (1997)**[75]	Mandibular horizontal	NNA		1.62±1.28	1.05±1.00	1 yr	Pg
	Mandibular vertical			0.37±1.33	0.00±0.77		
**Costa et al. (2006)**[76]	Maxillary horizontal	3.50±1.65	3.54±1.54	0.90±1.37	0.16±0.72	1 yr	Point-A
	Maxillary vertical	0.95±1.79	2.33±1.83	1.55±1.36	0.042±1.31		Point-A
	Maxillary angle	3.00±1.28	3.32±1.62	0.57±1.20	0.02±0.64		SNA
**Landes et al. (2006)**[5],[d]	Maxillary horizontal (adv)	3.5±4.1	5.4±3.5	2.3±1.8	2.4±2.0	1 yr	Point-A
	Maxillary horizontal (setb)	2.8±3.7	1.9±1.8	2.3±1.9	2.5±1.7		Point-A
	Maxillary vertical (imp)	1.9±1.7	3.3±2.7	2.1±1.9	2.2±1.5		ANS
	Maxillary vertical (elong)	4.2±3.6	3.7±5.2	3.8±3.1	3.1±3.6		ANS
	Mandibular horizontal (adv)	4.6±3.6	6.3±8.8	4.9±4.3	5.1±8.2		Point-B
	Mandibular horizontal (setb)	7.5±8.3	7.2±3.2	3.0±2.0	1.7±2.0		Point-B
	Mandibular angle (CW)	11.8±9.9	7.9±6.6	6.7±8.9	8.2±9.6		Ar-Go-Gn
	Mandibular angle (CCW)	4.5±3.2	6.3±6.6	6.8±5.2	4.2±5.9		Ar-Go-Gn

Study (first author, year)	Type of displacement	Operative displacement (mean±SD or median (IQR), in mm or ° angle)		Relapse (mean±SD or median (IQR), in mm or ° angle)		Follow-up	Lateral cephalometric reference marks
		B	T	B	T		
**Turvey et al. (2006)**[77]	Mandibular horizontal	5.20±2.37	4.96±2.60	0.54±3.25	0.33±2.2	1 yr	Point-B
	Mandibular vertical	4.34±1.68	4.01±2.30	1.36±2.59	1.15±1.80		
**Ueki et al. (2006a and b)**[78]	Maxillary horizontal (a)	2.9±2.3[a]	3.1±2.9[a]	1.30±2.14	0.90±2.86	1 yr	Point-A
	Maxillary vertical (a)	0.8±2.0[a]	0.4±1.8[a]	2.30±2.10	0.40±1.90		Point-A
	Maxillary angle (a)	2.7±1.4[a]	3.1±1.6[a]	0.20±1.31	0.80±1.43		SNA
	Maxillary horizontal (b)	1.7±2.7[a]	2.8±1.7[a]	2.00±2.27	1.10±2.04		Point-A
	Maxillary vertical (b)	3.7±2.0[a]	0.0±1.2[a]	3.30±2.15	2.00±1.39		Point-A
	Maxillary angle (b)	2.5±2.3[a]	2.6±1.5[a]	0.40±1.92	1.10±1.61		SNA
**Landes et al. (2007)**[79],[d]	Maxillary horizontal (adv)	2.5±1.0	5.4±3.5	1.2±0.8	2.4±2.0	1yr	Point-A
	Maxillary horizontal (setb)	2.2±2.4	1.9±1.8	1.8±1.9	2.5±1.7		Point-A
	Maxillary vertical (imp)	1.0±0.7	3.3±2.7	1.1±1.1	2.2±1.5		ANS
	Maxillary vertical (elong)	6.5±3.4	3.7±5.2	2.0±1.4	3.1±3.6		ANS
	Mandibular horizontal (adv)	5.5±3.7	6.3±8.8	2.6±2.7	5.1±8.2		Point-B
	Mandibular horizontal (setb)	11.2±7.7	7.2±3.2	2.7±2.6	1.7±2.0		Point-B
	Mandibular angle (CW)	7.9±2.4	7.9±6.6	2.4±2.7	8.2±9.6		Ar-Go-Gn
	Mandibular angle (CCW)	6.9±2.6	6.3±6.6	7.0±5.4	4.2±5.9		Ar-Go-Gn
**Choi et al. (2010)**[82]	Mandibular horizontal (setb)	7.11±2.7	5.69±1.10	1.94±0.93	1.60±0.58	14.5 mos	Point-B
	Mandibular vertical (setb)	1.58±2.73	1.81±1.44	0.08±1.38	0.10±1.10		Point-B
**Ueki et al. (2011)**[83]	Mandibular horizontal	5.0±7.2[e]	5.1±6.9	NNA		1 yr	Pg

Study (first author, year)	Type of displacement	Operative displacement (mean±SD or median (IQR), in mm or ° angle)		Relapse (mean±SD or median (IQR), in mm or ° angle)		Follow-up	Lateral cephalometric reference marks
		B	T	B	T		
	Mandibular vertical	1.5±4.0[e]	3.7±5.5				Pg
	Mandibular angle	3.2±5.4[e]	2.4±3.2				Ar-Go-Gn
**Ballon et al. (2012)**[84]	Maxillary horizontal (adv)	2.70±1.94	4.28±2.37	1.84±1.69	1.59±1.48	≥6 mos	Point-A
	Maxillary horizontal (setb)	3.46±2.63	3.70±2.10	2.02±1.89	1.70±1.64		Point-A
	Maxillary vertical (imp)	3.13±2.25	3.25±1.55	2.67±2.08	1.40±1.42		ANS
	Maxillary vertical (elong)	5.22±4.05	2.92±2.64	2.68±2.65	1.39±1.55		ANS
	Mandibular horizontal (adv)	4.89±3.67	4.09±2.84	3.65±3.39	2.09±1.43		Point-B
	Mandibular horizontal (setb)	9.31±5.46	8.55±4.85	4.86±2.87	1.05±1.31		Point-B
	Mandibular angle (CW)	7.75±6.18	9.57±7.13	4.55±3.52	10.63±9.47		Ar-Go-Gn
	Mandibular angle (CCW)	4.79±3.09	6.50±6.06	6.36±4.86	5.00±6.53		Ar-Go-Gn
**Paeng et al. (2012)**[6]	Mandibular horizontal	6.7±2.2	7.0±3.2	0.51±1.23	0.75±1.85	6 mos	Point-B
	Mandibular vertical	NNA		0.71±1.35	1.5±1.39		Me
**Ueki et al. (2012)**[85]	Maxillary horizontal	2.4±2.1[e]	2.7±2.6	0.30±1.25	0.50±1.99	1 yr	Point-A
	Maxillary vertical	1.0±4.3[e]	0.8±2.9	1.00±1.36	1.20±1.45		Point-A
	Maxillary angle	1.2±3.6[e]	2.2±2.9	0.95±0.76	0.60±1.22		SNA
**Blakey et al. (2014)**[86]	Maxillary horizontal	5.61±1.30	7.07±2.30	2.06±1.91	1.34±1.34	1 yr	Point-A
**Lee et al. (2014)**[87]	Mandibular horizontal	13.97±1.39	9.59±1.51	1.89±1.33	3.02±1.05	6 mos	Pg
	Mandibular vertical	1.7±1.68	0.65±1.6	0.83±0.53	1.51±1.56		Pg
	Mandibular angle	3.57±1.06	2.81±0.65	0.33±0.85	1.39±0.48		Ar-Pg to FH

Perioperative displacement and relapse are given in absolute values. The direction of operative displacement (e.g., setback or advancement) are only stated in this table whenever this was explicitly stated in the original manuscript. [a]Time interval values of cephalometric data (e.g., 1-year postoperative minus immediate postoperative data) were calculated based on the cephalometric data of specific time points (e.g., 1 year and immediate postoperative data), assuming normal distribution of data. [b]Discrepancy

exists between data in the text and tables of the original manuscript. The authors did not respond to contact attempts. Data presented in the text of the original manuscript were used. [c]Data presented as mean ± standard error of the mean (SEM). [d]Landes et al. (2006) and Landes et al. (2007) have included the identical control groups. The means and standard deviations of both the intervention groups were pooled and analyzed as a single pair-wise comparison with that specific control group, assuming normal distribution of data. [e]The two subgroups of biodegradable osteosyntheses (i.e., uHA/PLLA and PLLA subgroups) were pooled and analyzed as a single pair-wise comparison between biodegradable and titanium osteosyntheses. a: subgroup Le Fort advancement + BSSO setback. b: subgroup Le Fort I advancement + IVRO without fixation. IQR, interquartile range; NNA, numbers not available; Go, gonion; Ar-Go-Gn, articular-gonion-gnathion angle (gonial angle); AI, anterior implant; NS, nasion-sella line; NSP, nasion-sella perpendicular line; OP, occlusal plane; Pg, pogonion; SNA, sella-nasion-A point angle; adv, advancement; setb, setback; imp, impaction; elong, elongation; ANS, anterior nasal spine; CW, clockwise rotation; CCW, counter-clockwise rotation; Me, menton; Ar-Pg, articular-pogonion; FH, Frankfurt horizontal plane; wks, weeks; mos, months; yrs, years.

Table S7: Results of univariable meta-regression analyses to analyze the effect of risk of bias items on the log risk ratio of symptomatic device removal using a random effects model.

Risk of bias item	Regression coefficient	95% CI (lower to upper border)	P-value
**Domain 1 (ref. = low RoB)**	0.64	-0.15 to 1.43	0.11
Some concerns	-0.56	-1.74 to 0.61	0.35
High RoB	-2.23	-5.40 to 0.94	0.35
**Domain 2 (ref. = low RoB)**	-0.06	-0.91 to 0.79	0.89
High RoB	0.70	-0.50 to 1.90	0.25
**Domain 3 (ref. = low RoB)**	-0.31	-1.68 to 1.06	0.65
Some concerns	0.34	-2.00 to 2.69	0.78
High RoB	0.80	-1.87 to 3.48	0.56
**Domain 4 (ref. = low RoB)**	-0.46	-1.71 to 0.79	0.47
Some concerns	1.15	-1.40 to 3.70	0.38
High RoB	0.95	-1.63 to 3.54	0.47
**Domain 5 (ref. = low RoB)**	0.42	-0.11 to 0.96	0.12
Some concerns	-2.01	-5.10 to 1.07	0.20
**Overall RoB (ref. = some concerns)**	-0.18	-1.29 to 0.93	0.75
High RoB	0.64	-0.71 to 2.00	0.35

RoB, Risk of Bias. Ref., reference item. 95% CI, 95% confidence interval. The meta-regression analysis shows none of the individual risk of bias items have a significant effect on the symptomatic device removal rate.

Table S8: Input and results of the trial sequential analyses using the random-effects (DerSimonian-Laird) model with the corresponding interpretations.

Endpoint	Control event proportion (titanium)[a]	Relative risk (95% CI)[a]	Diversity (D^2) [a]	Total N/RIS	Crossed conventional test boundary	Crossed O'Brien-Fleming boundary	Crossed futility boundary	Interpretation
				Short-term follow-up				
**Infection**	4.3%	1.03 (0.46; 2.28)	0.0	645/780586[b]	Not estimable due to <5% of RIS achieved			Inconclusive, potentially false neutral
**Swelling**	13.2%	1.51 (0.68; 3.38)	0.62	255/2536	No	No	No	Inconclusive, potentially false neutral
**Dehiscence**	2.3%	1.53 (0.52; 4.50)	0.0	421/5865	No	No	No	Inconclusive, potentially false neutral
				Intermediate follow-up				
**Mobility bone segments**	10.3%	1.37 (0.47; 3.99)	0.0	155/2302	No	No	No	Inconclusive, potentially false neutral
				Long-term follow-up				
**Malocclusion**	11.2%	0.93 (0.39; 2.26)	0.0	217/49794[b]	Not estimable due to <5% of RIS achieved			Inconclusive, potentially false neutral
**Swelling**	2.0%	2.42 (0.52; 11.19)	0.0	178/1316	No	No	No	Inconclusive, potentially false neutral
**Palpability of screws/plates**	23.2%	0.38 (0.11; 1.28)	0.67	400/619	No	No	No	Inconclusive, potentially false neutral

				Overall follow-up				
**Symptomatic device removal**	8.3%	1.29 (0.68; 2.44)	0.52	777/9717	No	No	No	Inconclusive, potentially false neutral
**Revision surgery (not device removal)**	2.0%	1.40 (0.37; 5.34)	0.0	377/11445	No	No	No	Inconclusive, potentially false neutral

RIS, required information size. [a]According to the observed relative risk and diversity of the present meta-analysis including randomised controlled trials only. [b]RIS is very high due to a very small relative risk reduction. Outcomes that are not mentioned were assessed in no or a single randomised controlled trials, or were only assessed in total zero-event trials.

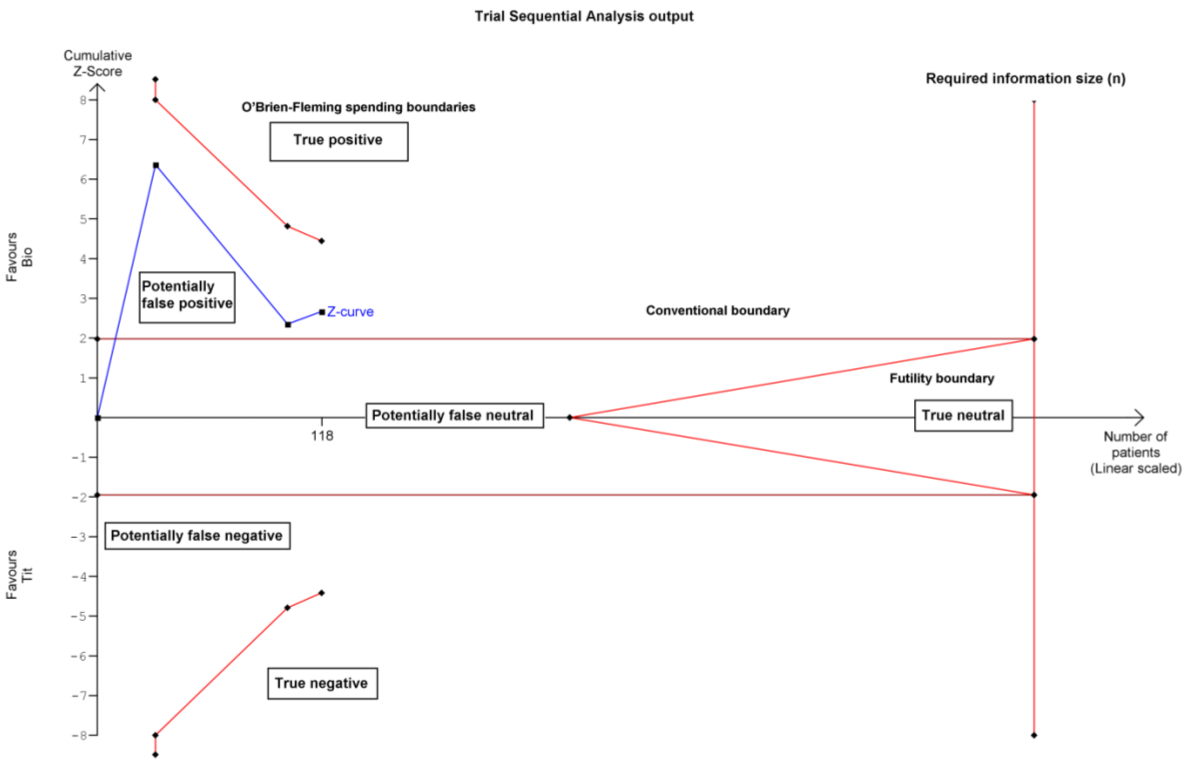

Figure S1: Example graph with explanation of the trial sequential analysis. The y-axis represents the cumulative Z-score and the x-axis the number of patients of included trials. A Z-score of ±1.96 corresponds to α = 0.05 (conventional boundaries). The required information size is the number of patients needed to draw a definite conclusion and this number is comparable to a sample size calculation in randomised controlled trials. The O'Brien-Fleming spending boundaries are trial sequential adjusted boundaries; the fewer patients are randomised, the wider these borders are due to increased chance of random errors. Crossing the futility boundary indicates that the intervention is unlikely to have the anticipated effect. The interpretation of each area is presented as textboxes in the graph. Thus, TSA provides three borders: conventional test boundaries (α = 0.05; Z = +/- 1.96; i.e., crossing boundary means potentially false positive or negative), O'Brien-Fleming spending boundaries (i.e., crossing boundary means true positive or negative effect), and futility boundaries (crossing boundary means true neutral effect). If no boundaries are crossed, the evidence remain inconclusive (i.e., potentially false neutral).

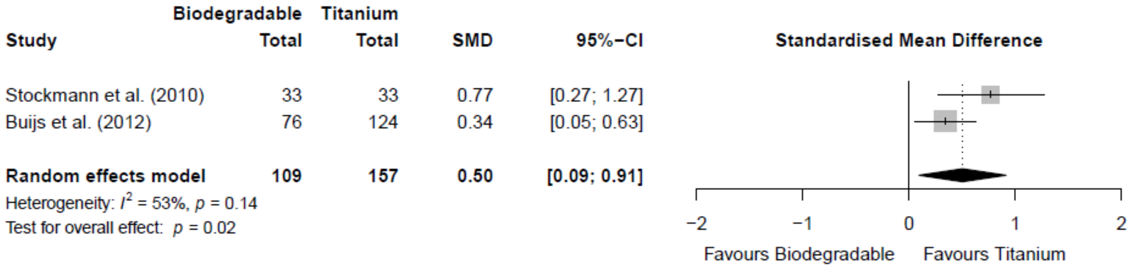

Figure S2: Forest plot of the endpoint operative time in minutes. *SMD, standardised mean difference; 95%-CI, 95% confidence interval.*

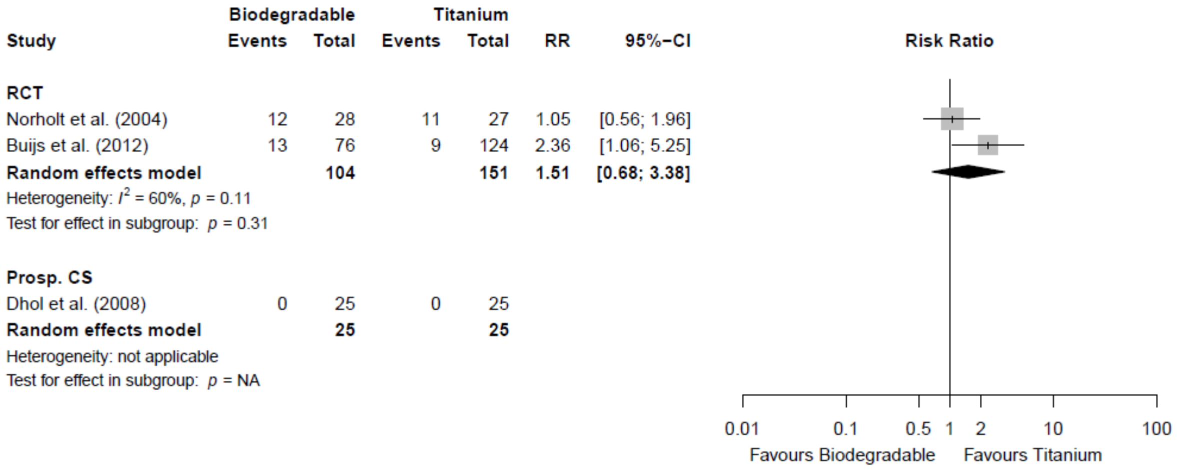

Figure S3: Forest plot of the endpoint swelling (<4 weeks follow-up) stratified by study design. *RCT, randomised controlled trials; Prosp. CS, prospective cohort studies; RR, risk ratio; 95%-CI, 95% confidence interval.*

Study	Biodegradable Events	Biodegradable Total	Titanium Events	Titanium Total	RR	95%-CI
**RCT**						
Cheung et al. (2004)	2	30	3	30	0.67	[0.12; 3.71]
Norholt et al. (2004)	1	28	0	27	2.89	[0.12; 68.06]
Ueki et al. (2005)	0	20	0	20		
Stockmann et al. (2010)	1	33	0	33	3.00	[0.13; 71.04]
Buijs et al. (2012)	3	76	2	124	2.45	[0.42; 14.31]
**Random effects model**		**187**		**234**	**1.53**	**[0.52; 4.50]**
Heterogeneity: $I^2 = 0\%$, $p = 0.68$						
Test for effect in subgroup: $p = 0.44$						
**Retrosp. CS**						
Costa et al. (2006)	0	10	0	12		
Ueki et al. (2006a)	0	12	0	12		
Ueki et al. (2006b)	0	9	0	14		
Ueki et al. (2009)	0	11	0	12		
Ueki et al. (2012)	0	40	0	20		
Ueki et al. (2015)	0	61	0	13		
**Random effects model**		**143**		**83**		
Heterogeneity: not applicable						
Test for effect in subgroup: p = NA						

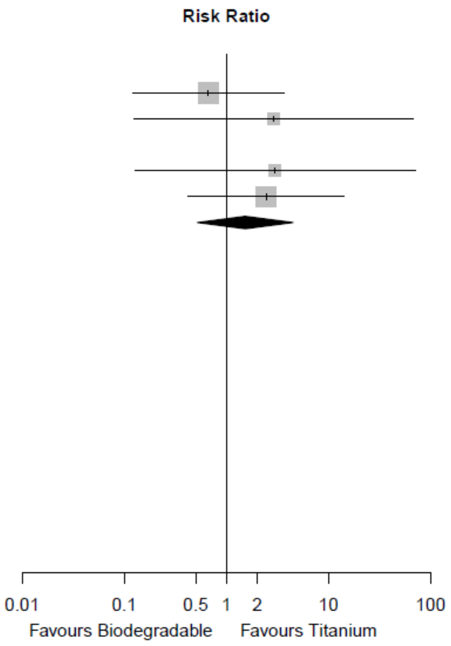

Figure S4: Forest plot of the endpoint dehiscence (<4 weeks follow-up) stratified by study design. *RCT, randomised controlled trials; Retrosp. CS, retrospective cohort studies; RR, risk ratio; 95%-CI, 95% confidence interval.*

Study	Biodegradable Mean	Biodegradable SD	Biodegradable Total	Titanium Mean	Titanium SD	Titanium Total	SMD	95%-CI
**RCT**								
Cheung et al. (2004)	1.20	1.35	30	1.47	1.50	30	−0.19	[−0.69; 0.32]
Buijs et al. (2012)	5.15	8.90	76	4.70	10.60	124	0.04	[−0.24; 0.33]
**Random effects model**			**106**			**154**	**−0.01**	**[−0.26; 0.24]**
Heterogeneity: $I^2 = 0\%$, $p = 0.44$								
Test for effect in subgroup: $p = 0.93$								

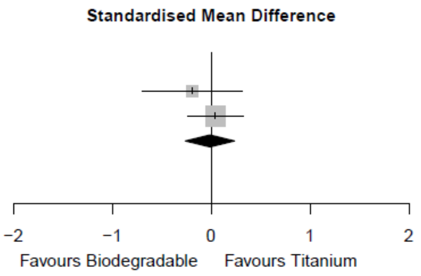

Figure S5: Forest plot of the endpoint pain (6-12 weeks follow-up) stratified by study design. *RCT, randomised controlled trials; SMD, standardised mean difference; 95%-CI, 95% confidence interval.*

Study	Biodegradable Mean	SD	Total	Titanium Mean	SD	Total	SMD	95%-CI
**RCT**								
Cheung et al. (2004)	0.46	1.10	24	0.67	1.13	24	−0.19	[−0.75; 0.38]
Ueki et al. (2005)	0.80	1.80	20	1.00	2.10	20	−0.10	[−0.72; 0.52]
Gareb et al. (2017)	6.96	18.68	51	5.68	17.55	81	0.07	[−0.28; 0.42]
**Random effects model**			**95**			**125**	**−0.02**	**[−0.29; 0.25]**

Heterogeneity: $I^2 = 0\%$, $p = 0.72$
Test for effect in subgroup: $p = 0.89$

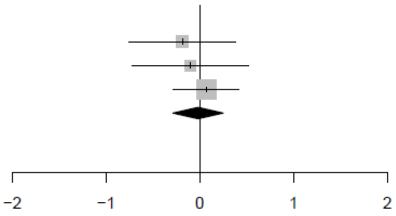

Figure S6: Forest plot of the endpoint pain (>12 weeks follow-up) stratified by study design. *RCT, randomised controlled trials; SMD, standardised mean difference; 95%-CI, 95% confidence interval.*

Study	Biodegradable Mean	SD	Total	Titanium Mean	SD	Total	SMD	95%-CI
**RCT**								
Ueki et al. (2005)	3.40	2.26	20	5.60	1.86	20	−1.04	[−1.71; −0.38]
Yu et al. (2014)	0.31	6.80	50	1.86	7.51	51	−0.21	[−0.61; 0.18]
**Random effects model**			**70**			**71**	**−0.58**	**[−1.39; 0.22]**

Heterogeneity: $I^2 = 77\%$, $p = 0.04$
Test for effect in subgroup: $p = 0.16$

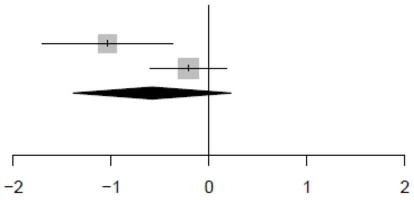

Figure S7: Forest plot of the endpoint maximum mouth opening (>12 weeks follow-up) stratified by study design. *RCT, randomised controlled trials; SMD, standardised mean difference; 95%-CI, 95% confidence interval.*

Study	Biodegradable Events	Biodegradable Total	Titanium Events	Titanium Total	RR	95%-CI
**Maxillary osteotomy only**						
Norholt et al. (2004)	0	25	3	21	0.12	[0.01; 2.20]
Gareb et al. (2017)	0	8	0	8		
**Random effects model**		**33**		**29**	**0.12**	**[0.01; 2.20]**
Heterogeneity: not applicable						
Test for effect in subgroup: p = 0.15						
**Mandibular osteotomy only**						
Stockmann et al. (2010)	0	33	1	33	0.33	[0.01; 7.89]
Yoshioka et al. (2012)	6	110	3	90	1.64	[0.42; 6.36]
Yu et al. (2014)	0	50	2	51	0.20	[0.01; 4.14]
Gareb et al. (2017)	19	55	14	87	2.15	[1.18; 3.92]
**Random effects model**		**248**		**261**	**1.60**	**[0.76; 3.34]**
Heterogeneity: I^2 = 16%, p = 0.31						
Test for effect in subgroup: p = 0.21						
**Bimaxillary osteotomy**						
Cheung et al. (2004)	6	30	3	30	2.00	[0.55; 7.27]
Tuovinen et al. (2010)	1	51	2	50	0.49	[0.05; 5.24]
Gareb et al. (2017)	4	16	5	29	1.45	[0.45; 4.65]
**Random effects model**		**97**		**109**	**1.45**	**[0.64; 3.27]**
Heterogeneity: I^2 = 0%, p = 0.59						
Test for effect in subgroup: p = 0.37						
Heterogeneity: I^2 = 14%, p = 0.32						
Test for subgroup differences: χ^2_2 = 2.87, df = 2 (p = 0.24)						

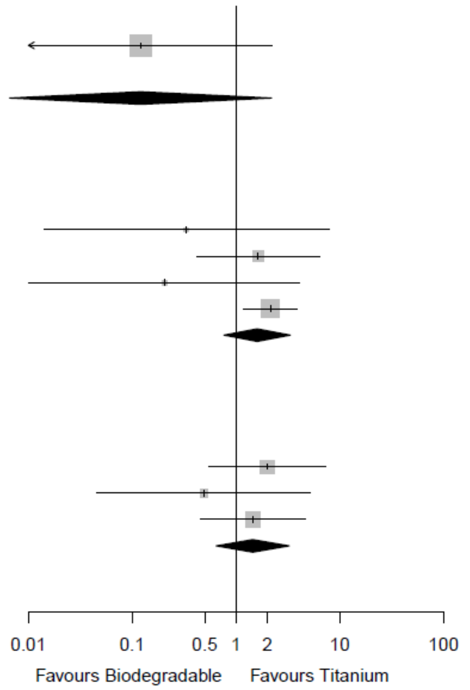

Figure S8: Forest plot of the endpoint symptomatic device removal (overall follow-up) of studies stratified maxillary, mandibular, and bimaxillary osteotomies. *RR, risk ratio; 95%-CI, 95% confidence interval.*

Study	Biodegradable Events	Biodegradable Total	Titanium Events	Titanium Total	RR	95%-CI
**Screws only**						
Stockmann et al. (2010)	0	33	1	33	0.33	[0.01; 7.89]
Yu et al. (2014)	0	50	2	51	0.20	[0.01; 4.14]
**Random effects model**		**83**		**84**	**0.26**	**[0.03; 2.28]**
Heterogeneity: I^2 = 0%, p = 0.83						
Test for effect in subgroup: p = 0.22						
**Plates and screws**						
Yoshioka et al. (2012)	6	110	3	90	1.64	[0.42; 6.36]
Gareb et al. (2017)	23	79	19	124	1.90	[1.11; 3.25]
**Random effects model**		**189**		**214**	**1.86**	**[1.13; 3.07]**
Heterogeneity: I^2 = 0%, p = 0.84						
Test for effect in subgroup: p = 0.01						
Heterogeneity: I^2 = 6%, p = 0.36						
Test for subgroup differences: χ^2_1 = 3.00, df = 1 (p = 0.08)						

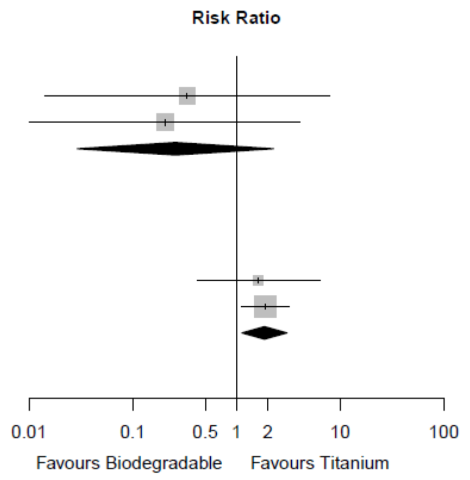

Figure S9: Forest plot of the endpoint symptomatic device removal (overall follow-up) of studies including osteosynthesis by plates and screws versus only screws. *RR, risk ratio; 95%-CI, 95% confidence interval.*

Study	Biodegradable Events	Biodegradable Total	Titanium Events	Titanium Total	RR	95%-CI
**Follow-up = up to 1yr**						
Norholt et al. (2004)	0	25	3	21	0.12	[0.01; 2.20]
Yu et al. (2014)	0	50	2	51	0.20	[0.01; 4.14]
**Random effects model**		**75**		**72**	**0.16**	**[0.02; 1.26]**
Heterogeneity: I^2 = 0%, p = 0.81						
Test for effect in subgroup: p = 0.08						
**Follow-up = >1yr**						
Cheung et al. (2004)	6	30	3	30	2.00	[0.55; 7.27]
Stockmann et al. (2010)	0	33	1	33	0.33	[0.01; 7.89]
Tuovinen et al. (2010)	1	51	2	50	0.49	[0.05; 5.24]
Yoshioka et al. (2012)	6	110	3	90	1.64	[0.42; 6.36]
Gareb et al. (2017)	23	79	19	124	1.90	[1.11; 3.25]
**Random effects model**		**303**		**327**	**1.73**	**[1.10; 2.72]**
Heterogeneity: I^2 = 0%, p = 0.68						
Test for effect in subgroup: p = 0.02						
Heterogeneity: I^2 = 21%, p = 0.27						
Test for subgroup differences: χ^2_1 = 4.87, df = 1 (p = 0.03)						

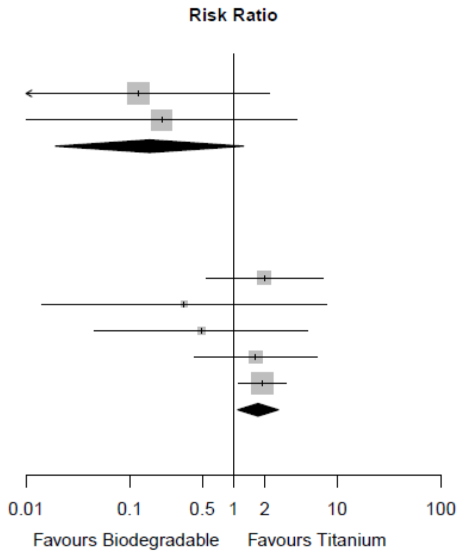

Figure S10: Forest plot of the endpoint symptomatic device removal (overall follow-up) stratified by ≤1-year and >1-year follow-up. *RR, risk ratio; 95%-CI, 95% confidence interval.*

Study	Biodegradable Mean	Biodegradable SD	Biodegradable Total	Titanium Mean	Titanium SD	Titanium Total	SMD	95%-CI
**RCT**								
Norholt et al. (2004)	0.03	0.82	27	0.24	1.40	28	−0.18	[−0.71; 0.35]
Park et al. (2010)	0.08	0.23	30	0.00	0.00	10		
**Random effects model**			**57**			**38**	**−0.18**	**[−0.71; 0.35]**
Heterogeneity: not applicable								
Test for effect in subgroup: p = 0.51								
**Retrosp. CS**								
Costa et al. (2006)	0.90	1.37	10	0.16	0.72	12	0.67	[−0.20; 1.54]
Ueki et al. (2006a)	1.30	2.14	12	0.90	2.86	12	0.15	[−0.65; 0.95]
Ueki et al. (2006b)	2.00	2.27	9	1.10	2.04	14	0.41	[−0.44; 1.25]
Landes et al. (2006 & 2007)	2.00	1.65	26	2.40	2.00	21	−0.22	[−0.79; 0.36]
Ballon et al. (2012)	1.84	1.69	26	1.59	1.48	29	0.16	[−0.37; 0.69]
Ueki et al. (2012)	0.30	1.25	40	0.50	1.99	20	−0.13	[−0.67; 0.41]
Blakey et al. (2014)	2.06	1.91	27	1.34	1.34	30	0.43	[−0.09; 0.96]
**Random effects model**			**150**			**138**	**0.15**	**[−0.08; 0.39]**
Heterogeneity: I^2 = 0%, p = 0.49								
Test for effect in subgroup: p = 0.21								
Heterogeneity: I^2 = 0%, p = 0.46								
Test for subgroup differences: χ^2_1 = 1.25, df = 1 (p = 0.26)								

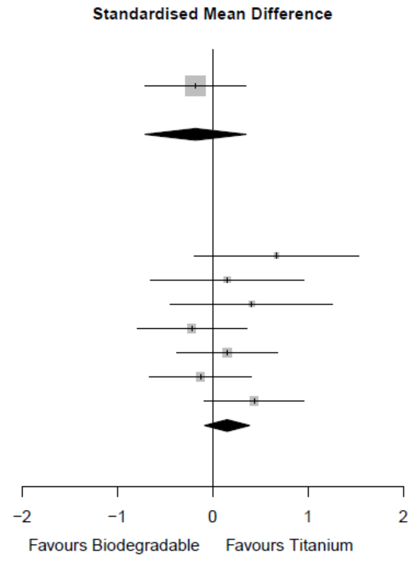

Figure S11: Forest plot of the endpoint maxillary horizontal relapse after maxillary advancement stratified by study design (overall follow-up). *RCT, randomised controlled trials; Retrosp. CS, retrospective cohort studies; SMD, standardised mean difference; 95%-CI, 95% confidence interval.*

Study	Biodegradable Mean	SD	Total	Titanium Mean	SD	Total	SMD	95%-CI
**Retrosp. CS**								
Costa et al. (2006)	0.57	1.20	10	0.02	0.64	12	0.57	[−0.29; 1.43]
Ueki et al. (2006a)	0.20	1.31	12	0.80	1.43	12	−0.42	[−1.23; 0.39]
Ueki et al. (2006b)	0.40	1.92	9	1.10	1.61	14	−0.39	[−1.24; 0.46]
Ueki et al. (2012)	0.95	0.76	40	0.60	1.22	20	0.37	[−0.17; 0.91]
**Random effects model**			**71**			**58**	**0.07**	**[−0.41; 0.55]**
Heterogeneity: $I^2 = 39\%$, $p = 0.18$								
Test for effect in subgroup: $p = 0.78$								

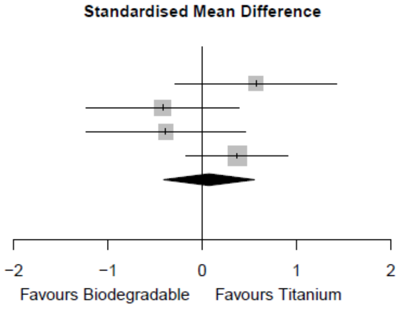

Figure S12: Forest plot of the endpoint maxillary angular relapse after maxillary advancement stratified by study design (overall follow-up). *Retrosp. CS, retrospective cohort studies; SMD, standardised mean difference; 95%-CI, 95% confidence interval.*

Study	Biodegradable Mean	SD	Total	Titanium Mean	SD	Total	SMD	95%-CI
**Retrosp. CS**								
Landes et al. (2006 & 2007)	2.08	1.85	16	2.50	1.70	8	−0.22	[−1.08; 0.63]
Ballon et al. (2012)	2.02	1.89	13	1.70	1.64	10	0.17	[−0.65; 1.00]
**Random effects model**			**29**			**18**	**−0.02**	**[−0.61; 0.57]**
Heterogeneity: $I^2 = 0\%$, $p = 0.51$								
Test for effect in subgroup: $p = 0.95$								

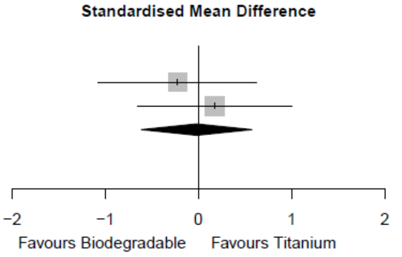

Figure S13: Forest plot of the endpoint maxillary horizontal relapse after maxillary setback stratified by study design (overall follow-up). *Retrosp. CS, retrospective cohort studies; SMD, standardised mean difference; 95%-CI, 95% confidence interval.*

Study	Biodegradable Mean	SD	Total	Titanium Mean	SD	Total	SMD	95%-CI
**RCT**								
Norholt et al. (2004)	0.58	0.60	27	0.56	1.90	28	0.01	[−0.51; 0.54]
Park et al. (2010)	0.07	0.41	30	0.00	0.24	10	0.18	[−0.53; 0.90]
**Random effects model**			**57**			**38**	**0.07**	**[−0.35; 0.50]**
Heterogeneity: $I^2 = 0\%$, $p = 0.71$								
Test for effect in subgroup: $p = 0.74$								
**Retrosp. CS**								
Ueki et al. (2006a)	2.30	2.10	12	0.40	1.90	12	0.92	[0.07; 1.76]
Ueki et al. (2006b)	3.30	2.15	9	2.00	1.39	14	0.73	[−0.14; 1.60]
Landes et al. (2006 & 2007)	1.81	1.73	17	2.20	1.50	13	−0.23	[−0.96; 0.49]
Ballon et al. (2012)	2.67	2.08	18	1.40	1.42	20	0.71	[0.05; 1.36]
Ueki et al. (2012)	1.00	1.36	40	1.20	1.45	20	−0.14	[−0.68; 0.40]
**Random effects model**			**96**			**79**	**0.35**	**[−0.13; 0.83]**
Heterogeneity: $I^2 = 56\%$, $p = 0.06$								
Test for effect in subgroup: $p = 0.16$								
Heterogeneity: $I^2 = 39\%$, $p = 0.13$								
Test for subgroup differences: $\chi^2_1 = 0.70$, df = 1 ($p = 0.40$)								

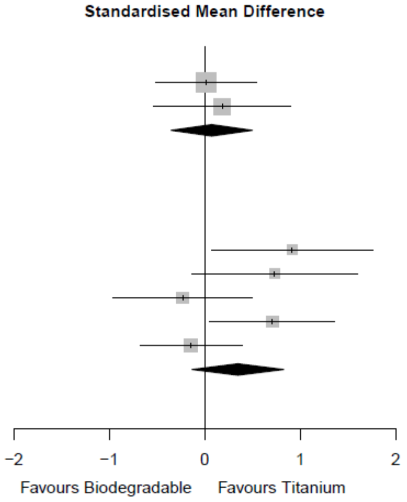

Figure S14: Forest plot of the endpoint maxillary vertical relapse after maxillary impaction stratified by study design (overall follow-up). *RCT, randomised controlled trials; Retrosp. CS, retrospective cohort studies; SMD, standardised mean difference; 95%-CI, 95% confidence interval.*

Study	Biodegradable Mean	SD	Total	Titanium Mean	SD	Total	SMD	95%-CI
**Retrosp. CS**								
Landes et al. (2006 & 2007)	3.20	2.76	27	3.10	3.60	14	0.03	[−0.61; 0.68]
Ballon et al. (2012)	2.68	2.65	21	1.39	1.55	19	0.58	[−0.06; 1.21]
**Random effects model**			**48**			**33**	**0.31**	**[−0.23; 0.84]**
Heterogeneity: $I^2 = 28\%$, $p = 0.24$								
Test for effect in subgroup: $p = 0.26$								

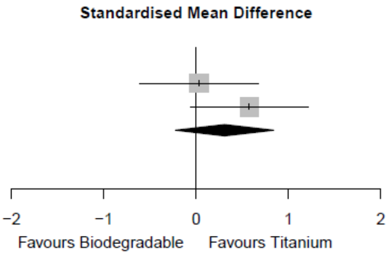

Figure S15: Forest plot of the endpoint maxillary vertical relapse after maxillary elongation stratified by study design (overall follow-up). *Retrosp. CS, retrospective cohort studies; SMD, standardised mean difference; 95%-CI, 95% confidence interval.*

Study	Biodegradable Mean	SD	Total	Titanium Mean	SD	Total	SMD	95%-CI
**Prosp. CS**								
Ferretti et al. (2002)	0.83	1.25	20	0.25	1.38	20	0.43	[−0.20; 1.06]
Bakelen et al. (2014)	0.03	1.70	15	0.30	2.30	22	−0.13	[−0.78; 0.53]
**Random effects model**			**35**			**42**	**0.16**	**[−0.39; 0.71]**
Heterogeneity: $I^2 = 31\%$, $p = 0.23$								
Test for effect in subgroup: $p = 0.56$								
**Retrosp. CS**								
Turvey et al. (2006)	0.54	3.25	34	0.33	2.20	35	0.08	[−0.40; 0.55]
Landes et al. (2006 & 2007)	4.24	3.96	14	5.10	8.20	18	−0.13	[−0.82; 0.57]
Ballon et al. (2012)	3.65	3.39	11	2.09	1.43	17	0.64	[−0.14; 1.41]
**Random effects model**			**59**			**70**	**0.14**	**[−0.23; 0.51]**
Heterogeneity: $I^2 = 8\%$, $p = 0.34$								
Test for effect in subgroup: $p = 0.45$								
Heterogeneity: $I^2 = 0\%$, $p = 0.46$								
Test for subgroup differences: $\chi^2_1 = 0.00$, df = 1 ($p = 0.95$)								

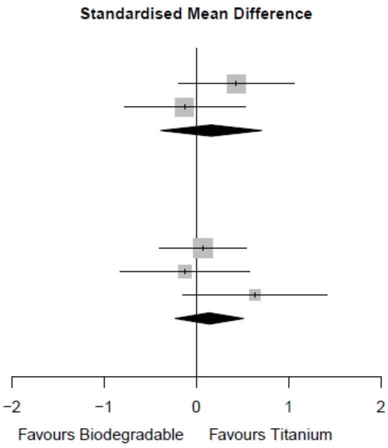

Figure S16: Forest plot of the endpoint mandibular horizontal relapse after mandibular advancement stratified by study design (overall follow-up). *Prosp. CS, prospective cohort studies; Retrosp. CS, retrospective cohort studies; SMD, standardised mean difference; 95%-CI, 95% confidence interval.*

Study	Biodegradable Mean	Biodegradable SD	Biodegradable Total	Titanium Mean	Titanium SD	Titanium Total	SMD	95%-CI
**RCT**								
Ueki et al. (2005)	2.40	2.89	20	0.70	4.99	20	0.41	[−0.22; 1.04]
Park et al. (2010)	0.60	1.96	30	1.45	2.95	10	−0.37	[−1.09; 0.35]
**Random effects model**			**50**			**30**	**0.04**	**[−0.73; 0.80]**
Heterogeneity: $I^2 = 61\%$, $p = 0.11$								
Test for effect in subgroup: $p = 0.92$								
**Retrosp. CS**								
Harada et al. (1997)	1.62	1.28	10	1.05	1.00	10	0.48	[−0.42; 1.37]
Landes et al. (2006 & 2007)	2.92	2.12	27	1.70	2.00	12	0.57	[−0.12; 1.27]
Choi et al. (2010)	1.94	0.93	15	1.60	0.58	15	0.43	[−0.30; 1.15]
Ballon et al. (2012)	4.86	2.87	27	1.05	1.31	11	1.47	[0.69; 2.25]
Paeng et al. (2012)	0.51	1.23	25	0.75	1.85	25	−0.15	[−0.71; 0.40]
Lee et al. (2014)	1.89	1.33	8	3.02	1.05	10	−0.91	[−1.90; 0.08]
**Random effects model**			**112**			**83**	**0.33**	**[−0.25; 0.91]**
Heterogeneity: $I^2 = 72\%$, $p < 0.01$								
Test for effect in subgroup: $p = 0.26$								
Heterogeneity: $I^2 = 67\%$, $p < 0.01$								
Test for subgroup differences: $\chi^2_1 = 0.36$, df = 1 ($p = 0.55$)								

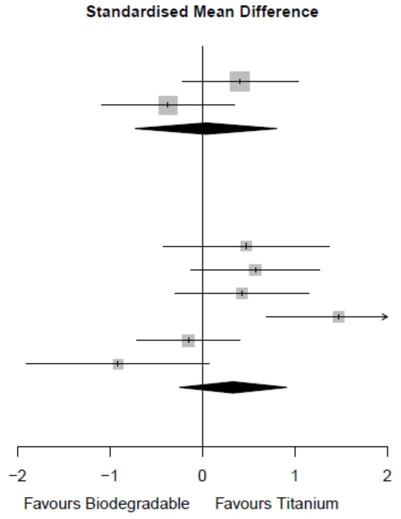

Figure S17: Forest plot of the endpoint mandibular horizontal relapse after mandibular setback stratified by study design (overall follow-up). *RCT, randomised controlled trials; Retrosp. CS, retrospective cohort studies; SMD, standardised mean difference; 95%-CI, 95% confidence interval.*

Study	Biodegradable Mean	Biodegradable SD	Biodegradable Total	Titanium Mean	Titanium SD	Titanium Total	SMD	95%-CI
**Plates and screws**								
Ueki et al. (2005)	2.40	2.89	20	0.70	4.99	20	0.41	[−0.22; 1.04]
Choi et al. (2010)	1.94	0.93	15	1.60	0.58	15	0.43	[−0.30; 1.15]
Lee et al. (2014)	1.89	1.33	8	3.02	1.05	10	−0.91	[−1.90; 0.08]
**Random effects model**			**43**			**45**	**0.06**	**[−0.69; 0.80]**
Heterogeneity: $I^2 = 64\%$, $p = 0.06$								
Test for effect in subgroup: $p = 0.88$								
**Screws only**								
Harada et al. (1997)	1.62	1.28	10	1.05	1.00	10	0.48	[−0.42; 1.37]
Paeng et al. (2012)	0.51	1.23	25	0.75	1.85	25	−0.15	[−0.71; 0.40]
**Random effects model**			**35**			**35**	**0.06**	**[−0.52; 0.64]**
Heterogeneity: $I^2 = 27\%$, $p = 0.24$								
Test for effect in subgroup: $p = 0.84$								
Heterogeneity: $I^2 = 44\%$, $p = 0.13$								
Test for subgroup differences: $\chi^2_1 = 0.00$, df = 1 ($p = 0.99$)								

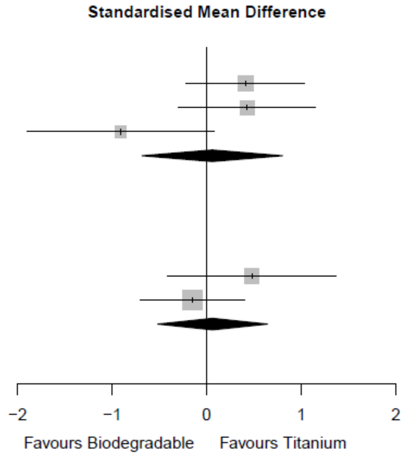

Figure S18: Forest plot of the endpoint mandibular horizontal relapse after mandibular setback of studies including osteosynthesis by plates and screws versus only screws (overall follow-up). *SMD, standardised mean difference; 95%-CI, 95% confidence interval.*

Study	Biodegradable Mean	SD	Total	Titanium Mean	SD	Total	SMD	95%-CI
**RCT**								
Ueki et al. (2005)	0.10	2.61	20	2.50	2.94	20	−0.85	[−1.50; −0.20]
Park et al. (2010)	1.26	1.52	30	1.85	1.73	10	−0.37	[−1.09; 0.35]
**Random effects model**			**50**			**30**	**−0.63**	**[−1.11; −0.15]**
Heterogeneity: $I^2 = 0\%$, $p = 0.33$								
Test for effect in subgroup: $p = 0.01$								
**Retrosp. CS**								
Harada et al. (1997)	0.37	1.33	10	0.00	0.77	10	0.33	[−0.56; 1.21]
Choi et al. (2010)	0.08	1.38	15	0.10	1.10	15	−0.02	[−0.73; 0.70]
Paeng et al. (2012)	0.71	1.35	25	1.50	1.39	25	−0.57	[−1.13; 0.00]
Lee et al. (2014)	0.83	0.53	8	1.51	1.56	10	−0.53	[−1.48; 0.42]
**Random effects model**			**58**			**60**	**−0.25**	**[−0.66; 0.16]**
Heterogeneity: $I^2 = 16\%$, $p = 0.31$								
Test for effect in subgroup: $p = 0.23$								
Heterogeneity: $I^2 = 16\%$, $p = 0.31$								
Test for subgroup differences: $\chi^2_1 = 1.42$, df = 1 ($p = 0.23$)								

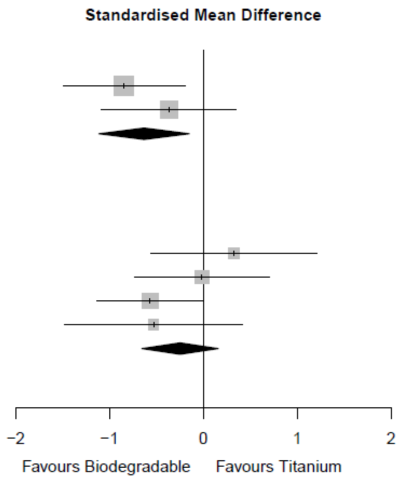

Figure S19: Forest plot of the endpoint mandibular vertical relapse after mandibular setback stratified by study design (overall follow-up). *RCT, randomised controlled trials; Retrosp. CS, retrospective cohort studies; SMD, standardised mean difference; 95%-CI, 95% confidence interval.*

Study	Biodegradable Mean	SD	Total	Titanium Mean	SD	Total	SMD	95%-CI
**Plates and screws**								
Ueki et al. (2005)	0.10	2.61	20	2.50	2.94	20	−0.85	[−1.50; −0.20]
Choi et al. (2010)	0.08	1.38	15	0.10	1.10	15	−0.02	[−0.73; 0.70]
Lee et al. (2014)	0.83	0.53	8	1.51	1.56	10	−0.53	[−1.48; 0.42]
**Random effects model**			**43**			**45**	**−0.47**	**[−0.99; 0.05]**
Heterogeneity: $I^2 = 30\%$, $p = 0.24$								
Test for effect in subgroup: $p = 0.07$								
**Screws only**								
Harada et al. (1997)	0.37	1.33	10	0.00	0.77	10	0.33	[−0.56; 1.21]
Paeng et al. (2012)	0.71	1.35	25	1.50	1.39	25	−0.57	[−1.13; 0.00]
**Random effects model**			**35**			**35**	**−0.19**	**[−1.05; 0.68]**
Heterogeneity: $I^2 = 64\%$, $p = 0.10$								
Test for effect in subgroup: $p = 0.67$								
Heterogeneity: $I^2 = 32\%$, $p = 0.21$								
Test for subgroup differences: $\chi^2_1 = 0.31$, df = 1 ($p = 0.58$)								

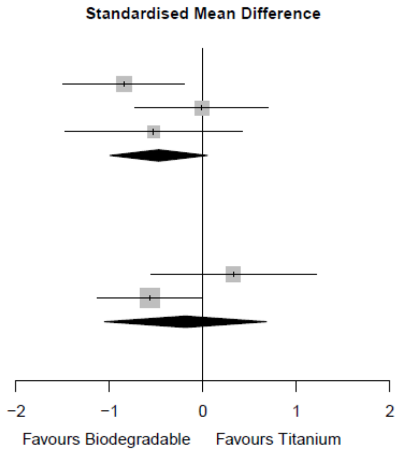

Figure S20: Forest plot of the endpoint mandibular vertical relapse after mandibular setback of studies including osteosynthesis by plates and screws versus only screws (overall follow-up). *SMD, standardised mean difference; 95%-CI, 95% confidence interval.*

Study	Biodegradable Mean	SD	Total	Titanium Mean	SD	Total	SMD	95%-CI
**Retrosp. CS**								
Landes et al. (2006 & 2007)	5.32	7.69	28	8.20	9.60	21	−0.33	[−0.90; 0.24]
Ballon et al. (2012)	4.55	3.52	20	10.63	9.47	15	−0.88	[−1.59; −0.18]
Lee et al. (2014)	0.33	0.85	8	1.39	0.48	10	−1.51	[−2.60; −0.43]
**Random effects model**			**56**			**46**	**−0.79**	**[−1.40; −0.17]**

Heterogeneity: $I^2 = 50\%$, $p = 0.13$
Test for effect in subgroup: $p = 0.01$

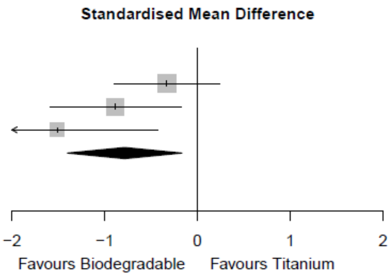

Figure S21: Forest plot of the endpoint mandibular angular relapse after mandibular clockwise rotation stratified by study design (overall follow-up). *Retrosp. CS, retrospective cohort studies; SMD, standardised mean difference; 95%-CI, 95% confidence interval.*

Study	Biodegradable Mean	SD	Total	Titanium Mean	SD	Total	SMD	95%-CI
**RCT**								
Ueki et al. (2005)	6.70	2.53	20	2.60	2.33	20	1.65	[0.92; 2.38]
Park et al. (2010)	5.01	4.61	30	2.30	4.10	10	0.59	[−0.14; 1.32]
**Random effects model**			**50**			**30**	**1.12**	**[0.08; 2.16]**
Heterogeneity: $I^2 = 75\%$, $p = 0.04$								
Test for effect in subgroup: $p = 0.03$								
**Retrosp. CS**								
Landes et al. (2006 & 2007)	6.86	5.03	13	4.20	5.90	9	0.47	[−0.39; 1.34]
Ballon et al. (2012)	6.36	4.86	14	5.00	6.53	13	0.23	[−0.53; 0.99]
**Random effects model**			**27**			**22**	**0.34**	**[−0.23; 0.91]**
Heterogeneity: $I^2 = 0\%$, $p = 0.68$								
Test for effect in subgroup: $p = 0.25$								

Heterogeneity: $I^2 = 64\%$, $p = 0.04$
Test for subgroup differences: $\chi^2_1 = 1.68$, df = 1 ($p = 0.19$)

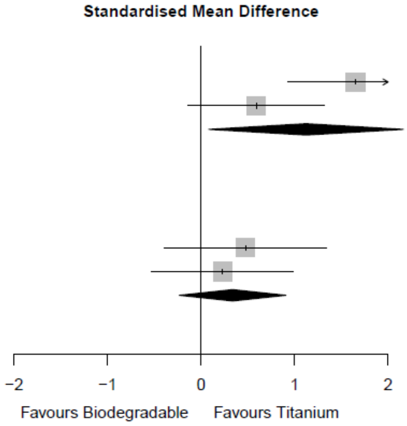

Figure S22: Forest plot of the endpoint mandibular angular relapse after mandibular counter clockwise rotation stratified by study design (overall follow-up). *RCT, randomised controlled trials; Retrosp. CS, retrospective cohort studies; SMD, standardised mean difference; 95%-CI, 95% confidence interval.*

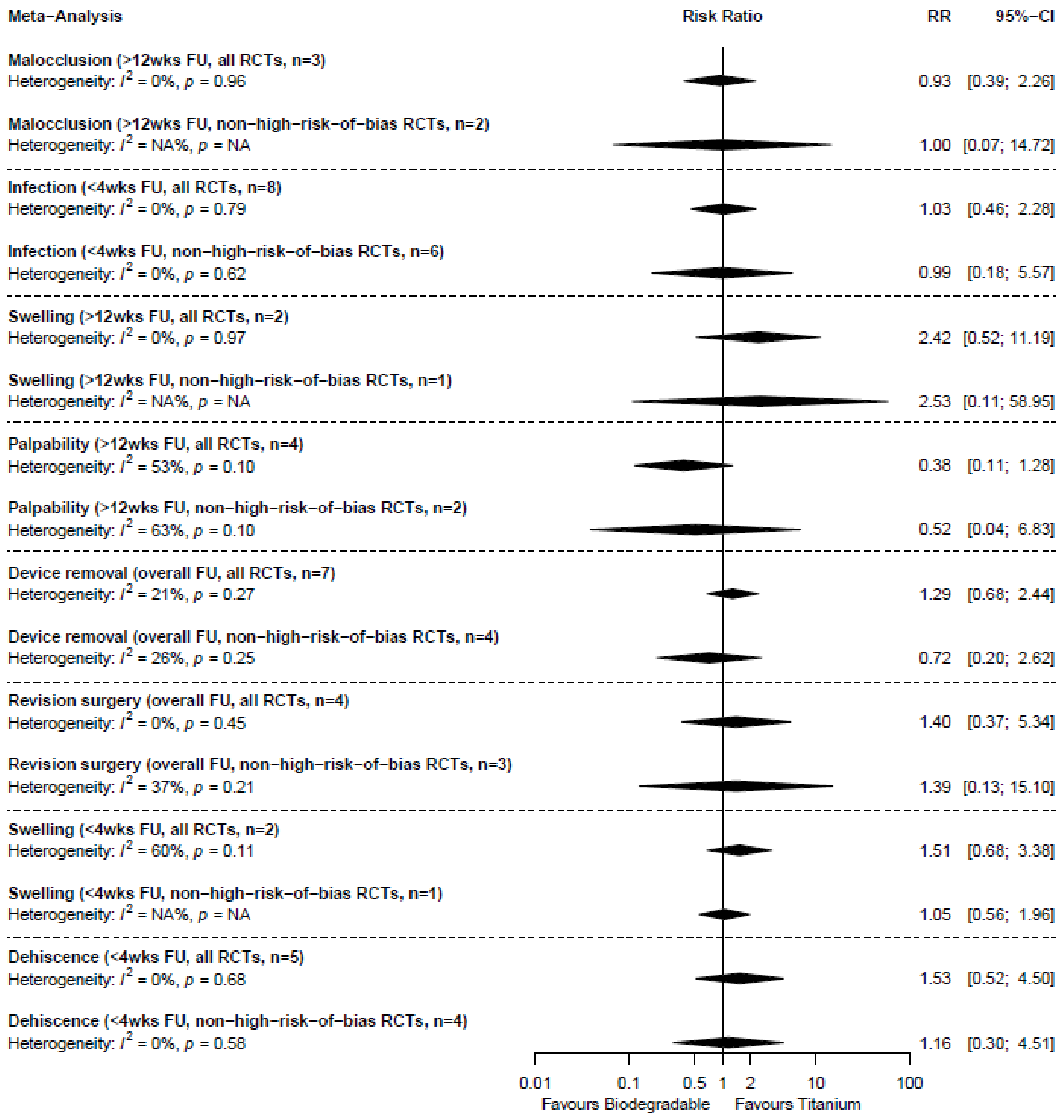

Figure S23: Sensitivity analysis with summary risk ratios for all pooled outcomes according to the inclusion of all randomized controlled trials and non-high-risk-of-bias RCTs only. *RCT, randomised controlled trials; RR, risk ratio; 95%-CI, 95% confidence interval.*

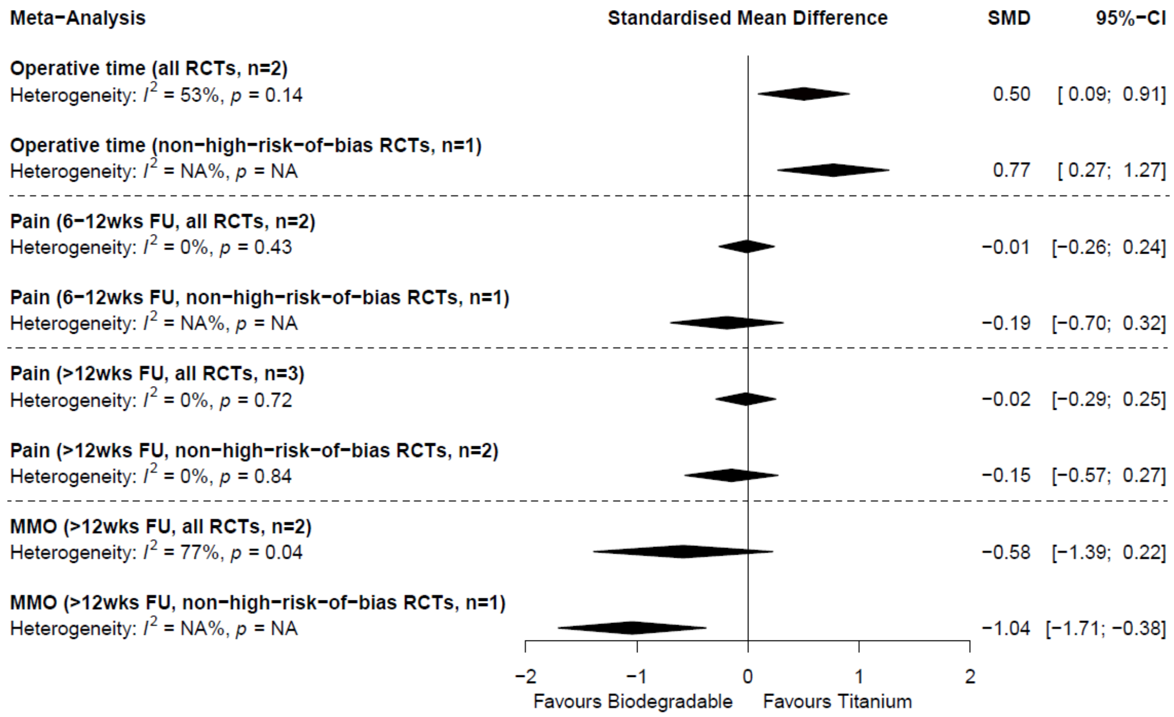

Figure S24: Sensitivity analysis with summary standardized mean differences for all pooled outcomes according to the inclusion of all randomized controlled trials and non-high-risk-of-bias RCTs only. *RCT, randomised controlled trials; SMD, standardised mean difference; 95%-CI, 95% confidence interval.*

References

[1] Matthews NS, Khambay BS, Ayoub AF, Koppel D, Wood G. Preliminary assessment of skeletal stability after sagittal split mandibular advancement using a bioresorbable fixation system. Br J Oral Maxillofac Surg 2003;41:179–84. https://doi.org/10.1016/S0266-4356(03)00048-2.

[2] Cheung LK, Yip IHS, Chow RLK. Stability and morbidity of Le Fort I osteotomy with bioresorbable fixation: a randomized controlled trial. Int J Oral Maxillofac Surg 2008;37:232–41. https://doi.org/10.1016/j.ijom.2007.09.169.

[3] Stockmann P, Böhm H, Driemel O, Mühling J, Pistner H. Resorbable versus titanium osteosynthesis devices in bilateral sagittal split ramus osteotomy of the mandible - The results of a two centre randomised clinical study with an eight-year follow-up. J Cranio-Maxillofacial Surg 2010;38:522–8. https://doi.org/10.1016/j.jcms.2010.01.002.

[4] Tuovinen V, Suuronen R, Teittinen M, Nurmenniemi P. Comparison of the stability of bioabsorbable and titanium osteosynthesis materials for rigid internal fixation in orthognathic surgery. A prospective randomized controlled study in 101 patients with 192 osteotomies. Int J Oral Maxillofac Surg 2010;39:1059–65. https://doi.org/10.1016/j.ijom.2010.07.012.

[5] Landes CA, Ballon A. Skeletal stability in bimaxillary orthognathic surgery: P(L/DL)LA-resorbable versus titanium osteofixation. Plast Reconstr Surg 2006;118:703–21. https://doi.org/10.1097/01.prs.0000232985.05153.bf.

[6] Paeng JY, Hong J, Kim CS, Kim MJ. Comparative study of skeletal stability between bicortical resorbable and titanium screw fixation after sagittal split ramus osteotomy for mandibular prognathism. J Cranio-Maxillofacial Surg 2012;40:660–4. https://doi.org/10.1016/j.jcms.2011.11.001.

[7] Ahmed W, Bukhari SGA, Janjua OS, Luqman U, Shah I, W. A, et al. Bioresorbable versus titanium plates for mandibular fractures. J Coll Physicians Surg Pakistan 2013;23:480–3. https://doi.org/07.2013/JCPSP.480483.

[8] Arshad R, Muddassar M, Rabbani M, Ahmed S, Durrani AU, Gulfam F. Comparative Study of Titanium Micro Plates Versus Bioresorbable Plates In The Osteosynthesis of Isolated Zygomatic Bone Fractures. Ann Dent Spec 2019;7:4–10.

[9] Arya S, Bhatt K, Bhutia O, Roychoudhury A. Efficacy of bioresorbable plates in the osteosynthesis of linear mandibular fractures. Natl J Maxillofac Surg 2020;11:98. https://doi.org/10.4103/njms.njms_54_19.

[10] Bekal M, Brijesh K, Suresha KR. Non-randomised controlled trial comparing the effectiveness of bioresorbable plates versus titanium miniplates in the management of mandibular fractures under general anaesthesia. J Evol Med Dent Sci 2017;6:6286–92.

[11] Bell RB, Kindsfater CS. The use of biodegradable plates and screws to stabilize facial fractures. J Oral Maxillofac Surg 2006;64:31–9. https://doi.org/10.1016/j.joms.2005.09.010.

[12] Bhatt K, Roychoudhury A, Bhutia O, Trikha A, Seith A, Pandey RM. Equivalence randomized controlled trial of bioresorbable versus titanium miniplates in treatment of mandibular fracture: A pilot study. J Oral Maxillofac Surg 2010;68:1842–8. https://doi.org/10.1016/j.joms.2009.09.005.

[13] Bhatt K, Arya S, Bhutia O, Pandey S, Roychoudhury A. Retrospective study of mandibular angle fractures treated with three different fixation systems. Natl J Maxillofac Surg 2015;6:31. https://doi.org/10.4103/0975-5950.168229.

[14] Böhm H, Pistner H, Barth T, Reuther J, Mühling J. Bioresorbierbare Schrauben im Vergleich zu Titanschrauben für die Osteosynthese nach sagittaler Spaltung des Unterkiefers - Eine prospektive, randomisierte, kontrollierte klinische Studie. Biomed Tech 1998;Band 43 ·:4–7.

[15] Bouletreau P, Jurdic P, Mazoorana M, Breton P, Freidel M. Evaluation of tissue response to resorbable osteosynthesis materials in maxillofacial surgery. Rev Stomatol Chir Maxillofac 2005;106:316–20.

[16] Burlini D, Conti G, Amadori F, Bardellini E, De Giuli C. Management of paediatric maxillofacial

fractures: Conventional methods and resorbable materials. Eur J Paediatr Dent 2015;16:24–8.

[17] Champy M, Blez P, Kahn J. [Osteosynthesis using resorbable plates in maxillofacial surgery - hopes and disappointments]. Chirurgie 1992;118:596–600.

[18] Wang JK, Eun SC, Heo CY, Baek RM, Minn KW. The Result of Mandible Fracture Fixations with Biodegradable Materials. J Korean Cleft Palate-Craniofacial Assoc 2013;9:45–50.

[19] Fakourand SR, Ladez MAR. Evaluation of the use of Self-reinforced Absorbable versus Metallic Plates and Screws in the Fractures of Symphysis and Parasymphysis Area. Life Sci Journal-Acta Zhengzhou Univ Overseas Ed 2012;9:1538–42.

[20] Taylan Filinte G, Akan IM, Aycicek Cardak GN, Ozkaya Mutlu O, Akoz T. The Dilemma Of Pediatric Mandible Fractures: Resorbable Or Metallic Plates? Turkish J Trauma Emerg Surg 2015;21:3–7. https://doi.org/10.5505/tjtes.2015.23922.

[21] Fuente del Campo A, Pohjonen T, Tormala P, Waris T. Fixation of horizontal maxillary osteotomies with biodegradable self-reinforced absorbable polylactide plates: preliminary results. Eur J Plast Surg 1996;19:7–9. https://doi.org/10.1007/BF00209782.

[22] Hashiba Y, Ueki K, Marukawa K, Shimada M, Yoshida K, Shimizu C, et al. A comparison of lower lip hypoesthesia measured by trigeminal somatosensory-evoked potential between different types of mandibular osteotomies and fixation. Oral Surgery, Oral Med Oral Pathol Oral Radiol Endodontology 2007;104:177–85. https://doi.org/10.1016/j.tripleo.2006.11.038.

[23] Ho MW, Boyle MA, Cooper JC, Dodd MD, Richardson D. Surgical complications of segmental le Fort i osteotomy. Br J Oral Maxillofac Surg 2011;49:562–6. https://doi.org/10.1016/j.bjoms.2010.09.018.

[24] Hwang DSD, Lee HGH, Shin SSH, Kim UK. Evaluation of Intersegmental Displacement After Mandibular Setback Split Ramus Osteotomy Using Modified L-Shaped Monocortical Plate: Cone-Beam Computed Tomography Superimposition. J Craniofac Surg 2017;00:1–6. https://doi.org/10.1097/SCS.0000000000004161.

[25] Iatrou I, Theologie-Lygidakis N, Tzerbos F. Surgical protocols and outcome for the treatment of maxillofacial fractures in children: 9 years' experience. J Cranio-Maxillofacial Surg 2010;38:511–6. https://doi.org/10.1016/j.jcms.2010.02.008.

[26] Illi O, Sailer H, Stauffer U. [Preliminary of biodegradable osteosynthesis in craniofacial surgery of infants and children]. Chir Pédiatr 1989;30:284–7.

[27] M.J. I, V.L. S. Resorbable internal fixation in pediatric cranial base surgery. Laryngoscope 2002;112:1897–901.

[28] Janickova M, Statelova D, Mikuskova K, Jesenak M, Malachovsky I. Biodegradable versus

titanium plates and screws for paediatric facial skeleton fractures. Bratislava Med J 2018;119:554–9.

[29] Kallela I. [Rigid internal fixation of the mandible using biodegradable polylactide screws and metal screws a clinical and experimental study]. Ann Chir Gynaecol 1999;88:330–1.

[30] Kang IG, Jung JH, Kim ST, Choi JY, Sykes JM. Comparison of titanium and biodegradable plates for treating midfacial fractures. J Oral Maxillofac Surg 2014;72:762.e1-762.e4. https://doi.org/10.1016/j.joms.2013.12.020.

[31] Kim DY, Sung IY, Cho YC, Park E ji, Son JH. Bioabsorbable plates versus metal miniplate systems for use in endoscope-assisted open reduction and internal fixation of mandibular subcondylar fractures. J Cranio-Maxillofacial Surg 2018;46:398–412. https://doi.org/10.1016/j.jcms.2017.12.026.

[32] Kobayashi M, Nakajima T, Yuu. [Clinical cases using absorbable plates in cranio-maxillo-facial surgery]. Keisei-Geka 2004;47:1079.

[33] Kretschmer WB, Baciut G, Baciut M, Zoder W, Wangerin K. Transverse stability of 3-piece Le Fort I osteotomies. J Oral Maxillofac Surg 2011;69:861–9. https://doi.org/10.1016/j.joms.2010.05.024.

[34] Landes CA, Ballon A, Tran A, Ghanaati S, Sader R. Segmental stability in orthognathic surgery: Hydroxyapatite/Poly-l-lactide osteoconductive composite versus titanium miniplate osteosyntheses. J Cranio-Maxillofacial Surg 2014;42:930–42. https://doi.org/10.1016/j.jcms.2014.01.013.

[35] Landes C, Hoefer S, Richards T, Walcher F, Sader R. Perspectives of patients about bioabsorbable internal fixation for maxillofacial fractures. Ann Maxillofac Surg 2015;5:185. https://doi.org/10.4103/2231-0746.175769.

[36] Lee H-B Bin, Oh J-SS, Kim S-GG, Kim H-KK, Moon S-YY, Kim Y-KK, et al. Comparison of titanium and biodegradable miniplates for fixation of mandibular fractures. J Oral Maxillofac Surg 2010;68:2065–9. https://doi.org/10.1016/j.joms.2009.08.004.

[37] Lee GT, Jung HD, Kim SY, Park HS, Jung YS. The stability following advancement genioplasty with biodegradable screw fixation. Br J Oral Maxillofac Surg 2014;52:363–8. https://doi.org/10.1016/j.bjoms.2013.12.009.

[38] Lee JH, Kim SM, Lee BK, Jeon JH, Kim MJ. 3D vector analysis of mandibular condyle stability in mandibular setback surgery with bicortical bioabsorbable screw fixation. J Cranio-Maxillofacial Surg 2014;42:e105–10. https://doi.org/10.1016/j.jcms.2013.07.005.

[39] Leno MB, Liu SY, Chen CT, Liao HT. Comparison of functional outcomes and patient-reported satisfaction between titanium and absorbable plates and screws for fixation of mandibular

fractures: A one-year prospective study. J Cranio-Maxillofacial Surg 2017;45:704–9. https://doi.org/10.1016/j.jcms.2017.01.034.

[40] Leonhardt H, Demmrich A, Mueller A, Mai R, Loukota R, Eckelt U. INION®compared with titanium osteosynthesis: a prospective investigation of the treatment of mandibular fractures. Br J Oral Maxillofac Surg 2008;46:631–4. https://doi.org/10.1016/j.bjoms.2008.04.021.

[41] Lim H-Y, Jung C-H, Kim S-Y, Cho J-Y, Ryu J-Y, Kim H-M. Comparison of resorbable plates and titanium plates for fixation stability of combined mandibular symphysis and angle fractures. J Korean Assoc Oral Maxillofac Surg 2014;40:285. https://doi.org/10.5125/jkaoms.2014.40.6.285.

[42] Liu Y, Wang Y, Hu W. [Analysis of clinical efficacy and complications of titanium plate internal fixation for patients with orbital fracture]. Int Eye Sci 2016;16:1325–8.

[43] Mahmoud SM, Liao H-TT, Chen C-TT. Aesthetic and Functional Outcome of Zygomatic Fractures Fixation Comparison With Resorbable Versus Titanium Plates. Ann Plast Surg 2016;76:S85–90. https://doi.org/10.1097/SAP.0000000000000700.

[44] Menon S, Chowdhury SKR. Evaluation of bioresorbable vis-à-vis titanium plates and screws for craniofacial fractures and osteotomies. Med J Armed Forces India 2007;63:331–3. https://doi.org/10.1016/S0377-1237(07)80008-6.

[45] Menon S, Choudhury CSR. Resorbable Implants in Maxillofacial Surgery: A Reality Check. J Maxillofac Oral Surg 2012;11:132–7. https://doi.org/10.1007/s12663-011-0177-1.

[46] Netto HD, Olate S, Rodriguez-Chesse J, Kluppel L, de Moraes M, Mazzonetto R. Selection of osteosynthesis in maxillary reconstruction with iliac crest bone graft. Rev Kiru 2013;10:161–5.

[47] Obwegeser J. Osteosynthesis using biodegradable (PDS II) in Le Fort I-osteotomy without postoperative intermaxillary fixation. J Cranio-Maxillofacial Surg 1994;22:129–37.

[48] Park WJ, Shin HK. A Comparative Study of the Subjective Symptoms of Bioabsorbable and Metallic Osteofixation System in Zygomatic Bone Fracture. Arch Plast Surg 2005;32:227–30.

[49] C.H. P, H.S. K, J.H. L, S.M. H, Y.G. K, O.J. L, et al. Resorbable skeletal fixation systems for treating maxillofacial bone fractures. Arch Otolaryngol - Head Neck Surg 2011;137:125–9. https://doi.org/10.1001/archoto.2010.241.

[50] Pistner H, Muehling J, Reuther J. [Absorbable materials for osteosynthesis in craniofacial surgery]. Fortschr Kiefer Gesichtschir 1991;36:77–9.

[51] Qiu XD. Effects of bioabsorbable miniplate versus miniature titanium fixation system on the stability of mandibular fractures. Chinese J Tissue Eng Res 2015;19:6155–60. https://doi.org/10.3969/j.issn.2095-4344.2015.38.016.

[52] Stuck BA, Heller T. [Implant materials for the internal fixation of midfacial fractures]. HNO

2011;59:1088–92. https://doi.org/10.1007/s00106-011-2386-9.

[53] Sukegawa S, Kanno T, Nagano D, Shibata A, Sukegawa-Takahashi Y, Furuki Y. The Clinical Feasibility of Newly Developed Thin Flat-Type Bioresorbable Osteosynthesis Devices for the Internal Fixation of Zygomatic Fractures: Is There a Difference in Healing Between Bioresorbable Materials and Titanium Osteosynthesis? J Craniofac Surg 2016;27:2124–9. https://doi.org/10.1097/SCS.0000000000003147.

[54] Tan W, Niu F, Yu B, Gui L. Feasibility of absorbable plates and screws for fixation in reduction malarplasty with L-shaped osteotomy. J Craniofac Surg 2011;22:546–50. https://doi.org/10.1097/SCS.0b013e318208bb41.

[55] Tripathi N, Goyal M, Mishra B, Dhasmana S. Zygomatic complex fracture: A comparative evaluation of stability using titanium and bio-resorbable plates as one point fixation. Natl J Maxillofac Surg 2013;4:181. https://doi.org/10.4103/0975-5950.127648.

[56] Ueki K, Miyazaki M, Okabe K, Mukozawa A, Marukawa K, Moroi A, et al. Assessment of bone healing after Le Fort I osteotomy with 3-dimensional computed tomography. J Cranio-Maxillofacial Surg 2011;39:237–43. https://doi.org/10.1016/j.jcms.2010.06.008.

[57] Ueki K, Moroi A, Iguchi R, Kosaka A, Ikawa H, Yoshizawa K. Changes in the computed tomography (pixel) value of mandibular ramus bone and fixation screws after sagittal split ramus osteotomy. Int J Oral Maxillofac Surg 2015;44:1337–45. https://doi.org/10.1016/j.ijom.2015.06.010.

[58] Ueki K, Yoshizawa K, Moroi A, Hotta A, Tsutsui T, Fukaya K, et al. Modified hybrid fixation using absorbable plate and screw for mandibular advancement surgery. J Cranio-Maxillofacial Surg 2017;45:1788–93. https://doi.org/10.1016/j.jcms.2017.08.006.

[59] Wittwer G, Adeyemo WL, Yerit K, Voracek M, Turhani D, Watzinger F, et al. Complications after zygoma fracture fixation: Is there a difference between biodegradable materials and how do they compare with titanium osteosynthesis? Oral Surgery, Oral Med Oral Pathol Oral Radiol Endodontology 2006;101:419–25. https://doi.org/10.1016/j.tripleo.2005.07.026.

[60] Wu CM, Chen YA, Liao HT, Chen C hao, Pan CH, Chen CT. Surgical treatment of isolated zygomatic fracture: Outcome comparison between titanium plate and bioabsorbable plate. Asian J Surg 2017;41:370–6. https://doi.org/10.1016/j.asjsur.2017.03.003.

[61] Zheng C. [The use of absorbable plates in fixation of maxillofacial fractures]. Shang Hai Kou Qiang Yi Xue 2001;10:89–90.

[62] Norholt SE, Pedersen TK, Jensen J. Le Fort I miniplate osteosynthesis: a randomized, prospective study comparing resorbable PLLA/PGA with titanium. Int J Oral Maxillofac Surg 2004;33:245–52. https://doi.org/10.1006/ijom.2003.0505.

[63] Cheung LK, Chow LK, Chiu WK. A randomized controlled trial of resorbable versus titanium fixation for orthognathic surgery. Oral Surgery, Oral Med Oral Pathol Oral Radiol Endodontology 2004;98:386–97. https://doi.org/10.1016/j.tripleo.2004.02.069.

[64] Ueki K, Nakagawa K, Marukawa K, Takazakura D, Shimada M, Takatsuka S, et al. Changes in condylar long axis and skeletal stability after bilateral sagittal split ramus osteotomy with poly-l-lactic acid or titanium plate fixation. Int J Oral Maxillofac Surg 2005;34:627–34. https://doi.org/10.1016/j.ijom.2005.02.013.

[65] Park J, Park Y. Postoperative Stability of Fixation with Absorbables in Simultaneous Maxillomandibular Orthognathic Surgery. Maxillofac Plast Reconstr Surg 2010;32:126–31.

[66] Buijs GJ, Van Bakelen NB, Jansma J, de Visscher JGAM, Hoppenreijs TJM, Bergsma JE, et al. A randomized clinical trial of biodegradable and titanium fixation systems in maxillofacial surgery. J Dent Res 2012;91:299–304. https://doi.org/10.1177/0022034511434353.

[67] Yoshioka I, Igawa K, Nagata J, Yoshida M, Ogawa Y, Ichiki T, et al. Comparison of material-related complications after bilateral sagittal split mandibular setback surgery: Biodegradable versus titanium miniplates. J Oral Maxillofac Surg 2012;70:919–24. https://doi.org/10.1016/j.joms.2011.02.136.

[68] van Bakelen NB, Buijs GJ, Jansma J, de Visscher JG, Hoppenreijs TJ, Bergsma JE, et al. Comparison of biodegradable and titanium fixation systems in maxillofacial surgery: a two-year multi-center randomized controlled trial. J Dent Res 2013;92:1100–5. https://doi.org/10.1177/0022034513508953 [doi].

[69] Yu S, Bloomquist D. Can resorbable screws effectively be used in fixating bilateral sagittal split osteotomies for mandibular advancement? a randomized controlled trial. J Oral Maxillofac Surg 2014;72:2273–7. https://doi.org/10.1016/j.joms.2014.04.033.

[70] Van Bakelen NB, Vermeulen KM, Buijs GJ, Jansma J, De Visscher JGAM, Hoppenreijs TJM, et al. Cost-effectiveness of a biodegradable compared to a titanium fixation system in maxillofacial surgery: A multicenter randomized controlled trial. PLoS One 2015;10:1–16. https://doi.org/10.1371/journal.pone.0130330.

[71] Gareb B, Van Bakelen NB, Buijs GJ, Jansma J, De Visscher JGAM, Hoppenreijs TJM, et al. Comparison of the long-term clinical performance of a biodegradable and a titanium fixation system in maxillofacial surgery: A multicenter randomized controlled trial. PLoS One 2017;12:1–12. https://doi.org/10.1371/journal.pone.0177152.

[72] Ferretti C, Reyneke JP. Mandibular, sagittal split osteotomies fixed with biodegradable or titanium screws: A prospective, comparative study of postoperative stability. Oral Surg Oral Med Oral Pathol Oral Radiol Endod 2002;93:534–7. https://doi.org/10.1067/moe.2002.124091.

[73] Dhol WS, Reyneke JP, Tompson B, Sándor GKB. Comparison of titanium and resorbable

copolymer fixation after Le Fort I maxillary impaction. Am J Orthod Dentofac Orthop 2008;134:67–73. https://doi.org/10.1016/j.ajodo.2006.04.049.

[74] Van Bakelen NB, Boermans BDA, Buijs GJ, Jansma J, Pruim GJ, Hoppenreijs TJM, et al. Comparison of the long-term skeletal stability between a biodegradable and a titanium fixation system following BSSO advancement - A cohort study based on a multicenter randomised controlled trial. Br J Oral Maxillofac Surg 2014;52:721–8. https://doi.org/10.1016/j.bjoms.2014.06.014.

[75] Harada K, Enomoto S. Stability after surgical correction of mandibular prognathism using the sagittal split ramus osteotomy and fixation with poly-L-lactic acid (PLLA) screws. J Oral Maxillofac Surg 1997;55:464–9. https://doi.org/10.1016/S0278-2391(97)90691-1.

[76] Costa F, Robiony M, Zorzan E, Zerman N, Politi M. Stability of skeletal class III malocclusion after combined maxillary and mandibular procedures: Titanium versus resorbable plates and screws for maxillary fixation. J Oral Maxillofac Surg 2006;64:642–51. https://doi.org/10.1016/j.joms.2005.11.043.

[77] Turvey TA, Bell RB, Phillips C, Proffit WR. Self-reinforced biodegradable screw fixation compared with titanium screw fixation in mandibular advancement. J Oral Maxillofac Surg 2006;64:40–6. https://doi.org/10.1016/j.joms.2005.09.011.

[78] Ueki K, Marukawa K, Shimada M, Nakagawa K, Alam S, Yamamoto E. Maxillary stability following Le Fort I osteotomy in combination with sagittal split ramus osteotomy and intraoral vertical ramus osteotomy: A comparative study between titanium miniplate and poly-L-lactic acid plate. J Oral Maxillofac Surg 2006;64:74–80. https://doi.org/10.1016/j.joms.2005.09.015.

[79] Landes C, Ballon A, Sader R. Segment stability in bimaxillary orthognathic surgery after resorbable Poly(L-lactide-co-glycolide) versus titanium osteosyntheses. J Craniofac Surg 2007;18:1216–29. https://doi.org/10.1097/scs.0b013e31814b29df.

[80] Ueki K, Hashiba Y, Marukawa K, Okabe K, Nakagawa K, Alam S, et al. Evaluation of Bone Formation After Sagittal Split Ramus Osteotomy With Bent Plate Fixation Using Computed Tomography. J Oral Maxillofac Surg 2009;67:1062–8. https://doi.org/10.1016/j.joms.2008.11.016.

[81] Ahn YS, Kim SG, Baik SM, Kim BO, Kim HK, Moon SY, et al. Comparative Study Between Resorbable and Nonresorbable Plates in Orthognathic Surgery. J Oral Maxillofac Surg 2010;68:287–92. https://doi.org/10.1016/j.joms.2009.07.020.

[82] Choi B-H, Park S-W, Jang S-M, Son H-N, Park B-C, Son J-H, et al. The study of stability of absorbable internal fixation after mandibular bilateral sagittal split ramal osteotomy. J Korean Assoc Oral Maxillofac Surg 2010;36:255. https://doi.org/10.5125/jkaoms.2010.36.4.255.

[83] Ueki K, Okabe K, Miyazaki M, Mukozawa A, Moroi A, Marukawa K, et al. Skeletal stability after

mandibular setback surgery: Comparisons among unsintered hydroxyapatite/poly-L-lactic acid plate, poly-L-lactic acid plate, and titanium plate. J Oral Maxillofac Surg 2011;69:1464–8. https://doi.org/10.1016/j.joms.2010.06.187.

[84] Ballon A, Laudemann K, Sader R, Landes CA. Segmental stability of resorbable P(L/DL)LA-TMC osteosynthesis versus titanium miniplates in orthognatic surgery. J Cranio-Maxillofacial Surg 2012;40:e408–14. https://doi.org/10.1016/j.jcms.2012.02.014.

[85] Ueki K, Okabe K, Moroi A, Marukawa K, Sotobori M, Ishihara Y, et al. Maxillary stability after le Fort i osteotomy using three different plate systems. Int J Oral Maxillofac Surg 2012;41:942–8. https://doi.org/10.1016/j.ijom.2012.02.023.

[86] Blakey GH, Rossouw E, Turvey TA, Phillips C, Proffit WR, White RP. Are bioresorbable polylactate devices comparable to titanium devices for stabilizing le Fort i advancement? Int J Oral Maxillofac Surg 2014;43:437–44. https://doi.org/10.1016/j.ijom.2013.10.006.

[87] Lee JY, Kim YK, Yun PY, Lee NK, Kim JW, Choi JH. Evaluation of stability after orthognathic surgery with minimal orthodontic preparation: Comparison according to 3 types of fixation. J Craniofac Surg 2014;25:911–5. https://doi.org/10.1097/SCS.0000000000000609.

[88] Ueki K, Ishihara Y, Yoshizawa K, Moroi A, Ikawa H, Iguchi R, et al. Evaluation of bone formation after sagittal split ramus osteotomy using different fixation materials. J Cranio-Maxillofacial Surg 2015;43:710–6. https://doi.org/10.1016/j.jcms.2015.03.009.
